# Cu(II)-Mediated direct ^18^F-dehydrofluorination of phosphine oxides in high molar activity

**DOI:** 10.1186/s41181-023-00234-y

**Published:** 2024-01-06

**Authors:** Xiaoqun Tang, Shengji Lv, Zhaobiao Mou, Xia Liu, Zijing Li

**Affiliations:** 1 State Key Laboratory of Vaccines for Infectious Diseases, Center for Molecular Imaging and Translational Medicine, Xiang An Biomedicine Laboratory, School of Public Health, Xiamen University, Xiamen, 361102 Fujian China; 2 State Key Laboratory of Molecular Vaccinology and Molecular Diagnostics, National Innovation Platform for Industry-Education Integration in Vaccine Research, Xiamen University, Xiamen, 361102 Fujian China

**Keywords:** ^18^F-Labeling, Dehydrofluorination, Cu(II), Phosphine oxide, Molar activity

## Abstract

**Background:**

The ^18^F/^19^F-isotope exchange method employing P(V)-centered prosthetic groups demonstrates advantages in addressing mild one-step aqueous ^18^F-labeling of peptides and proteins. However, the molar activity (A_m_) achieved through isotope exchange remains relatively low, unless employing a high initial activity of [^18^F]F^−^. To overcome this drawback, our work introduces a novel approach through a Cu-mediated direct ^18^F-dehydrofluorination of phosphine oxides. This method leverages the straightforward separation of the ^18^F-labeled product from the phosphine oxide precursors, aiming to primarily increase A_m_.

**Results:**

Through a ^19^F-dehydrofluorination efficiency test, Cu(OAc)_2_ was identified as the optimal oxidative metal salt, exhibiting a remarkable 100% conversion within one hour. Leveraging the straightforward separation of phosphine oxide precursors and phosphinic fluoride products, the A_m_ of an activated ester, [^18^F]**4**, sees an impressive nearly 15-fold increase compared to the ^18^F/^19^F-isotope exchange, with the same initial activity of [^18^F]F^−^. Furthermore, this Cu(II)-mediated ^18^F-dehydrofluorination approach demonstrates tolerance up to 20% solvent water content, which enables the practical radiosynthesis of ^18^F-labeled water-soluble molecules under non-drying conditions.

**Conclusions:**

The direct ^18^F-dehydrofluorination of phosphine oxide prosthetic groups has been successfully accomplished, achieving a high A_m_ via Cu(II)-mediated oxidative addition and reductive elimination.

**Supplementary Information:**

The online version contains supplementary material available at 10.1186/s41181-023-00234-y.

## Background

With a favorable half-life of 109.8 min, widespread availability, and a low maximum positron energy of 634 keV, ^18^F that is typically produced in the form of [^18^F]F^−^ with high initial A_m_, is indispensable for obtaining high-resolution PET images in clinical and research settings (Wängler et al. 2012; Miller et al. 2008; Littich et al. 2012; Coenen et al. 2010; Jacobson et al. 2015; Cai et al. 2008). Aqueous ^18^F-labeling methods, capable of accommodating the excellent solubility of various hydrophilic substrates and [^18^F]F^−^ derived from [^18^O]H_2_O, have the potential to significantly reduce substantial loss of activity and time during the drying and redissolving of [^18^F]F^−^. The ^18^F/^19^F-isotope exchange method, relying on B/Si/P-centered prosthetic groups for ^18^F-labeling, has demonstrated advantages in the one-step aqueous labeling of peptides and a select range of small-molecular tracers (Liu et al. 2014a; Hong et al. 2019; Schirrmacher et al. 2006). High molar activity ^18^F-AMBF_3_-TATE (> 111 GBq/μmol) and ^18^F-SiFA*lin*-TATE (60 ± 7 GBq/μmol) have been achieved by isotope exchange of ^18^F-fluoride with a high starting activity (8–37 GBq) and successfully applied in clinical studies (Liu et al. 2014a; Ilhan et al. 2020). However, attaining such high A_m_ is contingent upon a low precursor loads that may otherwise compromise the radiochemical yield (RCY), and the utilization of high initial-A_m_ [^18^F]F^−^ generated in a system devoid of fluorinated materials (Berridge et al. 2009).

**Scheme 1:**
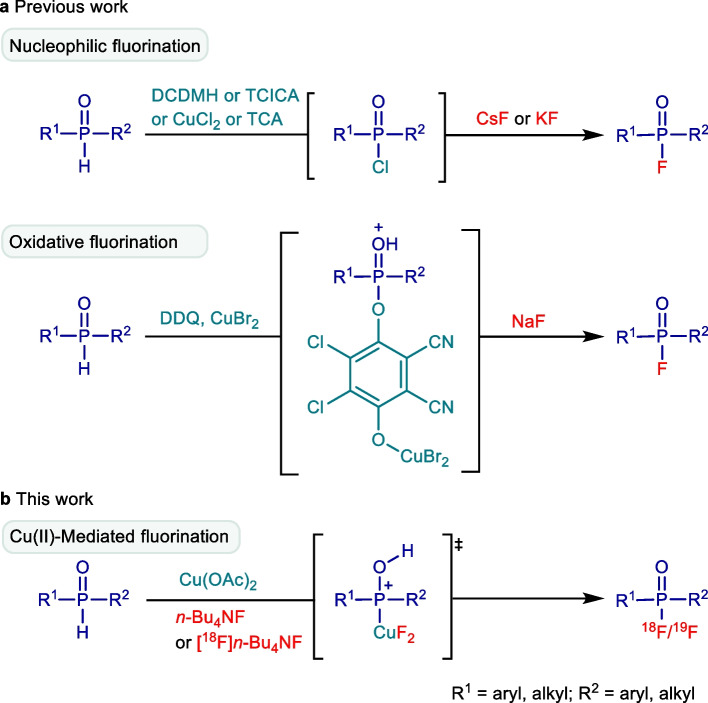
Overview of strategies for dehydrofluorination of phosphine oxides. **a** Previous work: nucleophilic fluorination and oxidative fluorination. **b** This work: Cu(II)-mediated fluorination. DCDMH: 1,3-Dichloro-5,5-dimethylhydantoin; TCICA: 1,3,5-Trichloro-1,3,5-triazinane-2,4,6-trione; TCA: Trichloroacetonitrile; DDQ: 2,3-Dichloro-5,6-dicyano-4-benzoquinone

In 2005, a groundbreaking method for labeling the cholinesterase inhibitor Dimefox (*N,N,N',N'*-tetramethylphosphorodiamidic fluoride) showcased the feasibility of constructing P-^18^F bonds (Studenov et al. 2005). Notably, the initial approach utilized the labeling precursor *N,N,N',N'*-tetramethylphosphorodiamidic chloride, which exhibited poor stability and susceptibility to hydrolysis upon contact with water. Recognizing these drawbacks, we turn our attention to phosphine oxides—an extensively available class of pentavalent phosphine reagents known for their high solubility and stability in aqueous media. These phosphine oxides emerge as promising ^18^F-labeling precursors, displaying an adequate polarity shift after fluorination that facilitates effective separation. Traditionally, the synthesis of organophosphorus fluorides from phosphine oxides involved one-pot-two-step methods, including nucleophilic attacks by F^−^ on intermediates featuring leaving groups such as Cl (Gupta et al. 2008a, b; Bornemann et al. 2021; Purohit et al. 2015), alkyl sulfide (Timperley et al. 2005), phenyl ether (Wang et al. 2021), imidazole (Mou et al. 2021), or oxidative coupling between phosphine hydrogen oxide and NaF (Liu et al. 2014b) (Scheme 1a). Unfortunately, these approaches often necessitated highly toxic, corrosive chlorinating reagents or strong oxidants, making them less amenable to mild ^18^F-labeling in an aqueous phase. An alternative avenue lies in the use of less toxic oxidative simple metal salts, which possess hydrates and are resilient to solvent effects. This approach presents a viable option for the direct dehydrofluorination of phosphine oxides through oxidative addition and reductive elimination.

In this study, a metal-mediated method for the direct radiofluorination of phosphine oxide prosthetic groups is developed (Scheme 1b). Initially, an array of metal salts including Cu(II), Cu(I), Ag(I), Pd(II), Fe(III), Fe(II), Ni(II), Mn(II), Zn(II), and Pt(II) undergoes investigation via a ^19^F-fluorination efficiency test monitored by ^19^F nuclear magnetic resonance (NMR). Subsequent optimization of the ^19^F-dehydrofluorination reaction conditions is conducted, exploring variations in metal salt equivalents, solvents, fluorine sources, and reaction duration. Building upon these findings, the optimization of conditions for the oxidative metal-mediated ^18^F-radiofluorination is pursued, with an additional focus on investigating water resistance properties. Capitalizing on the potential ease of separating the ^18^F-labeled products from the precursors, the achieved A_m_ via this direct ^18^F-dehydrofluorination approach is compared with that obtained through isotope exchange with the same initial activity of [^18^F]F^−^. The proposed mechanism involving oxidative addition and reductive elimination for this direct dehydrofluorination approach is observed through a ^19^F-fluorination conversion test, monitored by ^31^P NMR, incorporating free radical scavengers, organic acids, or organic bases as additives.

## Results

### Synthesis

**Scheme 2:**
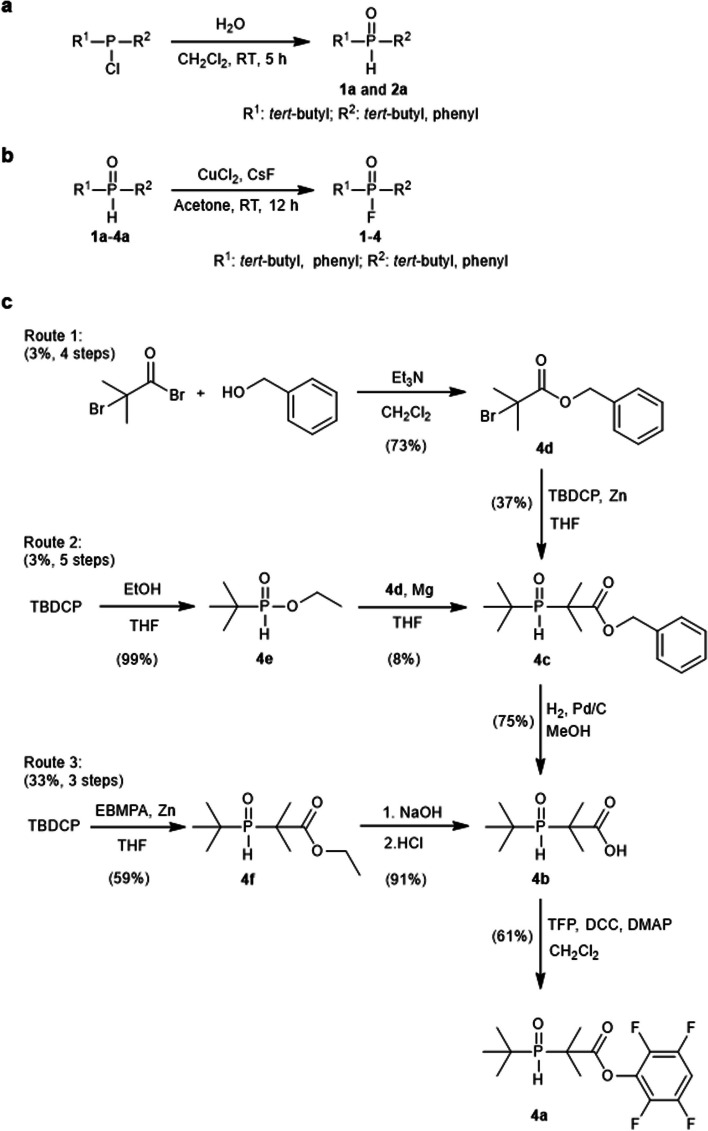
Synthetic routes for phosphine oxides and phosphinic fluorides. **a** Synthetic route of phosphine oxides **1a** and **2a**. **b** Synthetic route of phosphinic fluorides **1**–**4**. **c** Three synthetic routes of phosphine oxides **4a**. DCC: Dicyclohexylcarbodiimide; DMAP: 4-Dimethylaminopyridine. EBMPA: Ethyl 2-bromo-2-methylpropanoate; TBDCP: *tert*-Butyldichlorophosphane; TFP: 2,3,5,6-Tetrafluorophenol

Phosphine oxide substrates **1a** and **2a** were obtained in yields of 97–98% through the reciprocal isomerization of the hydrolysis products of the corresponding chlorophosphane, as illustrated in Scheme 2a (Smoll et al. 2017). Compound **3a** was procured directly from Energy Chemical Co., Ltd. (China), while **4a** was synthesized through three distinct routes, as depicted in Scheme 2c, with yields ranging from 3% to 33%. The fluorination products, referred to as the reference compounds—phosphinic fluorides **1–4**—were prepared from phosphine oxides **1a–4a**, employing CsF as the fluoride source in the presence of CuCl_2_, as outlined in Scheme 2b (Purohit et al. 2015). All synthesized compounds were characterized by nuclear magnetic resonance spectroscopy (^1^H NMR, ^13^C NMR, ^31^P NMR and ^19^F NMR) and mass spectrometry (MS). The chemical purities of these compounds were determined to be > 95% using thin-layer chromatography (TLC), NMR, and high-performance liquid chromatography (HPLC) methods.

### Preliminary screening of oxidative metal salts

**Fig. 1:**
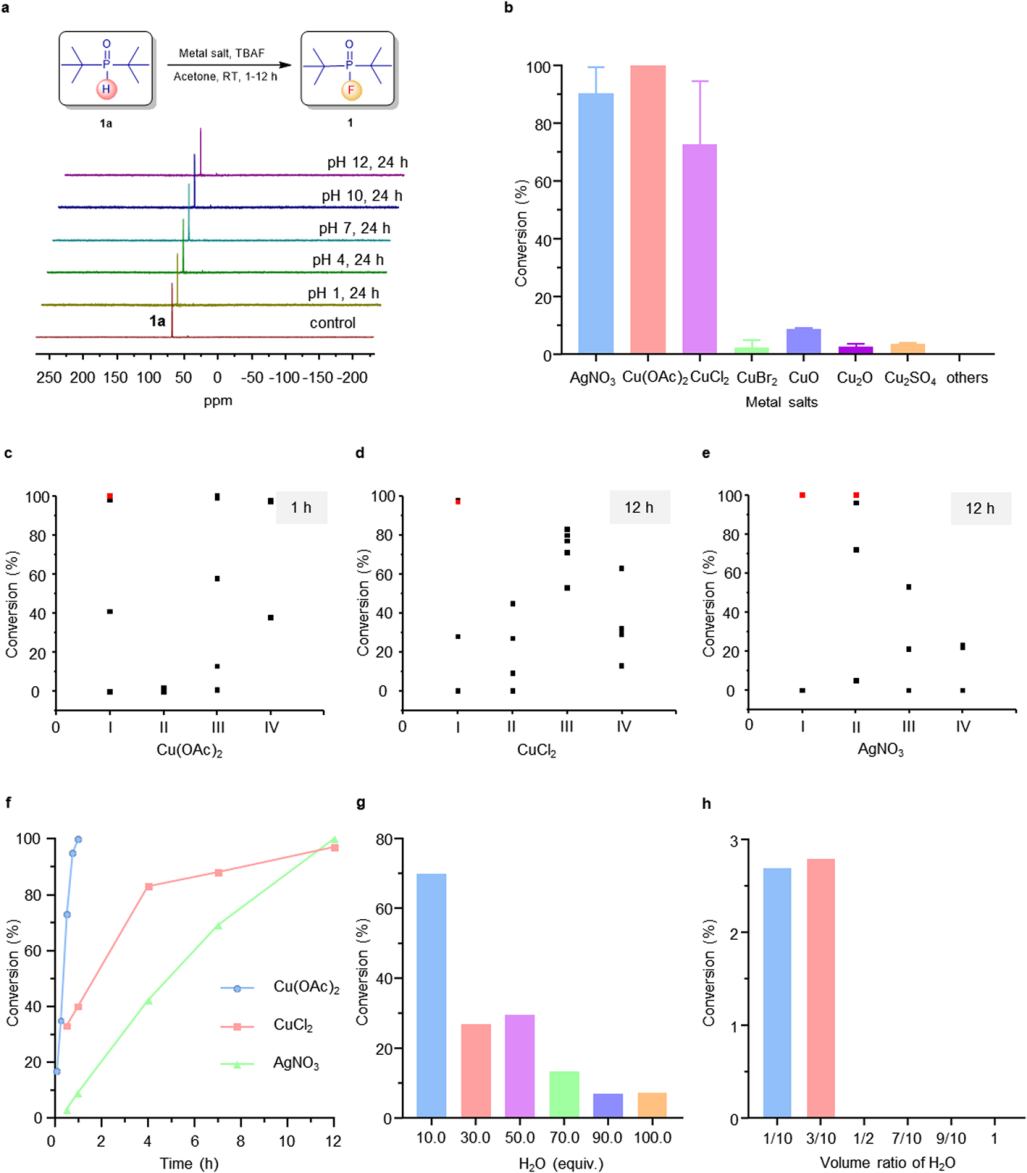
Screening of the oxidative metal salts for direct dehydrofluorination of phosphine oxides.** a** Stability of phosphine oxide **1a** incubated under different pH conditions for 24 h. **b** Screening of metal salts, others: MnCl_2_, FeCl_3_·6H_2_O, FeSO_4_, Fe(OAc)_3_, Fe(OTf)_3_, NiCl_2_, Ni(acac)_2_, Cu(OMs)_2_, Cu powder, CuI, CuCl, ZnCl_2_, ZnBr_2_, Zn(NO_3_)_2_, ZnSO_4_, Zn(OAc)_2_, Zn(OTf)_2_, ZnO, AgTFA, Ag_2_SO_4_, AgOAc, AgOTf, Pd(OAc)_2_, PdCl_2_, Pd(acac)_2_, Pd(dba)_2_, PtCl_2_. **c–e** Optimization of Cu(OAc)_2_, CuCl_2_ and AgNO_3_-mediated fluorination reaction; The conditions of I: **1a** (10 mg), metal salts (0.05–3 equiv.), acetone (0.5 mL), TBAF (2 equiv.); The conditions of II: **1a** (10 mg), metal salts (2 equiv.), acetone (0.5 mL), KF or CsF or NaF or AgF (2 equiv.); The conditions of III: **1a** (10 mg), metal salts (2 equiv.), CH_3_CN or THF or DMF or DMSO or CH_3_OH (0.5 mL), TBAF (2 equiv.); The conditions of IV: **1a** (10 mg), metal salts (2 equiv.), acetone (0.5 mL), TBAF (2 equiv.), TEA or Py or DBU or AcOH (2 equiv.). **f** Comparison of fluorination efficiency mediated by Cu(OAc)_2_, CuCl_2_ and AgNO_3_ under optimal conditions. **g** The effects of water equivalent on conversions in Cu(OAc)_2_-mediated fluorination reaction. Reaction condition: **1a** (10 mg), KF (2 equiv.), Cu(OAc)_2_ (2 equiv.), H_2_O (10–100 equiv.), DMSO (0.4 mL), 25 °C, 12 h. **h** The effects of water volume ratio on conversions in Cu(OAc)_2_-mediated fluorination reaction. Reaction condition: **1a** (10 mg), KF (2 equiv.), Cu(OAc)_2_ (2 equiv.), H_2_O(v): DMSO(v) = 10: 90, 30: 70, 50: 50, 70: 30, 90: 10, 100: 0, 25 °C, 12 h

To assess the stability of phosphine oxides, di-*tert*-butylphosphine oxide **1a** was dissolved in a mixture of water and acetonitrile at varying pH levels (1, 4, 7, 10, 12) and incubated at room temperature for 24 h. Remarkably, **1a** demonstrated robust stability under all tested conditions, as depicted in Fig. 1a. Subsequently, employing compound **1a** as the model substrate and tetrabutylammonium fluoride (TBAF) as the fluorination reagent, a comprehensive screening of oxidative metal salts, including Cu(II), Cu(I), Ag(I), Pd(II), Fe(III), Fe(II), Ni(II), Mn(II), Zn(II), and Pt(II) salts, were conducted under a general reaction formula outlined in Additional file 1: Scheme S1. The results indicated that, in addition to CuCl_2_, which has been reported in the literature to effectively mediate dehydrofluorination (Purohit et al. 2015), AgNO_3_ and Cu(OAc)_2_ (even achieving 100% dehydrofluorination conversion) exhibited outstanding fluorination efficiency (Fig. 1b and Additional file 1: Tables S1–S4). Conversely, dehydrofluorination conversions mediated by other transition metal salts were below 10%, with Pt(II) and Zn(II) metal salts exhibiting 0% dehydrofluorination conversion. Subsequently, the fluorination methods mediated by three metal salts—CuCl_2_, AgNO_3_, and Cu(OAc)_2_—were further optimized with respect to reaction time, reaction solvent, and fluorine source (Fig. 1c–e and Additional file 1: Tables S5–S7). Despite all three salts achieving fluorination yields exceeding 97% after optimization, Cu(OAc)_2_ demonstrated complete conversion in less than one hour, indicating significantly higher fluorination efficiency than the other two metal salts (Fig. 1f and Additional file 1: Tables S8–S10). The water resistance of this fluorination method was also preliminary tested to provide insights for subsequent radiofluorination studies in aqueous media (Fig. 1g, h). Remarkably, a fluorination conversion of 70% was maintained when water was added in 10 equivalents of the substrate. Even with a stoichiometric ratio of water to substrate increased to 100:1, a fluorination conversion of 7% was still observed in the ^31^P NMR spectrum.

### Radiochemistry

**Fig. 2:**
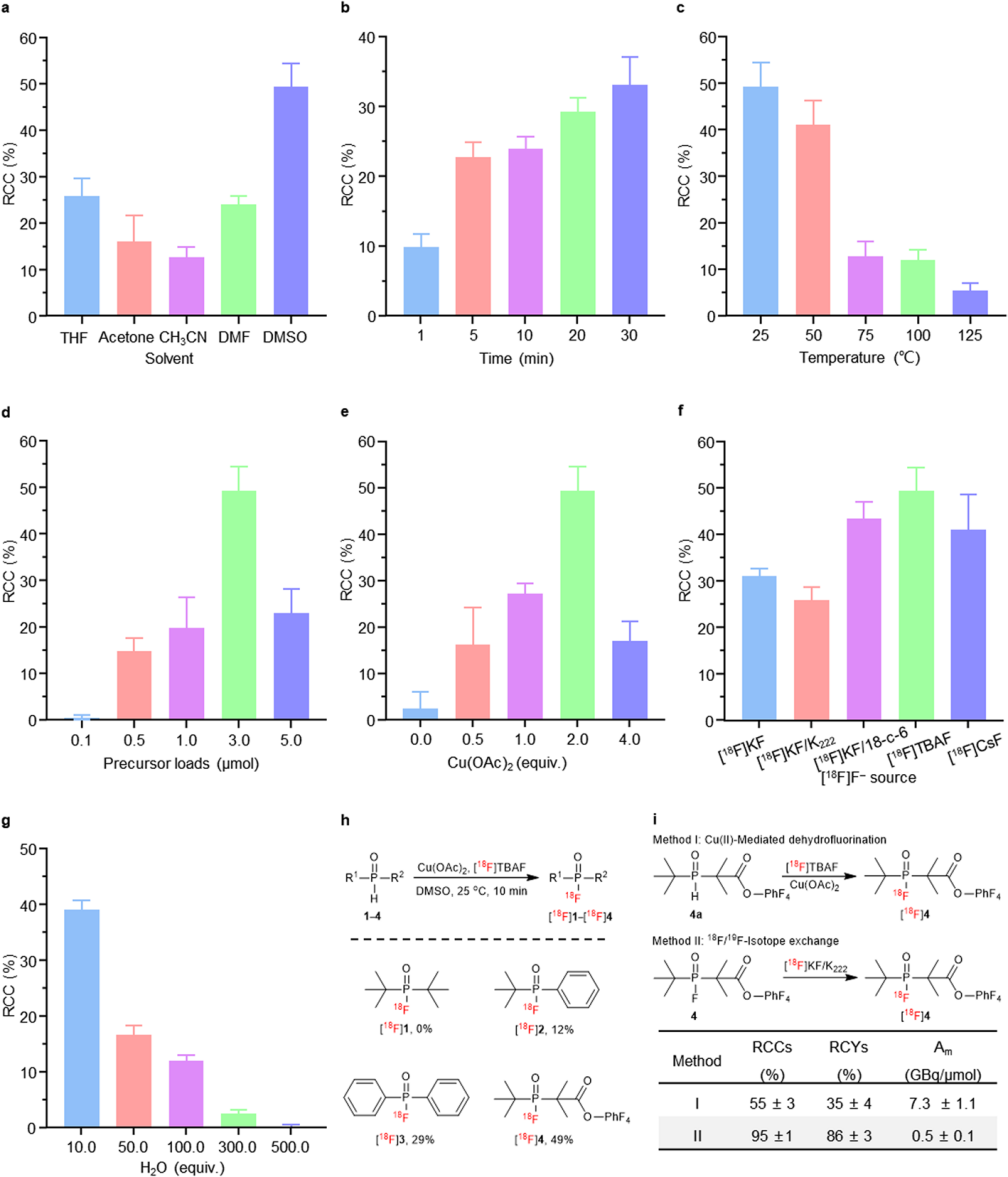
Optimization of ^18^F-fluorination condition for activated ester [^18^F]**4**. **a** The effects of solvent on RCCs; Reaction conditions: precursor **4a** (3 μmol), Cu(OAc)_2_ (2 equiv.), [^18^F]TBAF (2–3 mCi), solvent (200 μL), 25 °C, 10 min. **b** The effects of time on RCCs; Reaction conditions: precursor **4a** (3 μmol), Cu(OAc)_2_ (2 equiv.), [^18^F]TBAF (2–3 mCi), DMF (200 μL), 25 °C, 1–30 min. **c** The effects of temperature on RCCs; Reaction conditions: precursor **4a** (3 μmol), Cu(OAc)_2_ (2 equiv.), [^18^F]TBAF (2–3 mCi), DMSO (200 μL), 25–125 °C, 10 min. **d** The effects of precursor loads on RCCs; Reaction conditions: precursor **4a** (0.1–5 μmol), Cu(OAc)_2_ (2 equiv.), [^18^F]TBAF (2–3 mCi), DMSO (200 μL), 25 °C, 10 min. **e** The effects of equivalents of Cu(OAc)_2_ on RCCs; Reaction conditions: precursor **4a** (3 μmol), Cu(OAc)_2_ (0–4 equiv.), [^18^F]TBAF (2–3 mCi), DMSO (200 μL), 25 °C, 10 min. **f** The effects of [^18^F]F^−^ source on RCCs; Reaction conditions: precursor **4a** (3 μmol), Cu(OAc)_2_ (2 equiv.), [^18^F]F^−^ source (2–3 mCi), DMSO (200 μL), 25 °C, 10 min. **g** The effects of equivalents of H_2_O on RCCs; Reaction conditions: precursor **4a** (3 μmol), Cu(OAc)_2_ (2 equiv.), [^18^F]TBAF (2–3 mCi), DMSO (200 μL), H_2_O (10–500 equiv.), 25 °C, 10 min. **h** Substrate scope for the synthesis of ^18^F-labeled fluorophosphine. Conditions: precursors (3 μmol), Cu(OAc)_2_ (2 equiv.), [^18^F]TBAF (2–3 mCi), DMSO (200 μL), 25 °C, 10 min. **i** The A_m_ of Cu(II)-mediated dehydrofluorination versus ^18^F/^19^F-isotope exchange; Method I: precursor **4a** (3 μmol), Cu(OAc)_2_ (2 equiv.), [^18^F]TBAF (20 mCi), DMSO (200 μL), 25 °C, 10 min. Method II: precursor **4** (3 μmol), [^18^F]KF/K_222_ (20 mCi), CH_3_CN (200 μL), 25 °C, 10 min

The optimization of ^18^F-labeling conditions for this Cu(II)-mediated radiofluorination method was conducted using the activated ester substrate **4a**, a promising ^18^F-synthon, as the model substrate (Additional file 1: Scheme S3). Notably, the choice of solvent significantly influenced the radiochemistry conversions (RCCs), with the RCC of [^18^F]**4** reaching 49% when using the highly polar solvent DMSO, in which Cu(OAc)_2_ is well solubilized (Fig. 2a). A time-RCC study revealed a rapid increase in RCC within the first 5 min, followed by a slight elevation over time, suggesting 10 min as an optimal reaction time considering decay effects (Fig. 2b). Temperature modulation proved to be crucial, with an unfavorable increase causing the reaction system to transition from blue to black. This transformation is assumed to involve the conversion of copper ions to copper oxides, resulting in a decline in RCC. Therefore, the optimal reaction temperature was determined to be 25 °C (Fig. 2c). The optimal amount of precursor was identified as 3 μmol, and the ideal amount of Cu(OAc)_2_ was twice the precursor equivalent dissolved in 200 μL of solvent (Fig. 2d, e). Exploring the effects of different metal ions and phase transfer catalysts on ^18^F-fluorination, it was determined that the most suitable fluorine source was [^18^F]TBAF (Fig. 2f).

Under the optimal labeling conditions, the RCCs of [^18^F]**2** and [^18^F]**3** were 12% and 29%, respectively, as depicted in Fig. 2h. However, [^18^F]**1**, despite having the same di-*tert*-butyl substitute as [^18^F]**4**, exhibited an RCC of 0%. This observation was inferred to be attributed to the exceptionally low boiling point of [^18^F]**1**, leading to its rapid evaporation.

### H_2_O-Resistance of radiofluorination

We explored the water tolerance of the labeling method (Fig. 2g and Additional file 1: Table S20) and observed that the Cu(OAc)_2_-mediated radiofluorination of phosphine oxide substrates can endure water at a level of 300 equivalents concerning the precursor amount, corresponding to 20% of the solvent. This suggests a promising potential for achieving ^18^F-labeling under non-drying conditions.

### A_m_ of Cu(II)-mediated ^18^F-dehydrofluorination versus isotope exchange

Cu(OAc)_2_ (2 equiv.) and precursor **4a** (3 μmol) were individually dissolved in 100 μL DMSO and then sequentially added to the glass vial with dried [^18^F]TBAF (20 mCi). The mixture was incubated at 25 °C for 10 min. Following purification by HPLC, the final product [^18^F]**4** was obtained in a 35% RCY, boasting a radiochemical purity of > 97% and a molar activity of 7.3 GBq/μmol. Similarly, precursor **4** (3 μmol) was dissolved in 200 μL CH_3_CN and added to the glass vial with dried [^18^F]KF/K_222_ (20 mCi). The mixture was incubated at 25 °C for 10 min. Post-purification by Sep-Pak C18 light cartridge, [^18^F]**4** was obtained in an 86% RCY, demonstrating a radiochemical purity of > 97% and a molar activity of 0.5 GBq/μmol. Notably, this work led to a 15-fold improvement in the A_m_ of [^18^F]**4** compared to the isotope exchange method (Fig. 2i).

### Mechanism of Cu(II)-mediated dehydrofluorination

**Scheme 3:**
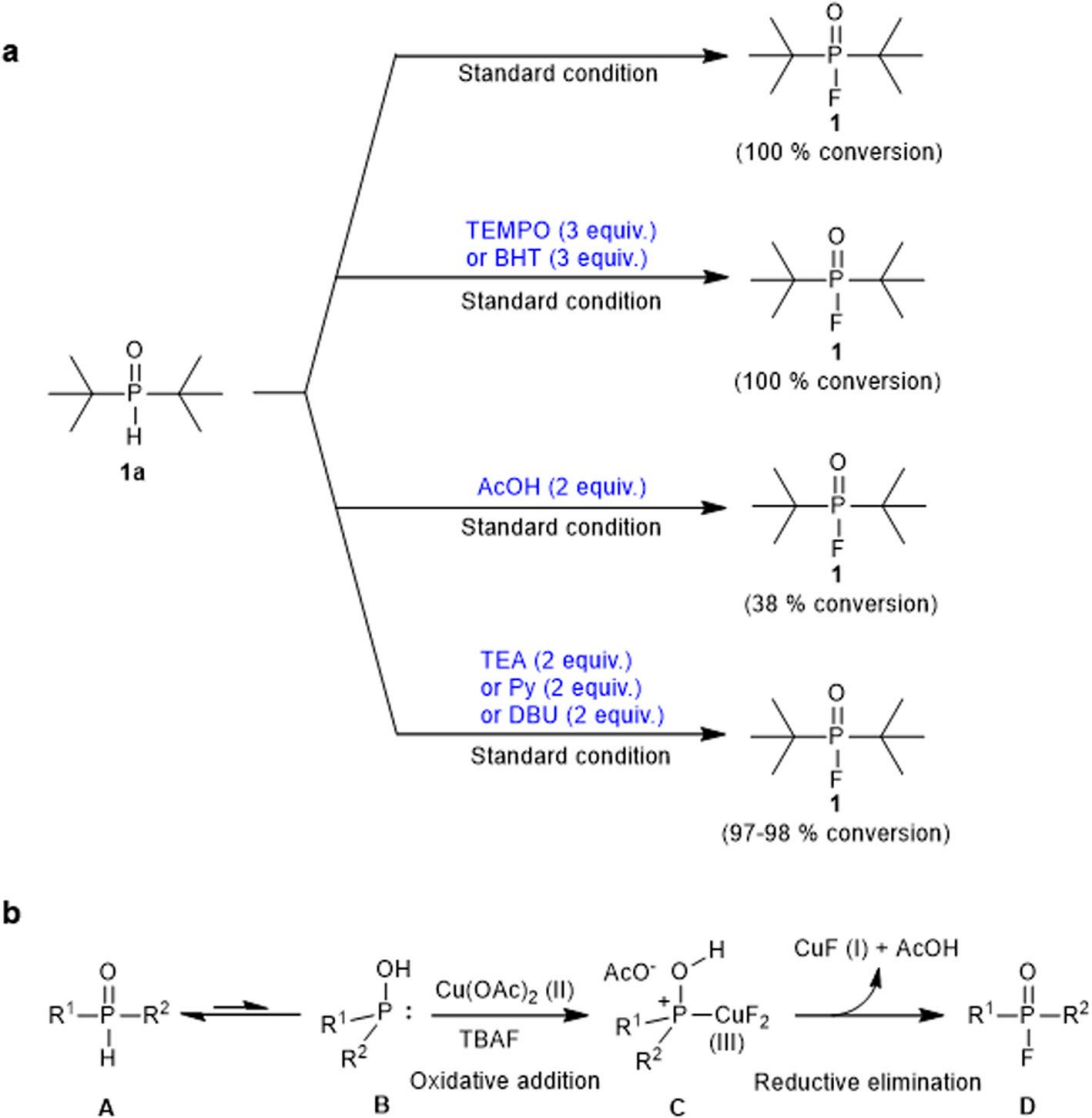
The mechanism of Cu(II)-mediated dehydrofluorination. **a** Experimental mechanistic investigations. Standard condition: **1a** (10 mg), Cu(OAc)_2_ (2 equiv.), acetone (0.5 mL), TBAF (2 equiv.), 1 h, RT. **b** The proposed mechanism for Cu(OAc)_2_-mediated dehydrofluorination on phosphine oxides

In order to gain insight into the mechanism of Cu(OAc)_2_-mediated fluorination, we conducted a preliminary mechanistic study outlined in Scheme 3a. Previous studies have established that copper salts can directly react with Ph_2_P(O)H to generate phosphorus radicals (Ke et al. 2015; Yi et al. 2016; Yang et al. 2015). Thus, we hypothesized that this fluorination mechanism might involve a radical pathway. To test this hypothesis, radical trapping experiments were performed using the commonly employed radical capture agents, 2,2,6,6-tetramethylpiperidine-1-oxyl (TEMPO) and 2,6-di-*tert*-butyl-4-methylphenol (BHT), known for effectively capturing free radicals in chemical reactions (Li et al. 2023; Ye et al. 2023). Surprisingly, the conversion of product **1** remained at 100% when 3.0 equivalents of TEMPO or BHT were added under standard condition. Furthermore, high-resolution mass spectrometry (HRMS) didn’t detect TEMPO- and BHT-coupled products. These results strongly suggest that this transformation may not actually involve the generation of radicals. Additionally, the addition of AcOH (2.0 equiv.) to the reaction under standard condition led to a significant decrease in the conversion of **1** from 100% to 38%. In contrast, the conversion of **1** remained essentially unchanged when 2.0 equivalents of base (TEA, Py, and DBU) were added. These results indicate that the pH of the reaction system may be a crucial factor influencing the reaction. We also speculated that AcOH was generated during the reaction, and the addition of AcOH inhibited the reaction, while the addition of these bases could react with the generated AcOH, promoting the reaction to proceed forward.

Based on the experimental results and literature research (Zhou et al. 2014; Shen et al. 2021), we propose a plausible mechanism for the Cu(OAc)_2_-mediated fluorination reaction, as illustrated in Scheme 3b. Initially, phosphine oxides undergo reciprocal isomerization from the pentavalent phosphorus compound **A** to the less-stable trivalent phosphorus compound **B**, exposing a pair of electrons. Subsequently, the coordination of copper fluoride with electrons on the trivalent phosphorus compound leads to the formation of the oxidation addition intermediate **C**. Finally, the reductive elimination process involves AcO^−^ attacking the H atom of OH and the F atom attacking the P atom, resulting in the formation of the fluorinated products **D**, along with CuF and AcOH.

## Discussion

Considering the established in vivo stable structures of phosphinic fluorides, compounds **1**–**3** were synthesized with a thoughtful consideration of both the site-blocking factor and the conjugation effect. To evaluate their hydrolytic stabilities, assessments were conducted in a mixed solvent containing D_2_O and CD_3_CN using ^19^F NMR (Additional file 1: Figure S2). The site-blocking effect emerged as a crucial factor in stabilizing disubstituted phosphinic fluorides. Compound **1**, characterized by a bis-*tert*-butyl structure with substantial steric hindrance, demonstrated no defluorination even after 9 days of incubation, aligning with findings from previous literature. In contrast, the diphenyl-substituted phosphoryl fluoride structure exhibited a higher susceptibility to hydrolysis. The stable phosphine oxides used in this method is, in fact, one of the reactants involved in the synthesis of phosphinic fluorides (the precursors for isotope exchange), which streamlines the synthesis pathway for the precursor.

The screening process involved the evaluation of various readily available oxidative metal salts, encompassing conventional halides, oxides, inorganic acid-derived metal salts, and organic acid-derived metal salts. While Cu(OAc)_2_ demonstrated the highest fluorination efficiency, it's worth mentioning that the less toxic alternative AgNO_3_ also exhibited satisfactory fluorination efficiency. While Cu(I)-based chelates are commonly used in clinical studies with various radiotracers (Anderson et al. 2009), it is crucial to consider the potential toxicity of Cu(OAc)_2_, even though minute amounts are used in PET imaging radiotracers. In order to ensure the removal of any Cu^2+^ residue, inductively coupled plasma mass spectrometry (ICP-MS) analysis can be conducted after the HPLC purification.

The significant steric hindrance effect was observed to be unfavorable for nucleophilic attack by F^−^, as evidenced by the gradual increase in RCCs as the site resistance decreased. Therefore, achieving a delicate balance between hydrolytic stability and RCC (reactivity towards F^−^), the *tert*-butylphenylphosphorylfluoride structure represents a reasonable compromise.

In the context of labeling hydrophilic substrates such as small molecules, polypeptides, and proteins, considering the water resistance property of the ^18^F-labeling method is essential. Our method exhibits a remarkable tolerance of up to 20% solvent water content, rendering it suitable for the ^18^F-labeling of certain water-soluble biomolecules and precursors that may be highly sensitive or insoluble in organic solvents.

The A_m_ is a critical consideration in the preparation of a receptor-targeting tracer. This method significantly enhances the A_m_ of the activated ester [^18^F]**4** by nearly 15-fold compared to the ^18^F/^19^F-isotope exchange method with the same precursor load and initial activity.

A review of previous studies revealed that the copper-mediated radiofluorination method plays a crucial role in the construction of aromatic C-^18^F bonds, significantly broadening the chemical space of ^18^F-labeling methodology, as well as improving the synthesis of radiopharmaceuticals and advancing PET imaging studies (Wright et al. 2020). In this work, we present a novel Cu(II)-mediated dehydrofluorination of phosphine oxides to construct P-^18^F bonds. This method offers a possibility for ^18^F-labeling of phosphorus-containing biomolecules and will hopefully be applied to the study of C-^18^F bond formation as well as to simplify the process of probe production.

## Conclusion

In summary, this work has disclosed a Cu(OAc)_2_-mediated ^18^F-dehydrofluorination method for phosphine oxides, facilitating the formation of P–F bonds, and proposes a plausible mechanism for this process. In particular, the A_m_ of the activated ester [^18^F]**4** is significantly increased by nearly 15-fold compared to the ^18^F/^19^F-isotope exchange method. Furthermore, the approach exhibits remarkable tolerance of up to 20% aqueous phase, holding promise for the realization of ^18^F-labeled water-soluble biomolecules and drug molecules suitable for positron emission tomography imaging under non-drying conditions. Eventually, a Cu(II)-mediated redox fluorination mechanism was proposed by monitoring the change in conversion rate upon the addition of radical trappers and additives.

## Methods

### General

All the reagents we used in this work were purchased from Energy Chemical Co., Ltd. (China) and J&K Co., Ltd. (China), and were used without further purification. Column chromatography purification was performed on silica gel (54–74 μm, Qingdao Haiyang Chemical Co., Ltd., China). Anhydrous dichloromethane, anhydrous tetrahydrofuran (THF), anhydrous dimethyl sulfoxide (DMSO), anhydrous acetonitrile, anhydrous dimethylformamide (DMF) and anhydrous methol (CH_3_OH) were purchased from Energy Chemical Co., Ltd. (China) and used without further drying.

Proton-1, carbon-13, fluorine-19, and phosphorus-31 nuclear magnetic resonance (^1^H, ^13^C, ^19^F, ^31^P NMR) spectra were recorded on an AS 400 MHz NMR spectrometer (ZhongKeNiuJin, China, ^1^H NMR at 400 MHz, ^13^C NMR at 101 MHz, ^19^F NMR at 376 MHz, ^31^P NMR at 162 MHz). Tetramethylsilane (TMS) was used as an internal standard for ^1^H NMR, and all the chemical shifts were reported as δ values relative to the internal TMS. Chemical shifts for protons were reported in parts per million (ppm) downfield from TMS and were referenced to residual protium in the solvent (^1^H NMR: CDCl_3_ at 7.26 ppm, D_2_O at 4.79 ppm, MeOD at 3.31 ppm, and DMSO-*d*_*6*_ at 2.50 ppm). Chemical shifts for ^13^C signals were referenced to the carbon resonances of the solvent peak (^13^C NMR: CDCl_3_ at 77.16 ppm, MeOD at 49.00 ppm, and DMSO-d_6_ at 39.52 ppm). Multiplicity was defined by s (singlet), d (doublet), t (triplet), and m (multiplet). The coupling constants (*J*) were reported in Hertz (Hz).

HPLC separation was achieved on an Ultimate XB-C_18_ (5 µm, 10 mm × 250 mm) column (Welch, China). HPLC analysis was achieved on a 5 C_18_-MS-II (4.4 µm, 4.6 mm × 250 mm) column (Nacalai Tesque Cosmosil, Japan). Thin layer chromatography (TLC) was performed on TLC Silica gel 60 F254 aluminum sheets (Energy, China), and visualized with short wave UV light (254 nm) or iodine staining. Sep-Pak® light QMA cartridge (Waters, USA) was flushed with 5.0 mL KHCO_3_ solution (0.5 mol L^−1^), air, 10.0 mL water and air before use. Sep-Pak® Plus C18 cartridges (Waters, USA) was flushed with 5.0 mL alcohol, air, 10.0 mL water and air before use. RCCs were obtained by calculating the ratio of the radioactive peak area of a ^18^F-product to the total radioactive peak area. RCCs were determined by radio-TLC and radio-HPLC. Radio-TLC was performed on a Mini-Scan (Eckert & Ziegler, Germany) equipped with a Flow-Count (Bioscan, USA). Radio-HPLC was performed on a Dionex Ulti-Mate 3000 HPLC (Thermo Fisher, USA) equipped with a SPD-20A UV detector (Thermo Fisher, USA) and a Gabi Star γ-radiation detector (Elysia Raytest, Hungary).

### Chemistry

#### Di-*tert*-butylphosphine oxide (1a)

Di-*tert*-butylphosphine chloride (1.8066 g, 10.00 mmol) and 20 mL dichloromethane were added to a dry round bottom flask, which was filled with nitrogen. And then pure water (0.1802 g, 10.00 mmol) was slowly added dropwise under ice bath. When all the pure water had been added, the resulting solution was allowed to warm to room temperature and stirred for 5 h. After the hydrolysis reaction was completed, the mixture was directly rotary evaporated to obtain a white solid compound **1a** (1.5936 g, 98% yield). ^1^H NMR (400 MHz, CDCl_3_): δ = 6.26 (d, *J* = 447.0, 1H), 1.34 (d, *J* = 15.9, 18H). ^13^C NMR (101 MHz, CDCl_3_): δ 27.56, 26.27, 25.00, 23.74. ^31^P NMR (162 MHz, CDCl_3_): δ 71.66. MS (ESI^+^): Calcd for C_8_H_20_OP [M + H]^+^ requires 163.1, found 163.1. (NMR spectra were shown in Additional file 1: Figure S13–S15, and MS spectrum was shown in Additional file 1: Figure S52.)

#### Di-*tert*-butylphosphinic fluoride (1)

Compound **1a** (0.6489 g, 4.00 mmol) was added to a dry round bottom flask with stirred suspension of CuCl_2_ (1.0756, 8.00 mmol) and CsF (1.2152 g, 8.00 mmol) in acetone (30 mL) at room temperature in one shot. The resulting mixture was stirred at room temperature for 12 h. The crude product was purified by silica gel column chromatography (petroleum ether: ethyl acetate = 5: 1) to give the desired product **1** as a white solid (0.2945 g, 41% yield). ^1^H NMR (400 MHz, CDCl_3_): δ = 1.31 (d, *J* = 15.1, 18H). ^13^C NMR (101 MHz, CDCl_3_): δ 26.65, 26.60, 26.55, 25.33. ^31^P NMR (162 MHz, CDCl_3_): δ = 77.52 (d, *J* = 1063.3). ^19^F NMR (376 MHz, CDCl_3_): δ = -101.90 (d, *J* = 1063.4). MS (ESI^+^): Calcd for C_8_H_19_FOP [M + H]^+^ requires 181.1, found 181.1. (NMR spectra were shown in Additional file 1: Figure S16–S19, and MS spectrum was shown in Additional file 1: Figure S53.)

#### *tert*-Butyl(phenyl)phosphine oxide (2a)

*tert*-Butyl(phenyl)phosphine chloride (1.003 g, 10.00 mmol) and 20 mL dichloromethane were added to a dry round bottom flask, which was filled with nitrogen. And then pure water (0.1802 g, 10.00 mmol) was slowly added dropwise under ice bath. When all the pure water had been added, the resulting solution was allowed to warm to room temperature and stirred for 5 h. After the hydrolysis reaction was completed, the mixture was directly rotary evaporated to obtain a white solid compound 2a (0.8864 g, 97% yield). ^1^H NMR (400 MHz, CDCl_3_): δ = 7.70–7.48 (m, 5H), 1.16 (dd, *J* = 17.0, 1.3, 9H). ^13^C NMR (101 MHz, CDCl_3_): δ = 132.88 (d, *J* = 2.8), 131.09 (d, *J* = 10.3), 128.70 (d, *J* = 11.9), 31.97 (d, *J* = 68.8), 23.42 (d, *J* = 2.2). ^31^P NMR (162 MHz, CDCl_3_): δ 49.24. MS (ESI^+^): Calcd for C_10_H_16_OP [M + H]^+^ requires 183.1, found 183.1. (NMR spectra were shown in Additional file 1: Figure S20–S22, and MS spectrum was shown in Additional file 1: Figure S54.)

#### *tert*-Butyl(phenyl)phosphinic fluoride (2)

Compound **2a** (0.7288 g, 4.00 mmol) was added to a dry round bottom flask with stirred suspension of CuCl_2_ (1.0756, 8.00 mmol) and CsF (1.2152 g, 8.00 mmol) in acetone (30 mL) at room temperature in one shot. The resulting mixture was stirred at room temperature for 12 h. The crude product was purified by silica gel column chromatography (petroleum ether: ethyl acetate = 5: 1) to give the desired product **2** as a white solid (0.4478 g, 56% yield). ^1^H NMR (400 MHz, CDCl_3_): δ = 7.89–7.44 (m, 5H), 1.23 (d, *J* = 16.7, 9H). ^13^C NMR (101 MHz, CDCl_3_): δ = 133.23 (d, *J* = 2.8), 132.57 (dd, *J* = 9.8, 2.8), 128.58 (d, *J* = 12.5), 33.09 (dd, *J* = 95.9, 15.2), 23.94. ^31^P NMR (162 MHz, CDCl_3_): δ = 61.51 (d, *J* = 1051.8). ^19^F NMR (376 MHz, CDCl_3_): δ = − 94.68 (d, *J* = 1052.0). MS (ESI^+^): Calcd for C_10_H_15_FOP [M + H]^+^ requires 201.1, found 201.1. (NMR spectra were shown in Additional file 1: Figure S23–S26, and MS spectrum was shown in Additional file 1: Figure S55.)

#### Diphenylphosphinic fluoride (3)

Diphenylphosphine oxide (0.4044 g, 2.00 mmol) and Selectfkuor (1-chloromethyl-4-fluoro-1,4-diazoniabicyclo[2.2.2]octane bis(tetrafluoroborate), 0.7794 g, 2.20 mmol)) were added to a dry round bottom flask containing 20 mL acetonitrile. The resulting mixture was stirred at room temperature for 12 h. The crude product was purified by silica gel column chromatography (petroleum ether: ethyl acetate = 3: 1) to give the desired product **3** as a light-yellow oil (0.3545 g, 81% yield). ^1^H NMR (400 MHz, CDCl_3_): δ = 7.83 (dd, *J* = 13.2, 7.6, 4H), 7.62 (t, *J* = 7.5, 2H), 7.52 (td, *J* = 7.6, 3.7, 4H). ^13^C NMR (101 MHz, CDCl_3_): δ = 133.44 (d, *J* = 3.2), 131.48 (dd, *J* = 11.4, 2.0), 128.89 (d, *J* = 14.0). ^31^P NMR (162 MHz, CDCl_3_) δ = 40.93 (d, *J* = 1020.3). ^19^F NMR (376 MHz, CDCl_3_) δ = − 75.08 (d, *J* = 1019.8). MS (ESI^+^): Calcd for C_12_H_11_FOP [M + H]^+^ requires 221.1, found 221.1. (NMR spectra were shown in Additional file 1: Figure S27–S30, and MS spectrum was shown in Additional file 1: Figure S56.)

#### Benzyl 2-bromo-2-methylpropanoate (4d)

According to a modified literature procedure (Sun et al. 2019): A 250 mL round bottom flask was degassed and filled with the mixture of benzyl alcohol (10.81 g, 100.00 mmol), triethylamine (TEA, 10.12 g, 100.00 mmol) in 100 mL CH_2_Cl_2_. Next, 2-bromo-2-methylpropanoyl bromide (22.99 g, 100 mmol) was added dropwise into the mixture in an ice-water bath at 0 °C for 1 h. Then, allow the reaction mixture warm to room temperature and was stirred for another 12 h. After the reaction was completed, the resulting solution was washed with water (100 mL × 3) and dried on magnesium sulfate. The filtrate was concentrated *in vacuo* and purified by a short column chromatography on silica gel using hexanes and ethyl acetate (50:1) as elute to give the corresponding colorless oil liquid **4d** (73% yield). ^1^H NMR (400 MHz, CDCl_3_): δ = 7.41–7.31 (m, 5H), 5.22 (d, *J* = 1.2, 2H), 1.96 (d, *J* = 1.2, 6H). ^13^C NMR (101 MHz, CDCl_3_): δ = 171.51, 135.49, 129.67–126.89 (m), 67.60, 55.72, 30.83. MS (ESI^+^): Calcd for C_11_H_13_BrO_2_K [M + K]^+^ requires 295.0, found 295.2. (NMR spectra were shown in Additional file 1: Figure S31–S32, and MS spectrum was shown in Additional file 1: Figure S57.)

#### Benzyl 2-(*tert*-butylhydrophosphoryl)-2-methylpropanoate (4c)

Route 1: A 250 mL three-necked round bottom flask containing zinc powder (719.18 mg, 11.00 mmol) was degassed and flushed with dry nitrogen and charged with 30 mL THF. A solution of iodine in 10 mL dry THF was added in the flask with vigorous stir. Next, a solution of benzyl 2-bromo-2-methylpropanoate (**4d**, 2.57 g, 10.00 mmol) in 40 mL THF was placed in dropping funnel and 5 mL of the solution was added to trigger the formation of Grignard reagent. After the mixture turning murky, residual **4d** was added dropwise into the mixture for another 4 h.

After zinc powder was nearly consumed, *tert*-butyldichlorophosphane (TBDCP, 1.59 g, 10.00 mmol) in 40 mL THF was added dropwise into the flask under nitrogen in an ice-water bath at 0 °C for 1 h. Then, allow the reaction mixture warm to room temperature and was stirred for another 12 h. The reaction was then quenched with 1 mol·L^−1^ HCl (20 mL) and extracted with ethyl acetate (80 × 3 mL). The organic layers were combined and concentrated and washed with saturated brine (50 × 3 mL). The organic layer was then dried on magnesium sulfate, filtered, and concentrated again *in vacuo*. The concentrate was purified by a short column chromatography on silica gel using dichloromethane and methanol (80:1) as elute to give the corresponding white solid (37% yield).

Route 2: A 100 mL three-necked round bottom flask containing Magnesium (Mg) chips (160.41 mg, 6.60 mmol) was degassed and flushed with dry nitrogen and charged with 20 mL THF. After heating the mixture at boiling point, a solution of benzyl 2-bromo-2-methylpropanoate (**4d**, 1.54 g, 6.00 mmol) in 20 mL THF was placed in dropping funnel and 4 mL of the solution was added to trigger the formation of Grignard reagent. After naturally cooling the flask to room temperature, the residual **4d** was added dropwise into the mixture for another 4 h.

Next, ethyl *tert*-butylphosphinate (**4e**, 900.95 mg, 6.00 mmol) in 20 mL THF was added dropwise into the flask under nitrogen in an ice-water bath at 0 °C for 1 h. Then, allow the reaction mixture warm to room temperature and was stirred for another 12 h. The reaction was then quenched with 1 mol·L^−1^ HCl (10 mL) and extracted with ethyl acetate (60 × 3 mL). The organic layers were combined and washed with saturated brine (50 × 2 mL). The organic layer was then dried on magnesium sulfate, filtered, and concentrated again *in vacuo*. The concentrate was purified by a short column chromatography on silica gel using dichloromethane and methanol (80:1) as elute to give the corresponding white solid (8% yield). ^1^H NMR (400 MHz, CDCl_3_): δ = 7.39 (dq, *J* = 6.9, 3.9, 2.6, 5H), 6.56 (d, *J* = 463.7, 1H), 5.27–5.11 (m, 2H), 1.67 (d, *J* = 14.4, 3H), 1.54 (d, *J* = 12.4, 3H), 1.19 (d, *J* = 16.2, 9H). ^13^C NMR (101 MHz, CDCl_3_): δ = 173.10, 135.00, 129.67–126.71 (m), 67.32, 44.00 (d, *J* = 49.8), 34.06 (d, *J* = 61.4), 24.82, 24.24. ^31^P NMR (162 MHz, CDCl_3_): δ = 58.53. MS (ESI^+^): Calcd for C_15_H_23_O_3_PNa [M + Na]^+^ requires 305.1, found 305.1. (NMR spectra were shown in Additional file 1: Figure S33–S35, and MS spectrum was shown in Additional file 1: Figure S58.)

#### 2-(*tert*-Butylhydrophosphoryl)-2-methylpropanoic acid (4b)

Route 1: According to a literature procedure (Hong et al. 2019): A 25 mL three-necked round bottom flask containing 10% Pd/C (957.8 mg, 9 mmol, 10 wt%) was degassed and charged with 8 mL MeOH, followed by a solution of benzyl 2-(*tert*-butylhydrophosphoryl)-2-methylpropanoate (**4c**, 846.9 mg, 3.00 mmol) in 3 mL MeOH. Hydrogen was then bubbled into the stirred reaction mixture continuously. After 12 h, the solution was filtered and concentrated *in vacuo*. The concentrate was purified by a short column chromatography on silica gel using dichloromethane and methanol (20:1) as elute to give the corresponding white solid (75% yield).

Route 2: A 100 mL round bottom flask was filled with ethyl 2-(*tert*-butylhydrophosphoryl)-2-methylpropanoate (**4f**, 1.10 g, 5.00 mmol), then a solution of sodium hydroxide (NaOH, 599.9 mg, 15 mmol) in 40 mL water was added to the reaction flask. After stirring for 5 h, the starting material was completely consumed (by TLC). Next, the mixture was acidified to pH = 2 with 1 mol·L^−1^ HCl and extracted with ethyl acetate (40 × 3 mL). The organic layer was then dried with magnesium sulfate, filtered, and concentrated *in vacuo* to give a white solid of pure compound **4b** (91% yield). ^1^H NMR (400 MHz, CDCl_3_): δ = 6.56 (d, *J* = 471.2, 1H), 1.59 (d, *J* = 14.9, 3H), 1.49 (d, *J* = 13.3, 3H), 1.25 (d, *J* = 16.3, 9H). ^13^C NMR (101 MHz, CDCl_3_): δ = 174.86, 43.76 (d, *J* = 50.8), 34.06 (d, *J* = 61.1), 24.94, 23.09. ^31^P NMR (162 MHz, CDCl_3_): δ = 61.17. MS (ESI^+^): Calcd for C_8_H_18_O_3_P [M + H]^+^ requires 193.1, found 193.1. (NMR spectra were shown in Additional file 1: Figure S36–S38, and MS spectrum was shown in Additional file 1: Figure S59).

#### 2,3,5,6-Tetrafluorophenyl 2-(*tert*-butylhydrophosphoryl)-2-methylpropanoate (4a)

According to a modified literature procedure (Hong et al. 2019): A 100 mL three-necked round bottom flask containing 2-(*tert*-butylhydrophosphoryl)-2-methylpropanoic acid (**4b**, 576.58 mg, 3.00 mmol) was degassed and flushed with dry nitrogen and charged with 20 mL dry CH_2_Cl_2_. Next, a solution of 4-dimethylaminopyridine (DMAP, catalytic amount) and dicyclohexylcarbodiimide (DCC, 742.80 mg, 3.60 mmol) in 20 mL dry CH_2_Cl_2_ was added to the flask in an ice-water bath. The reaction mixture was stirred for 20 min at 0 °C, then a solution of 2,3,5,6-tetrafluorophenol (597.87 mg, 3.60 mmol) in 10 mL dry CH_2_Cl_2_ was added dropwise. Then, allow the reaction mixture warm to room temperature and was stirred overnight. The mixture was later concentrated *in vacuo* and redissolve in ethyl acetate (30 mL), filtered, and reconcentrated and purified by a short column chromatography on silica gel using dichloromethane and ethyl acetate (2:1) as elute to give the corresponding white solid (61% yield). ^1^H NMR (400 MHz, CDCl_3_): δ = 7.04 (p, *J* = 8.5, 1H), 6.72 (d, *J* = 464.2, 1H), 1.82 (d, *J* = 13.5, 3H), 1.65 (d, *J* = 11.8, 3H), 1.29 (dd, *J* = 16.7, 1.4, 9H). ^13^C NMR (101 MHz, CDCl_3_): δ = 170.21, 151.31–126.91 (m), 103.72, 44.51 (d, *J* = 48.5), 34.41 (d, *J* = 62.4), 24.82. ^31^P NMR (162 MHz, CDCl_3_): δ = 56.04. MS (ESI^+^): Calcd for C_14_H_17_F_4_O_3_PNa [M + Na]^+^ requires 363.1, found 362.9. (NMR spectra were shown in Additional file 1: Figure S39–S41, and MS spectrum was shown in Additional file 1: Figure S60.)

#### Ethyl *tert*-butylphosphinate (4e)

According to a modified literature procedure (Liu et al. 2017): A 50 mL round bottom flask was filled with *tert*-butyldichlorophosphane (TBDCP, 1.59 g, 10.00 mmol) and charged with 10 mL THF. Anhydrous ethanol (EtOH, 20 mL) was then added at room temperature and the reaction mixture was stirred for another 12 h. After TBDCP was completely consumed (by ^31^P NMR), the mixture was concentrated *in vacuo* to give a colorless oil of pure compound **4e** (99% yield). ^1^H NMR (400 MHz, CDCl_3_): δ = 6.71 (d, *J* = 516.7, 1H), 4.40–3.89 (m, 2H), 1.57–1.25 (m, 3H), 1.12 (d, *J* = 17.8, 9H). ^13^C NMR (101 MHz, CDCl_3_): δ = 58.06, 30.81 (d, *J* = 96.3), 22.58, 18.09. ^31^P NMR (162 MHz, CDCl_3_): δ = 49.43. MS (ESI^+^): Calcd for C_6_H_15_O_2_PNa [M + Na]^+^ requires 173.1, found 173.0. (NMR spectra were shown in Additional file 1: Figure S42–S44, and MS spectrum was shown in Additional file 1: Figure S61.)

#### Ethyl 2-(*tert*-butylhydrophosphoryl)-2-methylpropanoate (4f)

A 250 mL three-necked round bottom flask containing zinc powder (1.44 g, 22.00 mmol) was degassed and flushed with dry nitrogen and charged with 50 mL THF. A solution of iodine in 10 mL dry THF was added in the flask with vigorous stir. Next, a solution of ethyl 2-bromo-2-methylpropanoate (EBMPA, 3.90 g, 20.00 mmol) in 40 mL THF was placed in dropping funnel and 5 mL of the solution was added to trigger the formation of Grignard reagent. After the mixture turning loose and murky, residual 2-bromo-2-methylpropanoate was added dropwise into the mixture for another 4 h.

After zinc powder was nearly consumed, *tert*-butyldichlorophosphane (TBDCP, 3.18 g, 20.00 mmol) in 50 mL THF was added dropwise into the flask under nitrogen in an ice-water bath at 0 °C for 1 h. Then, allow the reaction mixture warm to room temperature and was stirred for another 12 h. The reaction was then quenched with 1 mol·L^−1^ HCl (30 mL) and extracted with ethyl acetate (80 × 3 mL). The organic layers were combined and concentrated and washed with saturated brine (50 × 3 mL). The organic layer was then dried on magnesium sulfate, filtered, and concentrated again *in vacuo*. The concentrate was purified by a short column chromatography on silica gel using dichloromethane and methanol (80:1) as elute to give the corresponding white solid (59% yield). ^1^H NMR (400 MHz, CDCl_3_): δ = 6.56 (d, *J* = 463.6, 1H), 4.32–4.10 (m, 2H), 1.64 (d, *J* = 14.5, 3H), 1.52 (d, *J* = 12.4, 3H), 1.33 (t, *J* = 7.1, 3H), 1.24 (d, *J* = 16.1, 9H). ^13^C NMR (101 MHz, CDCl_3_): δ = 173.28, 61.55, 43.86 (d, *J* = 50.1), 34.07 (d, *J* = 61.4), 24.89, 24.26, 13.97. ^31^P NMR (162 MHz, CDCl_3_): δ = 58.93. MS (ESI^+^): Calcd for C_10_H_21_O_3_PNa [M + Na]^+^ requires 243.1, found 243.1. (NMR spectra were shown in Additional file 1: Figure S45–S47, and MS spectrum was shown in Additional file 1: Figure S62).

#### 2,3,5,6-Tetrafluorophenyl 2-(*tert*-butylfluorophosphoryl)-2-methylpropanoate (4)

Compound **4a** (0.1021 g, 0.30 mmol), CuCl_2_ (0.0807 g, 0.60 mmol) and CsF (0.0807 g, 0.60 mmol) were added to a dry round battom flask with acetone (25 mL) as the reaction solvent. The resulting mixture was stirred at room temperature for 12 h. The crude product was purified by silica gel column chromatography (petroleum ether: ethyl acetate = 6: 1) to obtain the desired product **4** as a white solid (0.0335 g, yield: 31%). ^1^H NMR (400 MHz, CDCl_3_): δ = 7.03 (tt, *J* = 9.8, 7.1, 1H), 1.87–1.71 (m, 6H), 1.36 (d, *J* = 17.1, 9H). ^13^C NMR (101 MHz, CDCl_3_): δ = 170.21, 149.56–123.10 (m), 103.73, 44.49 (d, *J* = 48.5), 34.41 (d, *J* = 62.5), 24.81, 24.62. ^31^P NMR (162 MHz, CDCl_3_): δ = 66.73 (d, *J* = 1090.0). ^19^F NMR (376 MHz, CDCl_3_): δ = − 92.90 (d, *J* = 1089.8), − 135.15 to − 141.24 (m), − 148.8 to − 155.00 (m). MS (ESI^+^): Calcd for C_14_H_17_F_5_O_3_P [M + H]^+^ requires 359.1, found 359.2. (NMR spectra were shown in Additional file 1: Figure S48–S51, and MS spectrum was shown in Additional file 1: Figure S63.)

### Stability of phosphine oxide 1a in different pH aqueous solutions

Compound **1a** (10 mg in 0.2 mL deuterated acetonitrile) was incubated with pH 1.00 (hydrochloric acid, 0.1 mol·L^−1^, 0.4 mL), pH 4.00 (potassium acid phthalate, 0.05 mol·L^−1^, 0.4 mL), pH 7.00 (ultra-pure water, 0.4 mL), pH 10.00 (borax/sodium hydroxide, 0.0125 mol·L^−1^, 0.4 mL), pH 12.00 (sodium hydroxide, 0.01 mol·L^−1^, 0.4 mL) at room temperature in 24 h, respectively. The percentage of remaining compound **1a** was monitored by ^31^P NMR in the following 24 h from the mixed solution (Additional file 1: Figure S1).

### Screening of metal salts and optimization of Cu(OAc)_2_, CuCl_2_ and AgNO_3_-mediated fluorination reaction conditions

Compound **1a** (0.0100 g, 0.0617 mmol), metal salt (2 equiv.) and were added to a 2 mL centrifuge tube with solvent (0.5–0.6 mL) as the reaction solvent. Oxidative transition metal salts, such as, Cu(II), Cu(I), Ag(I), Pd(II), Fe(III), Fe(II), Ni(II), Mn(II), Zn(II), and Pt(II) salts were tried. Each reactant was sonicated to dissolve as much as possible and then transferred to a magnetic stirrer. Then, fluorine source (2 equiv.) was added to the system, and the reaction was carried out at the corresponding temperature for 12 h. The reaction was quenched by adding saturated K_2_CO_3_ solution to the reaction solution and centrifuged, and the supernatant was aspirated for ^31^P NMR analysis (Additional file 1: Table S1–S7 and Figure S3).

### Mechanism study

Compound **1a** (0.0100 g, 0.0617 mmol), Cu(OAc)_2_ (0.1233 mmol), TBAF (1 mol·L^−1^ in THF, 0.1233 mL, 0.1233 mmol), and additives (TEMPO, BHT, AcOH, TEA, Py or DBU, 2–3 equiv.), were added to a 2 mL tube with acetone (0.5 mL) as solvent and transferred to a magnetic stirrer. Then, TBAF (1 mol·L^−1^ in THF, 0.1233 mL, 0.1233 mmol) was added to the mixture and stirred at room temperature for 12 h. When the reaction was finished, the resulting mixture was extracted with saturated potassium carbonate solution, and the supernatant was collected for ^31^P NMR analysis. The conversion of the compound **1** was shown in Additional file 1: Table S11.

### H_2_O-Resistance of fluorination

Compound **1a** (0.0100 g, 0.0617 mmol), Cu(OAc)_2_ (0.0246 g, 0.1233 mmol), pure water according to the corresponding stoichiometric ratio and KF (0.0072 g, 0.1233 mmol) were sequentially added to a tube with DMSO (0.4 mL) as reaction solvent (Additional file 1: Scheme S2). The resulting mixture was stirred at room temperature. The reaction was quenched by adding saturated K_2_CO_3_ solution to the reaction solution and centrifuged, and the supernatant was aspirated for ^31^P NMR analysis (Additional file 1: Table S12, S13, and Figure S4).

### Radiochemistry

#### The preparation of dried [^18^F]KF

The [^18^F]F^−^ aqueous was taken in a clean glass vial and KOAc solution (KOAc: 0.5 mg, 5.0 μmol; 200 μL H_2_O) was added to disperse [^18^F]F^−^ uniformly at the bottom of the vial. The solution was azeotropically dried for three times (300 μL anhydrous CH_3_CN × 3) at 100 °C with nitrogen flow. Then the reaction vial was capped to obtain dried [^18^F]KF for radiolabeling.

#### The preparation of dried [^18^F]KF/K_222_

The [^18^F]F^−^ aqueous was taken in a clean glass vial and K_222_/KOAc solution ((K_222_: 3.8 mg, 10.0 μmol; KOAc: 0.5 mg, 5.0 μmol; 400 μL CH_3_CN + 100 μL H_2_O) was added to disperse [^18^F]F^−^ uniformly at the bottom of the vial. The solution was azeotropically dried for three times (300 μL anhydrous CH_3_CN × 3) at 100 °C with nitrogen flow. Then the reaction vial was capped to obtain dried [^18^F]KF/K_222_ for radiolabeling.

#### The preparation of dried [^18^F]KF/18-crown-6

The [^18^F]F^−^ aqueous was taken in a clean glass vial and 18-crown-6/KOAc solution (18-crown-6: 2.8 mg, 10.0 μmol; KOAc: 0.5 mg, 5.0 μmol; 400 μL CH_3_CN + 100 μL H_2_O) was added to disperse [^18^F]F^−^ uniformly at the bottom of the vial. The solution was azeotropically dried for three times (300 μL anhydrous CH_3_CN × 3) at 100 °C with nitrogen flow. Then the reaction vial was capped to obtain dried [^18^F]KF/18-crown-6 ([^18^F]KF/18-crown-6) for radiolabeling.

#### The preparation of dried [^18^F]TBAF

The [^18^F]F^−^ aqueous was taken in a clean glass vial and Bu_4_NOAc solution (TBAA: 3.2 mg, 10.0 μmol; 500 μL CH_3_CN) was added to disperse [^18^F]F^−^ uniformly at the bottom of the vial. The solution was azeotropically dried for three times (300 μL anhydrous CH_3_CN × 3) at 100 °C with nitrogen flow. Then the reaction vial was capped to obtain dried [^18^F]TBAF for radiolabeling.

#### The preparation of dried [^18^F]CsF

The [^18^F]F^−^ aqueous was taken in a clean glass vial and CsOAc solution (CsOAc: 1.0 mg, 5.0 μmol; 200 μL H_2_O) was added to disperse [^18^F]F^−^ uniformly at the bottom of the vial. The solution was azeotropically dried for three times (300 μL anhydrous CH_3_CN × 3) at 100 °C with nitrogen flow. Then the reaction vial was capped to obtain dried [^18^F]CsF for radiolabeling.

#### ^18^F-Dehydrofluorination of phosphine oxides ([^18^F]1–[^18^F]4)

Cu(OAc)_2_ (2 equiv.) and precursor **1a**–**4a** (3 μmol) were dissolved in 100 μL DMSO respectively and sequentially added to the glass vial with dried [^18^F]TBAF. The mixture was incubated at 25 °C for 10 min. RCCs and the corresponding radio-TLC traces were shown in Additional file 1: Table S14–S19, S22–S24, Figure S5–S6 and S8–S12.

#### H_2_O-Resistance of radiofluorination

Precursor **4a** (3 μmol) and Cu(OAc)_2_ (2 equiv.) were dissolved in 100 μL DMSO respectively. Corresponding proportions of pure water, Cu(OAc)_2_, and **4a** were sequentially added to the glass vial with dried [^18^F]TBAF. The mixture was incubated at 25 °C for 10 min. RCCs were shown in Additional file 1: Table S20.

#### A_m_ of [^18^F]4

Solutions of **4** at graded concentrations were prepared and analyzed by an analytical HPLC shown in Additional file 1: Table S21 (phase A: CH3CN; phase B: ultrapure water; isocratic elution at 40% phase A and 60% phase B. Flow rate: 1.0 mL·min^−1^, 20 min, UV = 254 nm.). The UV absorption peak areas at different amounts of **4** was measured, and the relationship between the areas of the absorption peaks and the amounts of the substance was obtained by linear analysis in Additional file 1: Figure S7.

The A_m_ of [^18^F]**4** was calculated by dividing the radioactivity of [^18^F]**4** at end of synthesis by the molar amount of **4** measured by HPLC–UV [nmol of **4**] as interpreted from the UV-standard curve.

### Supplementary Information

**Additional file 1**. Relationship between RCCs and water amount.

## Data Availability

All data are available in the main text or the supplementary material.

## Abbreviations

AcOH: Acetic acid
BHT: Butylated hydroxytoluene
DBU: 1,8-Diazabicyclo[5,4,0]undec-7-ene
DCC: Dicyclohexylcarbodiimide
DCDMH: 1,3-Dichloro-5,5-dimethylhydantoin
DDQ: 2,3-Dichloro-5,6-dicyano-4-benzoquinone
DMAP: 4-Dimethylaminopyridine
DMF: Dimethylformamide
DMSO: Dimethyl sulfoxide
EBMPA: Ethyl 2-bromo-2-methylpropanoate
^18^F: Fluorine-18
HCl: Hydrochloric acid
HPLC: High-performance liquid chromatography
HRMS: High-resolution mass spectrometry
ICP-MS: Inductively coupled plasma mass spectrometry
K_222_: 4,7,13,16,21,24-Hexaoxa-1,10-diazabicyclo[8.8.8]hexacosane
MS: Mass spectrometer
A_m_: Molar activity
NMR: Nuclear magnetic resonance
PET: Positron emission tomography
Py: Pyridine
RCC: Radiochemical conversion
RCY: Radiochemical yield
TBAA: *tert*-Butyl acetoacetate
TBAF: Tetrabutylammonium fluoride
TBDCP: *tert*-Butyldichlorophosphane
TCA: Trichloroacetonitrile
TCICA: 1,3,5-Trichloro-1,3,5-triazinane-2,4,6-trione
TEA: Triethylamine
TEMPO: 2,2,6,6-Tetramethylpiperidinooxy
TFP: 2,3,5,6-Tetrafluorophenol
THF: Tetrahydrofuran
TLC: Thin-layer chromatography
UV: Ultraviolet
