# Supplementary material for: Cu(II)-Mediated direct 18F-dehydrofluorination of phosphine oxides in high molar activity
Source: EJNMMI Radiopharm Chem. 2024 Jan 6;9:4. doi: 10.1186/s41181-023-00234-y (PMC10771395; doi:10.1186/s41181-023-00234-y)
Supplement: Supplementary file 1 — Additional file 1. Relationship between RCCs and water amount. [file 41181_2023_234_MOESM1_ESM.pdf]

Supplementary Material for

Cu(II)-Mediated direct ^{18}F-dehydrofluorination of phosphine oxides in high molar activity

Xiaoqun Tang[1,2], Shengji Lv[1,2], Zhaobiao Mou[1,2], Xia Liu[1,2], Zijing Li[1,2,*]

[1]State Key Laboratory of Vaccines for Infectious Diseases, Center for Molecular Imaging and Translational Medicine, Xiang An Biomedicine Laboratory, School of Public Health, Xiamen University, Xiamen, Fujian 361102, China.

[2]State Key Laboratory of Molecular Vaccinology and Molecular Diagnostics, National Innovation Platform for Industry-Education Intergration in Vaccine Research, Xiamen University, Xiamen, Fujian 361102, China.

Correspondence and requests for materials should be addressed to Z. L. (zijing.li@xmu.edu.cn).

Content

1 Stability of phosphine oxides and phosphinic fluorides S4

1.1 Stability of phosphine oxide 1a in different pH aqueous solutions S4

1.2 Stability of phosphinic fluoride 1 .... S6

2 Metal salt-mediated fluorination of phosphine oxides S8

2.1 Screening of metal salts S8

2.1.1 General procedure S8

2.1.2 Screening of different Cu family salts S8

2.1.3 Screening of different Zn salts S11

2.1.4 Screening of different transition metal salts S11

2.1.5 Screening of different Pd and Pt metal salts S12

2.2 Optimization of $Cu(OAc)_2$, $CuCl_2$ and $AgNO_3$-mediated fluorination reaction conditions S14

2.2.1 Optimization of $Cu(OAc)_2$-mediated fluorination reaction conditions S14

2.2.2 Optimization of $CuCl_2$ -mediated fluorination reaction conditions S15

2.2.3 Optimization of $AgNO_3$-mediated fluorination reaction condition S16

2.2.4 Comparison of $Cu(OAc)_2$, $CuCl_2$ and $AgNO_3$-mediated fluorination reaction conversions with time S17

2.3 Characterization spectrum of ^{31}P NMR S17

2.4 Mechanism study of $Cu(OAc)_2$-mediated dehydrofluorination on phosphine oxides S24

2.5 Effects of water content on the efficiency of copper catalytic fluorination S24

2.5.1 General reaction route S24

2.5.2 General procedure for stoichiometric water addition S24

2.5.3 General procedure for volumetric water addition S25

2.5.4 ^{31}P NMR analysis of substrate conversion on water addition study S26

3 Radiochemistry S27

3.1 $[^{18}F]F^-$ source S27

3.2 General procedure for ^{18}F-fluorination S27

3.3 Optimization of ^{18}F-labeling conditions S27

3.3.1 Relationship between RCCs and different solvents S27

3.3.2 Relationship between RCCs and reaction time S28

3.3.3 Relationship between RCCs and precursor amount S29

3.3.4 Relationship between RCCs and reaction temperature S30

3.3.5 Relationship between RCCs and equivalent of $Cu(OAc)_2$ S31

3.3.6 Relationship between RCCs and ^{18}F-fluorine source S31

3.3.7 Relationship between RCCs and water amount S32

3.3.8 Characterization spectra by radio-TLC and radio-HPLC S33

3.4 UV standard curve of 4 .... S39

3.5 ^{18}F-Labeling of [^{18}F]1-[^{18}F]3 .... S39

3.5.1 ^{18}F-Labeling of [^{18}F]1 .... S39

3.5.2 ^{18}F-Labeling of [^{18}F]2 .... S40

3.5.3 ^{18}F-Labeling of [^{18}F]3 .... S41

4 Appendix (^{1}H, ^{13}C, ^{19}F, ^{31}P NMR and MS spectra) S42

1 Stability of phosphine oxides and phosphinic fluorides

1.1 Stability of phosphine oxide 1a in different pH aqueous solutions

Compound **1a** (10 mg in 0.2 mL deuterated acetonitrile) was incubated with pH 1 (hydrochloric acid, 0.1 mol·L^{-1}, 0.4 mL), pH 4 (potassium acid phthalate, 0.05 mol·L^{-1}, 0.4 mL), pH 7 (ultra-pure water, 0.4 mL), pH 10 (borax/sodium hydroxide, 0.0125 mol·L^{-1}, 0.4 mL), pH 12 (sodium hydroxide, 0.01 mol·L^{-1}, 0.4 mL) at room temperature in 24 h, respectively. The percentage of remaining compound **1a** was monitored by ^{31}P NMR in the following 24 h from the mixed solution.

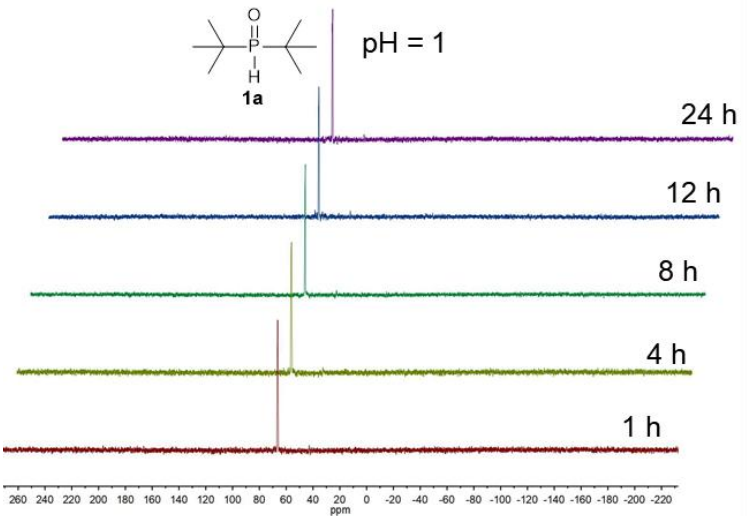

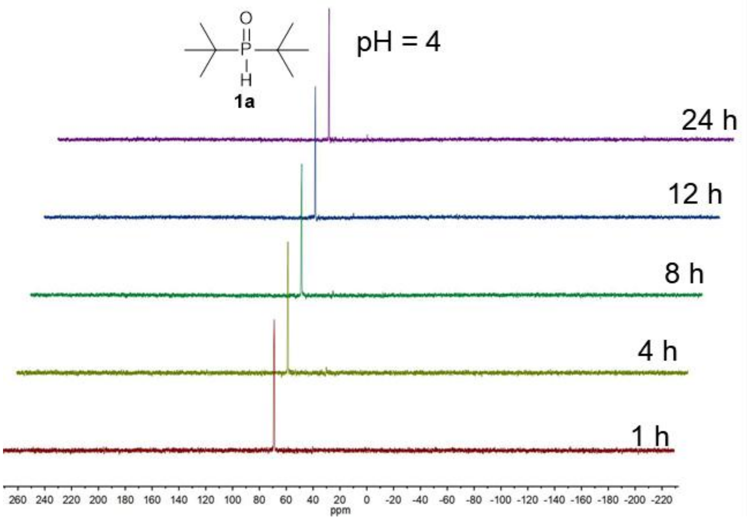

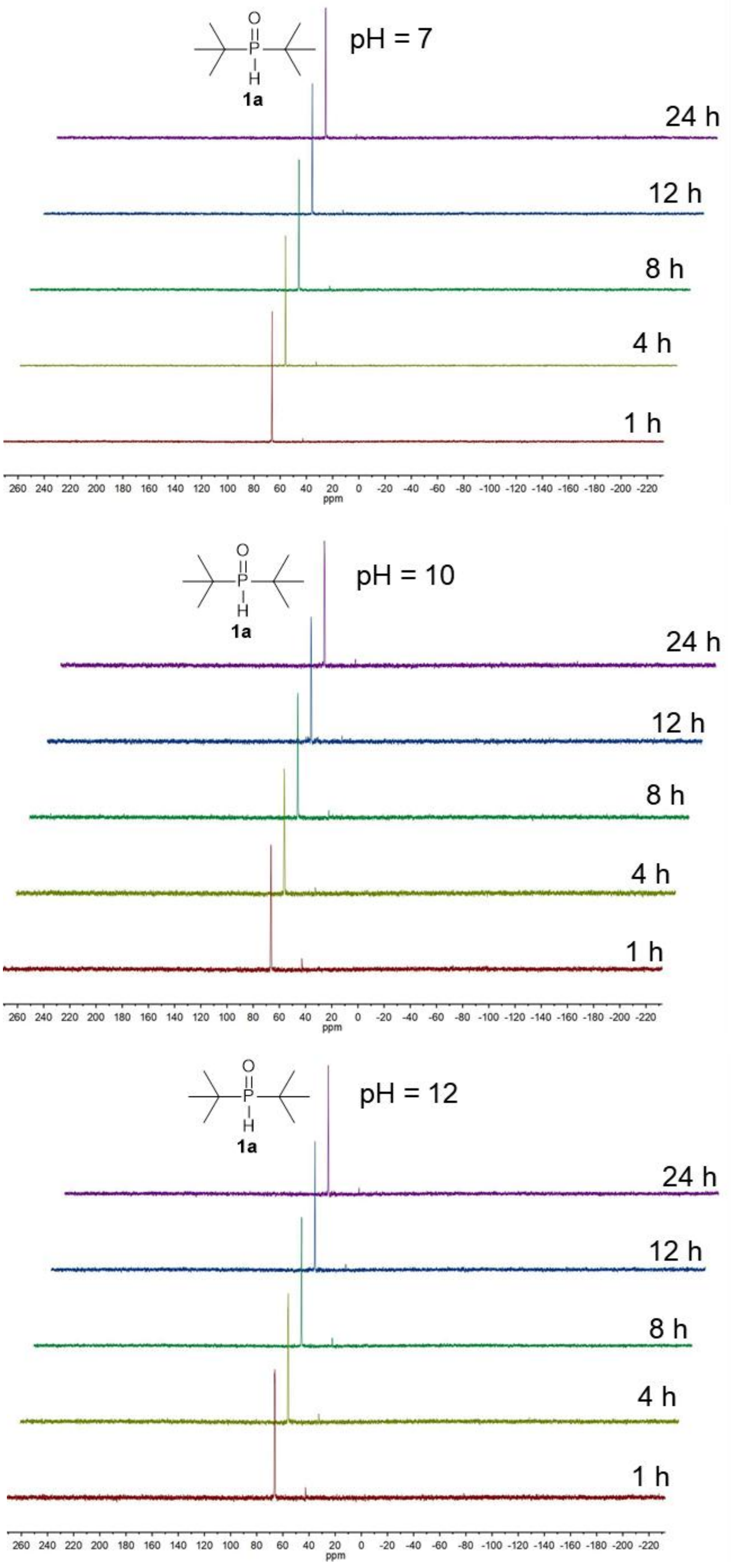

Figure S1. ^{31}P NMR analysis of stabilities from compound **1a** in different pH aqueous (pH=1, 4, 7, 10, 12).

1.2 Stability of phosphinic fluoride 1

10.0 mg of the phosphinic fluoride **1** was dissolved in a mixture of 800 μL of D_2O and 200 μL of CD_3CN and left to dissolve for transfer to the NMR tube. The ^{19}F NMR signals of the compound **1** was monitored by ^{19}F NMR for 1 h, 2 h, 4 h, 8 h, 1 d, 3 d and 9 d.

a

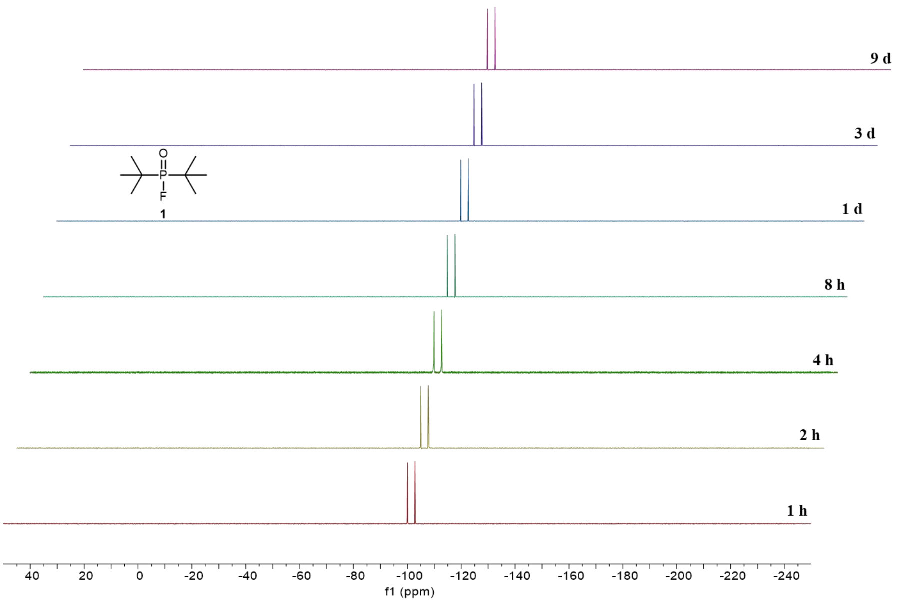

b

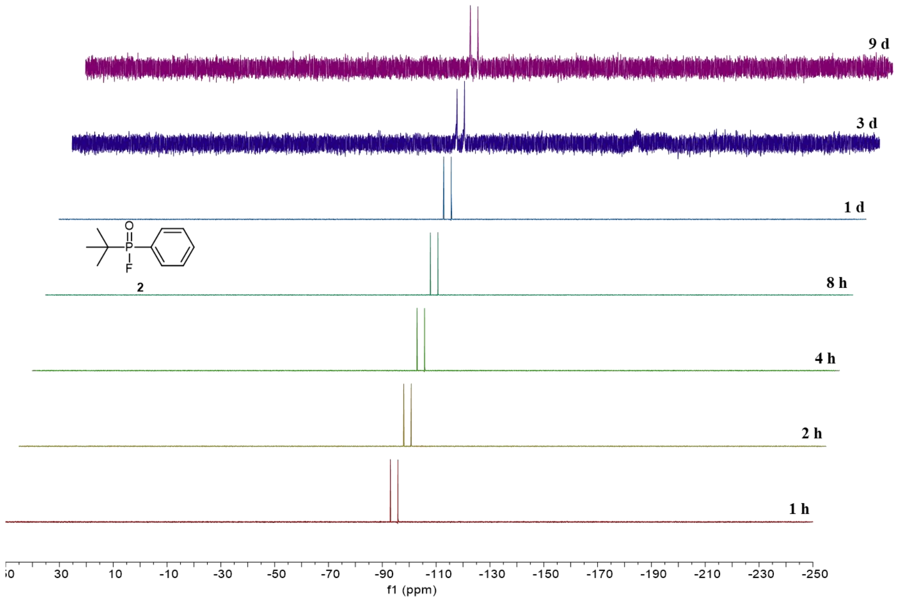

c

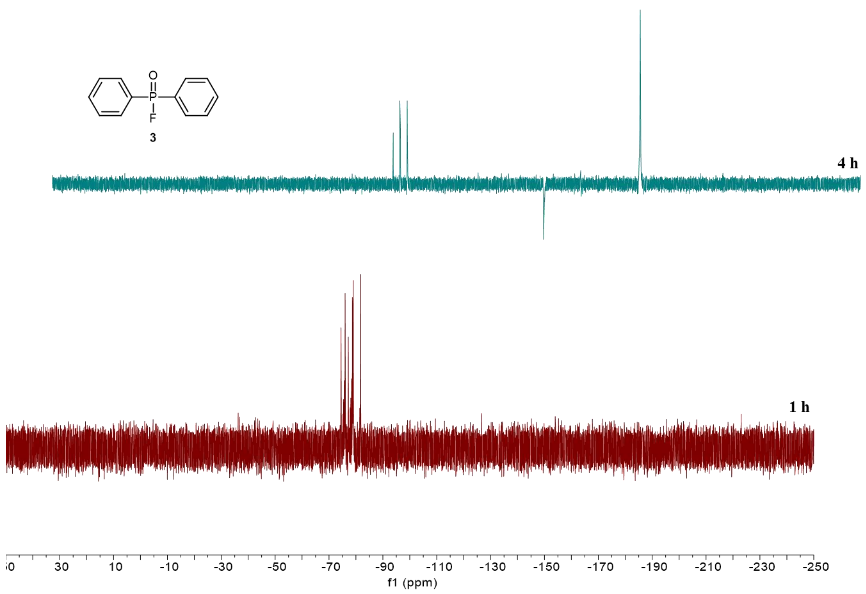

Figure S2. ^{19}F NMR analysis of stabilities from compound **1** in aqueous solution.

2 Metal salt-mediated fluorination of phosphine oxides

2.1 Screening of metal salts

2.1.1 General procedure

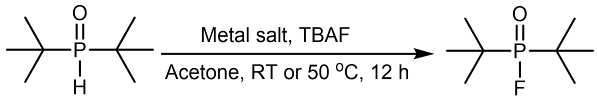

Scheme S1. General reaction route of metal salts screening.

Compound **1a** (0.0100 g, 0.0617 mmol), metal salt (2 equiv.) and were added to a 2.0 mL centrifuge tube with acetone (0.6 mL) as the reaction solvent. Each reactant was sonicated to dissolve as much as possible and then transferred to a magnetic stirrer. Then, tetrabutylammonium fluoride (2 equiv.) was added to the system, and the reaction was carried out at the corresponding temperature for 12 h. The reaction was quenched by adding saturated potassium carbonate solution to the reaction solution and centrifuged, and the supernatant was aspirated for ^{31}P NMR analysis.

2.1.2 Screening of different Cu family salts

The reaction was run according to the general procedure of **2.1.1 General procedure**. Compound **1a** (0.0100 g, 0.0617 mmol), metal salt (0.1233 mmol, wherein Cu powder 0.0078 g, CuCl 0.0122 g, $CuCl_2$ 0.0166 g, $CuBr_2$ 0.0275 g, CuI 0.0235 g, $CuSO_4{\cdot}5H_2O$ 0.0308 g, $Cu(OAc)_2$ 0.0246 g, CuO 0.0098 g, Cu_2O 0.0176 g, $Cu(OMs)_2$ 0.0869 g, $Cu(OTf)_2$ 0.0446 g, $AgNO_3$ 0.0209 g, Ag_2SO_4 0.0384 g, AgOAc 0.0206 g, AgTFA 0.0272 g, AgOTF 0.0317 g), additive (K_2CO_3, 0.0170 g, 0.1233 mmol), and TBAF (1 mol·L^{-1} in THF, 0.1233 mL, 0.1233 mmol) were added to a tube. After the reaction was completed, saturated potassium carbonate was added for extraction. The effects of different copper family salts on the fluorination conversion of compound **1** were shown in **Table S1**.

Table S1. Screening of different Cu family salts[a].

Entry	Metal salts	Additive	T (°C)	Conv. (%)[b]	Average (%)
1	Cu powder	-	25	0	0 (n = 3)
				0	
				0	
2	CuCl	-	25	trace	trace (n = 3)
				trace	
				trace	
		-	50	5.7	11.5 ± 5.4 (n = 3)
				12.4	
				16.5	
3	$CuCl_2$	-	25	47.6	72.5 ± 22.3 (n = 3)
				90.9	
				78.9	
		K_2CO_3	25	4.2	4.5 ± 0.3 (n = 3)
				4.7	
				4.5	
4	$CuBr_2$	-	25	0	1.9 ± 3.2 (n = 3)
				5.6	
				0	
		-	50	26.1	9.5 ± 11.1 (n = 4)
				3.4	
				6.0	
				2.6	
5	CuI	-	25	0	0(n = 3)
				0	
				0	
6	$CuSO_4 \cdot 5H_2O$	-	25	3.8	3.6 ± 0.3(n = 3)
				3.3	
				3.6	
		-	50	92.0	85.4 ± 5.7 (n = 3)
				82.9	
				81.4	
7	$Cu(OAc)_2$	-	25	100.0	100.0 ± 0.0 (n = 3)
				100.0	
				100.0	
8	CuO	-	25	8.3	8.6 ± 0.5 (n = 3)

		-	50	9.1<br>8.3<br>26.6<br>28.6<br>35.8	30.3 ± 4.8 (n = 3)
9	Cu_2O	-	25	1.3<br>3.3<br>2.8	2.5 ± 1.0 (n = 3)
		-	50	19.2<br>15.1<br>15.7	16.7 ± 2.2 (n = 3)
10	$Cu(OMs)_2$ (in 36.0 wt.% in H_2O)	-	25	0<br>0<br>0	0 (n = 3)
		-	50	24.7<br>27.3<br>28.1	26.7 ± 1.8 (n = 3)
11	$Cu(OTf)_2$	-	25	trace	trace (n = 1)
		-	50	32.7<br>27.3<br>32.8	30.9 ± 3.2 (n = 3)
12	$AgNO_3$	-	25	100.0<br>100.0<br>78.3	92.8 ± 12.5 (n = 3)
13	Ag_2SO_4	-	25	0<br>0<br>0	0 (n = 3)
14	AgOAc	-	25	0<br>0<br>0	0 (n = 3)
15	AgTFA	-	25	trace<br>trace<br>trace	trace (n = 3)
		-	50	27.4<br>8.9<br>7.1<br>13.0	14.4 ± 11.2 (n = 4)

16	AgOTF	-	25	0<br>0<br>0	0 (n = 3)

[a]Reaction condition: **1a** (0.0100 g, 0.0617 mmol), TBAF (2 equiv.), metal salt (2 equiv.), additive (2 equiv.), acetone (0.6 mL), 25/50 °C, 12 h. [b]Conversion was determined by ^{31}P NMR analysis.

2.1.3 Screening of different Zn salts

The reaction was run according to the general procedure of **2.1.1 General procedure**. Compound **1a** (0.0100 g, 0.0617 mmol), metal salt (0.1233 mmol, wherein $ZnCl_2$ 0.0168 g, $ZnBr_2$ 0.0278 g, $Zn(NO_3)_2 \cdot 6H_2O$ 0.0367 g, $ZnSO_4$ 0.0199 g, $Zn(OAc)_2$ 0.0206 g, $Zn(OTf)_2$ 0.0448 g, ZnO 0.0100 g) and TBAF (1 mol·L^{-1} in THF, 0.1233 mL, 0.1233 mmol) were added to a tube. The reaction solution was filtered and used for NMR analysis. The effects of different zinc family salts on the fluorination conversion of compound **1** were shown in **Table S2**.

Table S2. Screening of different Zn salts[a].

Entry	Metal salts	T (°C)	Conv. (%)[b]
1	$ZnCl_2$	25	0 (n = 3)
2	$ZnBr_2$	25	0 (n = 3)
3	$Zn(NO_3)_2 \cdot 6H_2O$	25	0 (n = 3)
4	$ZnSO_4$	25	0 (n = 3)
5	$Zn(OAc)_2$	25	0 (n = 3)
		50	0 (n = 3)
6	$Zn(OTf)_2$	25	0 (n = 3)
7	ZnO	25	0 (n = 3)

[a]Reaction condition: **1a** (0.0100 g, 0.0617 mmol), TBAF (2 equiv.), metal salt (2 equiv.), acetone (0.6 mL), 25/50 °C, 12 h. [b]Conversion was determined by ^{31}P NMR analysis.

2.1.4 Screening of different transition metal salts

The reaction was run according to the general procedure of **2.1.1 General procedure**. Compound **1a** (0.0100 g, 0.0617 mmol), metal salt (0.1233 mmol, wherein $FeCl_3 \cdot 6H_2O$ 0.0200 g, $FeSO_4$ 0.0187 g) g, $Fe(OAc)_3 \cdot nH_2O$ 0.0235 g, Fe_3O_4 0.0285 g, $Fe(OTf)_3$ 0.0620 g, $NiCl_2$ 0.0160 g, $Ni(acac)_2$ 0.0317 g, $MnCl_2$ 0.0155 g), and TBAF (1 mol·L^{-1} in THF, 0.1233 mL, 0.1233 mmol) were added to a tube. The

post-treatment of iron salts and nickel salts were extracted with saturated brine, then dried with anhydrous sodium sulfate, and the upper organic solvent was taken for NMR analysis; the post-treatment of manganese salt was extracted with saturated potassium carbonate, dried and then subjected to NMR analysis. The effects of different transition metal salts on the fluorination conversion of compound **1** were shown in **Table S3**.

Table S3. Screening of different transition metal salts[a].

Entry	Metal salt	T (°C)	Conv. (%)[b]
1	$FeCl_3{\cdot}6H_2O$	25	0 (n = 3)
		50	0 (n = 3)
2	$FeSO_4$	25	0 (n = 3)
3	$Fe(OAc)_3{\cdot}nH_2O$	25	0 (n = 3)
4	Fe_3O_4	25	0 (n = 1)
5	$Fe(OTf)_3$	25	0 (n = 3)
		50	0 (n = 4)
6	$NiCl_2$	25	0 (n = 3)
7	$Ni(acac)_2$	25	0 (n = 4)
8	$MnCl_2$	25	0 (n = 3)

[a]Reaction condition: **1a** (0.0100 g, 0.0617 mmol), TBAF (2 equiv.), metal salt (2 equiv.), acetone (0.6 mL), 25/50 °C, 12 h. [b]Conversion was determined by ^{31}P NMR analysis.

2.1.5 Screening of different Pd and Pt metal salts

The reaction was run according to the general procedure of **2.1.1 General procedure**. Compound **1a** (0.0100 g, 0.0617 mmol), metal salt (0.1233 mmol, wherein $PdCl_2$ 0.0219 g, $Pd(OAc)_2$ 0.0277 g, $Pd(acac)_2$ 0.0376 g, $Pd(dba)_2$ 0.0709 g, $PtCl_2$ 0.0416), TBAF (1mol·L^{-1} in THF, 0.1233 mL, 0.1233 mmol) were added to a tube. After the reaction was completed, the reaction solution was filtered and analyzed by NMR. The effects of different platinum metal compounds on the fluorination conversion of compound **1** were shown in **Table S4**.

Table S4. Screening of different Pd and Pt metal salts[a].

Entry	Metal salt	T (°C)	Conv. (%)[b]	Average (%)
1	$PdCl_2$	25	0	0 (n = 3)
			0	
			0	
		50	0	0 (n = 3)
			0	
			0	
2	$Pd(OAc)_2$	25	0	0 (n = 3)
			0	
			0	
		50	0	0.7 ± 1.1 (n = 3)
			2.0	
			0	
3	$Pd(acac)_2$	25	0	0 (n = 3)
			0	
			0	
4	$Pd(dba)_2$	25	0	0 (n = 3)
			0	
			0	
5	$PtCl_2$	25	0	0 (n = 3)
			0	
			0	

[a]Reaction condition: **1a** (0.0100 g, 0.0617 mmol), TBAF (2 equiv.), metal salt (2 equiv.), acetone (0.6 mL), 25/50 °C, 12 h. [b]Conversion was determined by ^{31}P NMR analysis.

2.2 Optimization of $Cu(OAc)_2$, $CuCl_2$ and $AgNO_3$-mediated fluorination reaction conditions

2.2.1 Optimization of $Cu(OAc)_2$-mediated fluorination reaction conditions

Table S5. Optimization of reaction conditions^{a}.

Experiment batch	Entry	Salt (equiv.)	Solvent	Fluorine source	Additive	Conv.^{b}
I	1	$Cu(OAc)_2$ (5 mol%)	Acetone	TBAF	None	0%
	2	$Cu(OAc)_2$ (10 mol%)	Acetone	TBAF	None	0%
	3	$Cu(OAc)_2$ (1)	Acetone	TBAF	None	41%
	4	$Cu(OAc)_2$ (1.5)	Acetone	TBAF	None	98%
	5	$Cu(OAc)_2$ (2)	Acetone	TBAF	None	100%
	6	$Cu(OAc)_2$ (3)	Acetone	TBAF	None	100%
II	7	$Cu(OAc)_2$ (2)	Acetone	KF	None	0%
	8	$Cu(OAc)_2$ (2)	Acetone	NaF	None	Trace
	9	$Cu(OAc)_2$ (2)	Acetone	CsF	None	Trace
	10	$Cu(OAc)_2$ (2)	Acetone	AgF	None	2%
III	11	$Cu(OAc)_2$ (2)	CH_3CN	TBAF	None	58%
	12	$Cu(OAc)_2$ (2)	THF	TBAF	None	99%
	13	$Cu(OAc)_2$ (2)	DMF	TBAF	None	100%
	14	$Cu(OAc)_2$ (2)	DMSO	TBAF	None	100%
	15	$Cu(OAc)_2$ (2)	CH_3OH	TBAF	None	1%
	16	$Cu(OAc)_2$ (2)	CH_3CH_2OH	TBAF	None	13%
IV	17	$Cu(OAc)_2$ (2)	Acetone	TBAF	TEA	97%
	18	$Cu(OAc)_2$ (2)	Acetone	TBAF	Py	97%
	19	$Cu(OAc)_2$ (2)	Acetone	TBAF	DBU	98%
	20	$Cu(OAc)_2$ (2)	Acetone	TBAF	AcOH	38%

^{a}Reaction conditions: **1a** (0.0100 g), $Cu(OAc)_2$ (0.05–3 equiv.), solvent (0.5 mL), fluorine source (2 equiv.), additive (2 equiv.), 1 h. ^{b}Conversion was determined by ^{31}P NMR analysis.

2.2.2 Optimization of $CuCl_2$ -mediated fluorination reaction conditions

Table S6. Optimization of reaction conditions[a].

Experiment batch	Entry	Salt (equiv.)	Solvent	Fluorine source	Additive	Conv. [b]
I	1	$CuCl_2$ (5 mol%)	Acetone	TBAF	None	0%
	2	$CuCl_2$ (10 mol%)	Acetone	TBAF	None	0%
	3	$CuCl_2$ (1)	Acetone	TBAF	None	28%
	4	$CuCl_2$ (2)	Acetone	TBAF	None	97%
	5	$CuCl_2$ (3)	Acetone	TBAF	None	98%
II	6	$CuCl_2$ (2)	Acetone	KF	None	45%
	7	$CuCl_2$ (2)	Acetone	AgF	None	9%
	8	$CuCl_2$ (2)	Acetone	CsF	None	27%
	9	$CuCl_2$ (2)	Acetone	NaF	None	Trace
	10	$CuCl_2$ (2)	Acetone	Me_4NF	None	0%
III	11	$CuCl_2$ (2)	CH_3CN	TBAF	None	71%
	12	$CuCl_2$ (2)	THF	TBAF	None	77%
	13	$CuCl_2$ (2)	DMF	TBAF	None	83%
	14	$CuCl_2$ (2)	DMSO	TBAF	None	80%
	15	$CuCl_2$ (2)	CH_3OH	TBAF	None	53%
IV	16	$CuCl_2$ (2)	Acetone	TBAF	TEA	13%
	17	$CuCl_2$ (2)	Acetone	TBAF	Py	29%
	18	$CuCl_2$ (2)	Acetone	TBAF	DBU	32%
	19	$CuCl_2$ (2)	Acetone	TBAF	AcOH	63%

[a]Reaction conditions: **1a** (0.0100 g), $CuCl_2$ (0.05–3 equiv.), solvent (0.5 mL), fluorine source (2 equiv.), additive (2 equiv.), 12 h. [b]Conversion was determined by ^{31}P NMR analysis.

2.2.3 Optimization of $AgNO_3$-mediated fluorination reaction condition

Table S7. Optimization of reaction conditions[a].

Experiment batch	Entry	Salt (equiv.)	Solvent	Fluorine source	Additive	Conv.[b]
I	1	$AgNO_3$ (5 mol%)	Acetone	TBAF	None	0%
	2	$AgNO_3$ (10 mol%)	Acetone	TBAF	None	0%
	3	$AgNO_3$ (1)	Acetone	TBAF	None	0%
	4	$AgNO_3$ (2)	Acetone	TBAF	None	Trace
	5	$AgNO_3$ (3)	Acetone	TBAF	None	100%
II	6	$AgNO_3$ (3)	Acetone	KF	None	72%
	7	$AgNO_3$ (3)	Acetone	AgF	None	100%
	8	$AgNO_3$ (3)	Acetone	CsF	None	100%
	9	$AgNO_3$ (3)	Acetone	NaF	None	5%
	10	$AgNO_3$ (3)	Acetone	Me_4NF	None	96%
III	11	$AgNO_3$ (3)	CH_3CN	TBAF	None	21%
	12	$AgNO_3$ (3)	THF	TBAF	None	53%
	13	$AgNO_3$ (3)	DMF	TBAF	None	53%
	14	$AgNO_3$ (3)	DMSO	TBAF	None	0%
	15	$AgNO_3$ (3)	CH_3OH	TBAF	None	21%
IV	16	$AgNO_3$ (3)	Acetone	TBAF	TEA	23%
	17	$AgNO_3$ (3)	Acetone	TBAF	Py	22%
	18	$AgNO_3$ (3)	Acetone	TBAF	DBU	0%
	19	$AgNO_3$ (3)	Acetone	TBAF	AcOH	0%

[a]Reaction conditions: **1a** (0.0100 g), $AgNO_3$ (0.05–3 equiv.), solvent (0.5 mL), fluorine source (2 equiv.), additive (2 equiv.), 12 h. [b]Conversion was determined by ^{31}P NMR analysis.

2.2.4 Comparison of $Cu(OAc)_2$, $CuCl_2$ and $AgNO_3$-mediated fluorination reaction conversions with time

Table S8. The conversion of $Cu(OAc)_2$-mediated fluorination reaction at different times[a].

Time (min)	5	15	30	45	60
Conv.[b] (%)	17	35	73	95	100

[a]Reaction conditions: **1a** (10 mg), $Cu(OAc)_2$ (2 equiv.), acetone (0.5 mL), TBAF (2 equiv.), RT.
[b]Conversion was determined by ^{31}P NMR analysis.

Table S9. The conversion of $CuCl_2$-mediated fluorination reaction at different times[a].

Time (h)	0.5	1	4	7	12
Conv.[b] (%)	33	40	83	88	97

[a]Reaction conditions: **1a** (10 mg), $CuCl_2$ (2 equiv.), acetone (0.5 mL), TBAF (2 equiv.), RT.
[b]Conversion was determined by ^{31}P NMR analysis.

Table S10. The conversion of $AgNO_3$-mediated fluorination reaction at different times[a].

Time (h)	0.5	1	4	7	12
Conv.[b] (%)	3	9	42	69	100

[a]Reaction conditions: **1a** (10 mg), $AgNO_3$ (2 equiv.), acetone (0.5 mL), TBAF (2 equiv.), RT.
[b]Conversion was determined by ^{31}P NMR analysis.

2.3 Characterization spectrum of ^{31}P NMR

The ^{31}P NMR analysis results of each metal salt screened were shown in **Figure S3**, in which the chemical shift of ^{31}P of compound **1a** is around 63.45 ppm (doublet, not decoupled; singlet, decoupled), and the chemical shift of compound **1** is around 76.68 ppm (doublet).

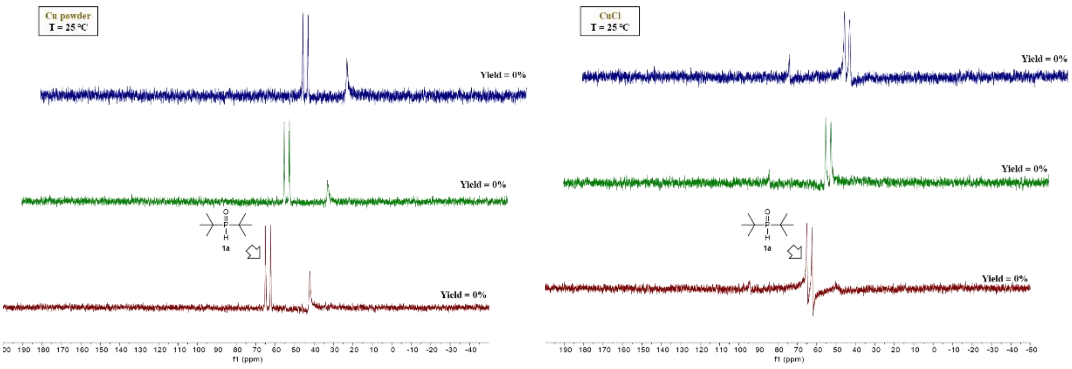

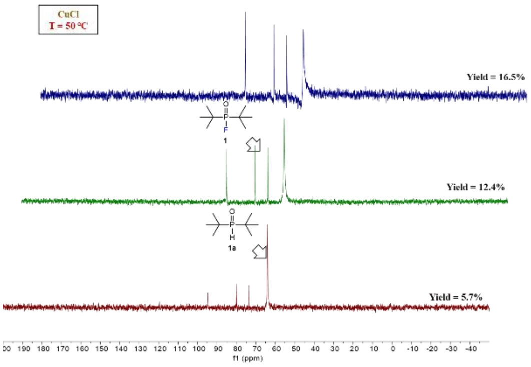
CuCl
T = 50 °C
Yield = 16.5%
Yield = 12.4%
Yield = 5.7%
1
1a
f1 (ppm)

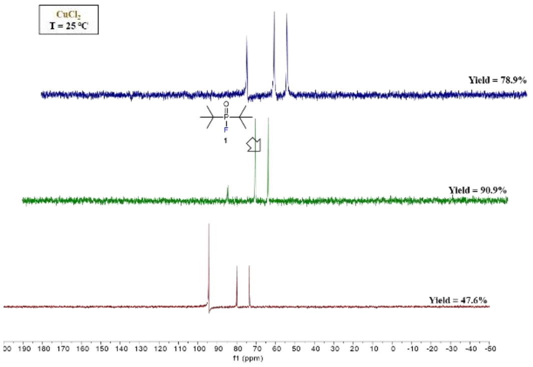
$CuCl_2$
T = 25 °C
Yield = 78.9%
Yield = 90.9%
Yield = 47.6%
1
f1 (ppm)

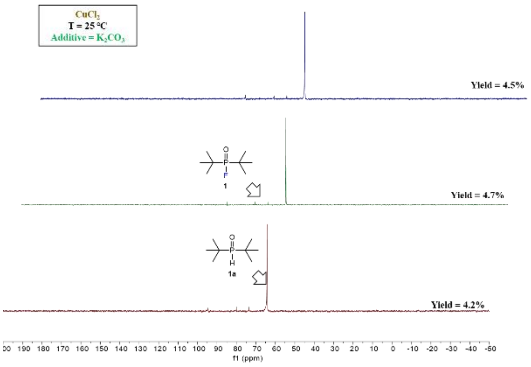
$CuCl_2$
T = 25 °C
Additive = K_2CO_3
Yield = 4.5%
Yield = 4.7%
Yield = 4.2%
1
1a
f1 (ppm)

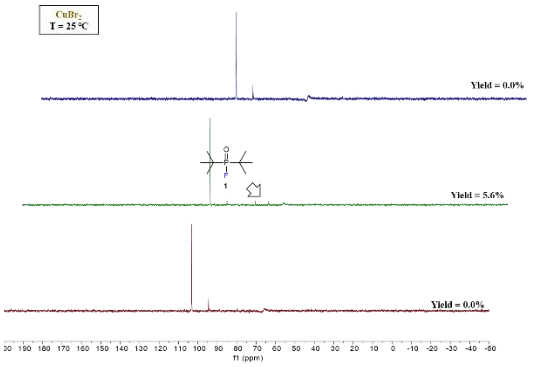
$CuBr_2$
T = 25 °C
Yield = 0.0%
Yield = 5.6%
Yield = 0.0%
1
f1 (ppm)

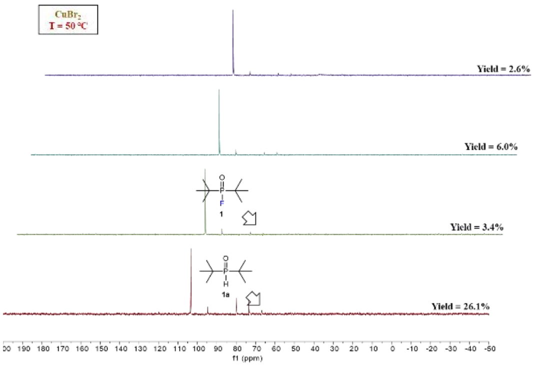
$CuBr_2$
T = 50 °C
Yield = 2.6%
Yield = 6.0%
Yield = 3.4%
Yield = 26.1%
1
1a
f1 (ppm)

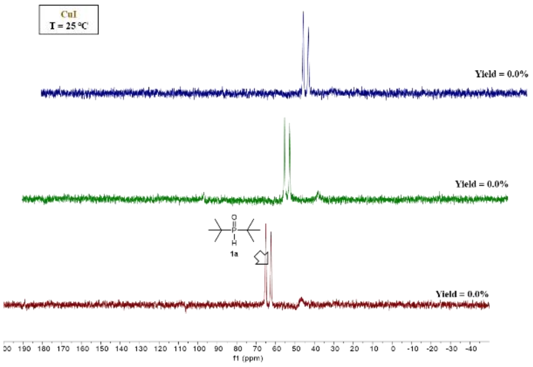
CuI
T = 25 °C
Yield = 0.0%
Yield = 0.0%
Yield = 0.0%
1a
f1 (ppm)

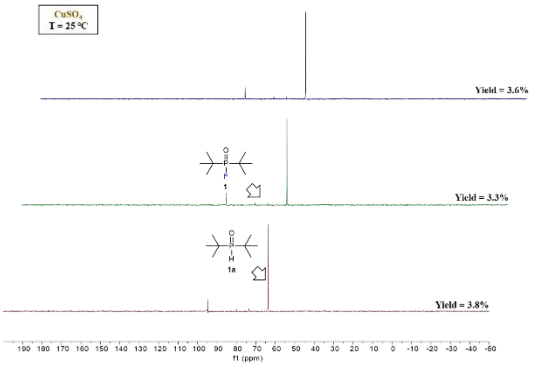
$CuSO_4$
T = 25 °C
Yield = 3.6%
Yield = 3.3%
Yield = 3.8%
1
1a
f1 (ppm)

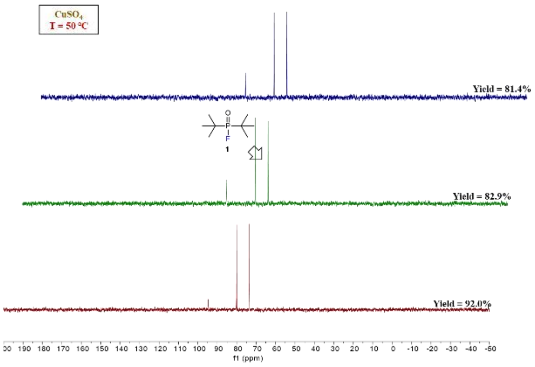
$CuSO_4$
T = 50 °C
Yield = 81.4%
Yield = 82.9%
Yield = 92.0%
1
f1 (ppm)

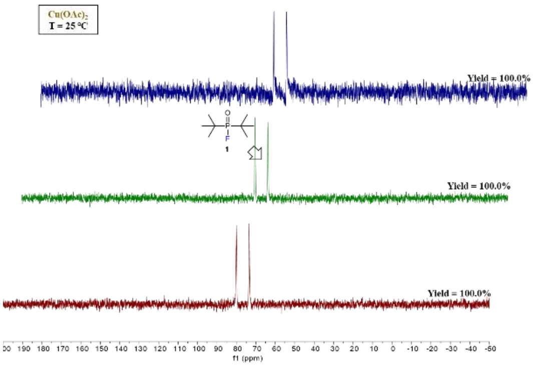
Cu(OAc)2
T = 25 ºC
Yield = 100.0%
1
Yield = 100.0%
Yield = 100.0%
f1 (ppm)

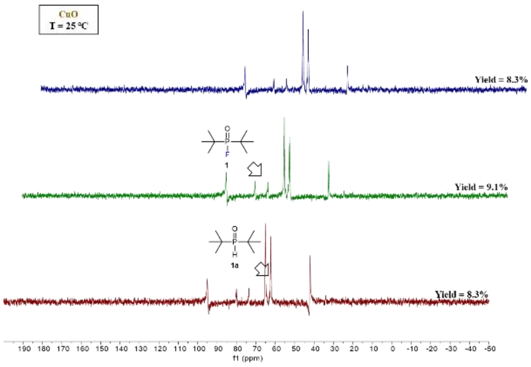
CuO
T = 25 ºC
Yield = 8.3%
1
Yield = 9.1%
1a
Yield = 8.3%
f1 (ppm)

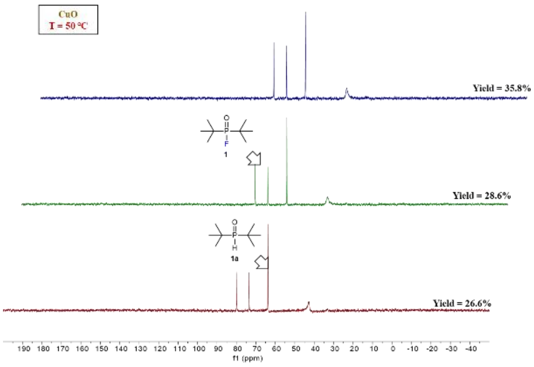
CuO
T = 50 ºC
Yield = 35.8%
1
Yield = 28.6%
1a
Yield = 26.6%
f1 (ppm)

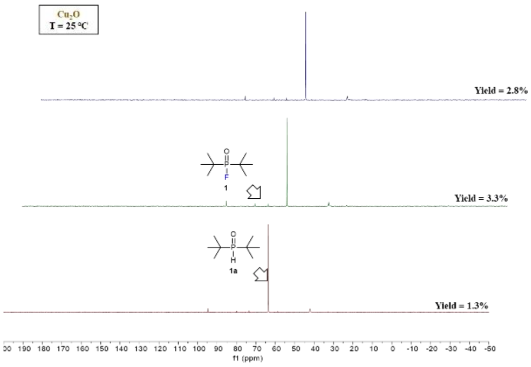
Cu2O
T = 25 ºC
Yield = 2.8%
1
Yield = 3.3%
1a
Yield = 1.3%
f1 (ppm)

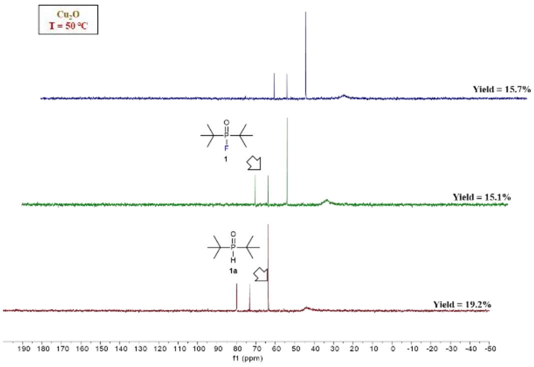
Cu2O
T = 50 ºC
Yield = 15.7%
1
Yield = 15.1%
1a
Yield = 19.2%
f1 (ppm)

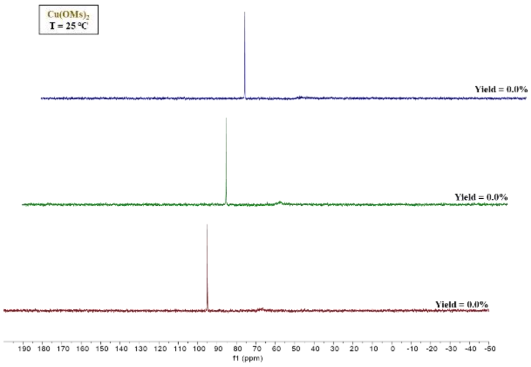
Cu(OMs)2
T = 25 ºC
Yield = 0.0%
Yield = 0.0%
Yield = 0.0%
f1 (ppm)

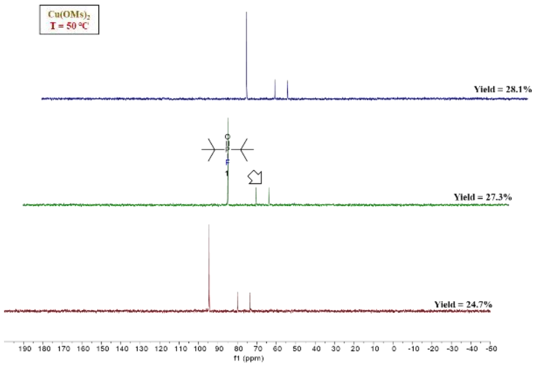
Cu(OMs)2
T = 50 ºC
Yield = 28.1%
1
Yield = 27.3%
Yield = 24.7%
f1 (ppm)

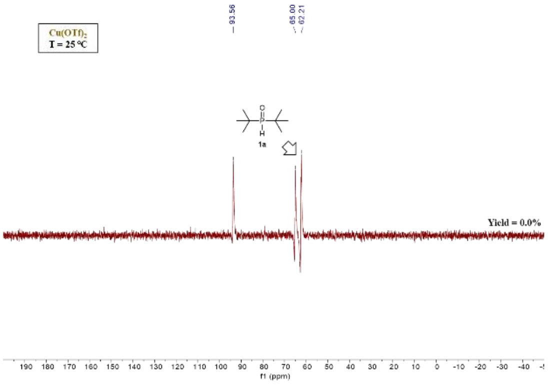
Cu(OTf)2
T = 25 ºC
93.56
65.00
62.21
1a
Yield = 0.0%
f1 (ppm)

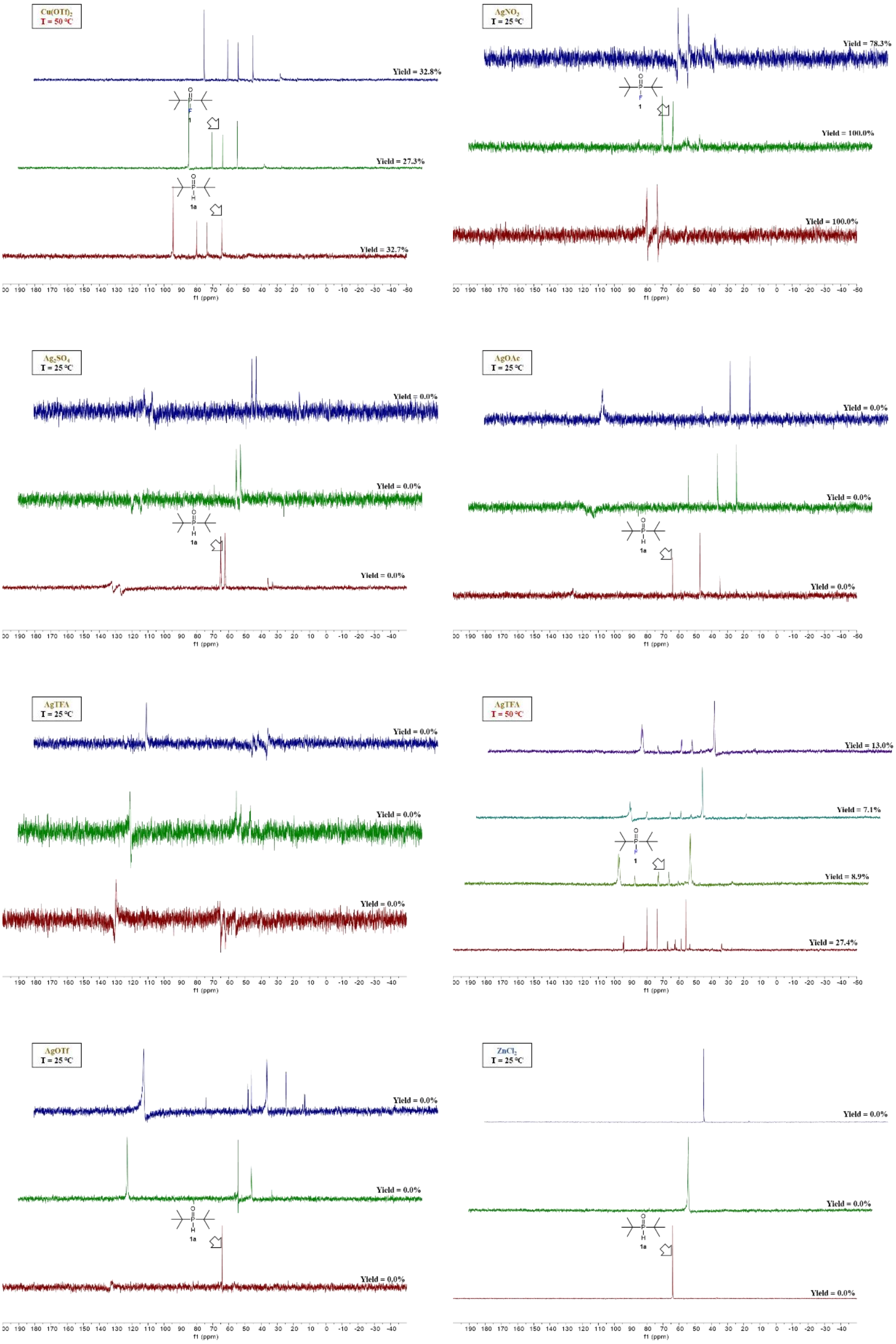
Cu(OTf)$_2$
T = 50 °C
Yield = 32.8%
Yield = 27.3%
Yield = 32.7%
1
1a
AgNO$_3$
T = 25 °C
Yield = 78.3%
Yield = 100.0%
Yield = 100.0%
1
Ag$_2$SO$_4$
T = 25 °C
Yield = 0.0%
Yield = 0.0%
Yield = 0.0%
1a
AgOAc
T = 25 °C
Yield = 0.0%
Yield = 0.0%
Yield = 0.0%
1a
AgTFA
T = 25 °C
Yield = 0.0%
Yield = 0.0%
Yield = 0.0%
AgTFA
T = 50 °C
Yield = 13.0%
Yield = 7.1%
Yield = 8.9%
Yield = 27.4%
1
AgOTf
T = 25 °C
Yield = 0.0%
Yield = 0.0%
Yield = 0.0%
1a
ZnCl$_2$
T = 25 °C
Yield = 0.0%
Yield = 0.0%
Yield = 0.0%
1a
f1 (ppm)

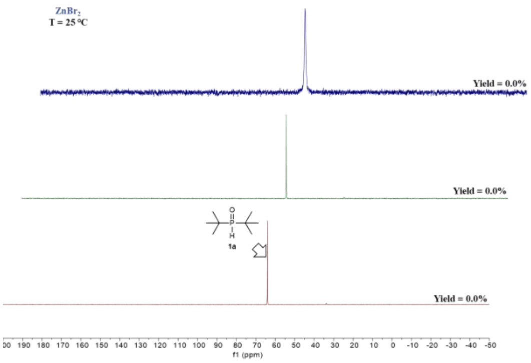
ZnBr$_2$
T = 25 °C
Yield = 0.0%
Yield = 0.0%
1a
Yield = 0.0%
f1 (ppm)

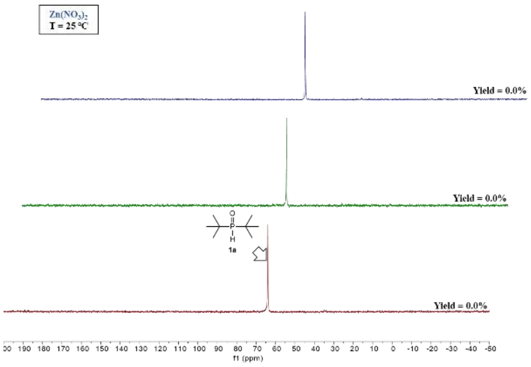
Zn(NO$_3$)$_2$
T = 25 °C
Yield = 0.0%
Yield = 0.0%
1a
Yield = 0.0%
f1 (ppm)

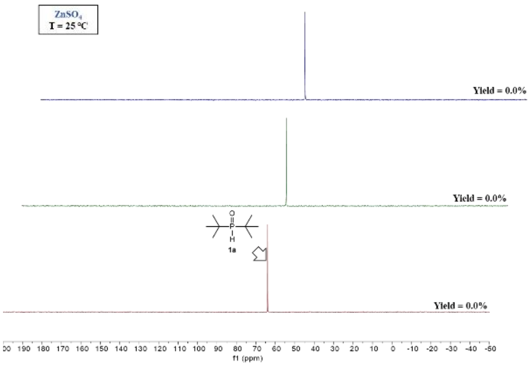
ZnSO$_4$
T = 25 °C
Yield = 0.0%
Yield = 0.0%
1a
Yield = 0.0%
f1 (ppm)

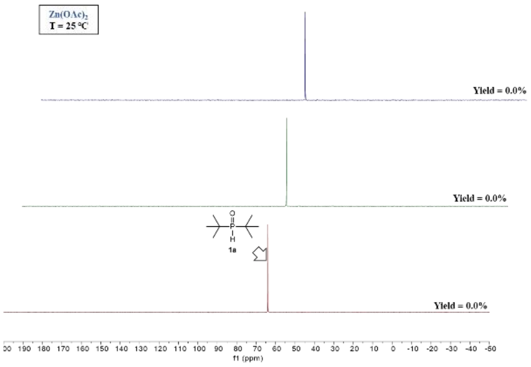
Zn(OAc)$_2$
T = 25 °C
Yield = 0.0%
Yield = 0.0%
1a
Yield = 0.0%
f1 (ppm)

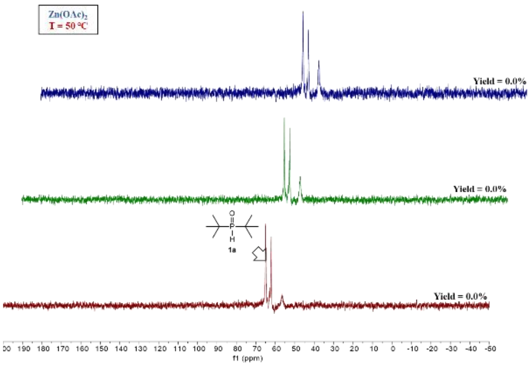
Zn(OAc)$_2$
T = 50 °C
Yield = 0.0%
Yield = 0.0%
1a
Yield = 0.0%
f1 (ppm)

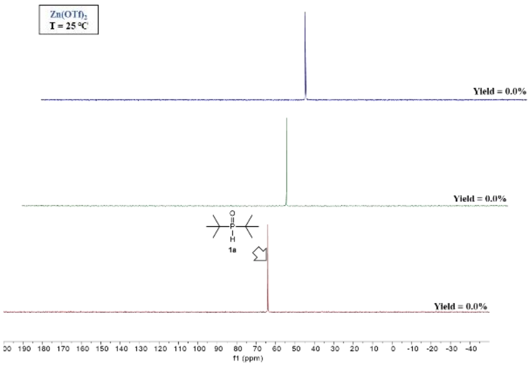
Zn(OTf)$_2$
T = 25 °C
Yield = 0.0%
Yield = 0.0%
1a
Yield = 0.0%
f1 (ppm)

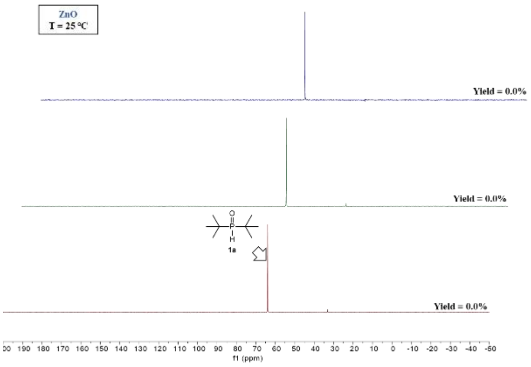
ZnO
T = 25 °C
Yield = 0.0%
Yield = 0.0%
1a
Yield = 0.0%
f1 (ppm)

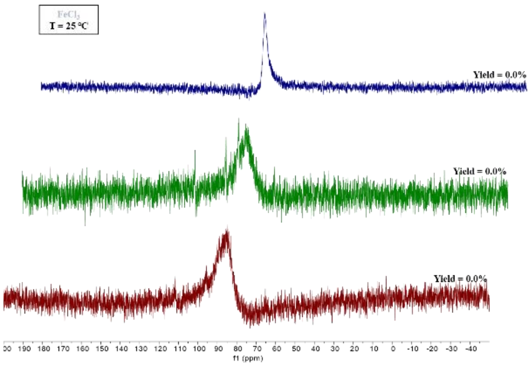
FeCl$_3$
T = 25 °C
Yield = 0.0%
Yield = 0.0%
Yield = 0.0%
f1 (ppm)

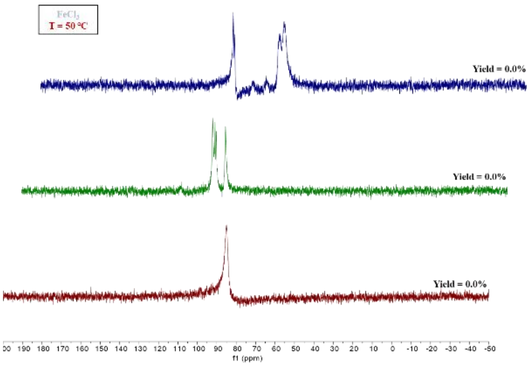
FeCl2
T = 50 °C
Yield = 0.0%
Yield = 0.0%
Yield = 0.0%
f1 (ppm)

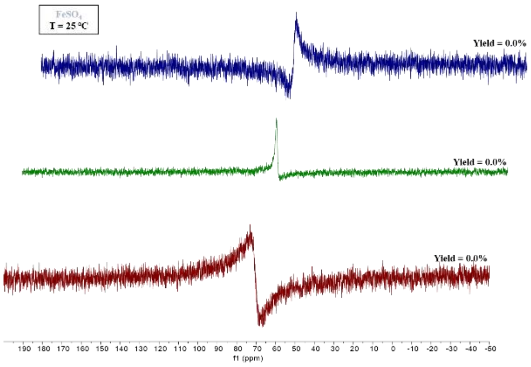
FeSO4
T = 25 °C
Yield = 0.0%
Yield = 0.0%
Yield = 0.0%
f1 (ppm)

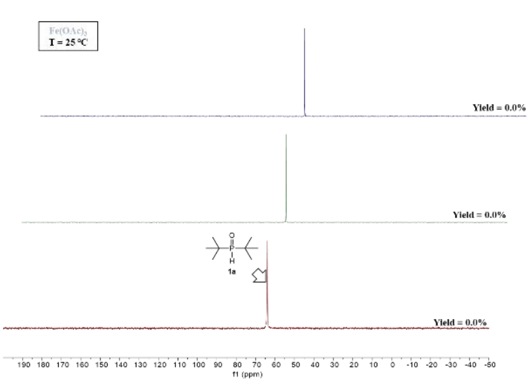
Fe(OAc)2
T = 25 °C
Yield = 0.0%
Yield = 0.0%
1a
Yield = 0.0%
f1 (ppm)

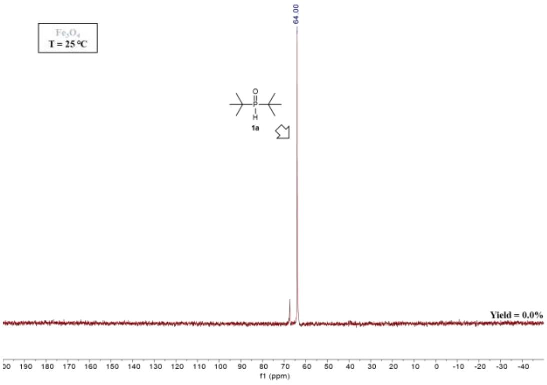
Fe3O4
T = 25 °C
64.00
1a
Yield = 0.0%
f1 (ppm)

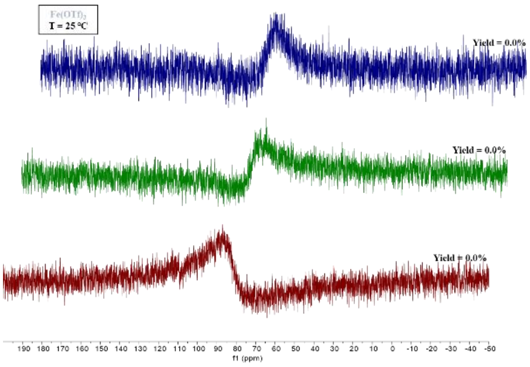
Fe(OTf)2
T = 25 °C
Yield = 0.0%
Yield = 0.0%
Yield = 0.0%
f1 (ppm)

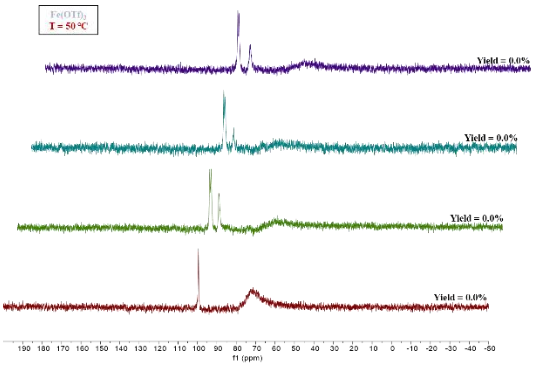
Fe(OTf)2
T = 50 °C
Yield = 0.0%
Yield = 0.0%
Yield = 0.0%
Yield = 0.0%
f1 (ppm)

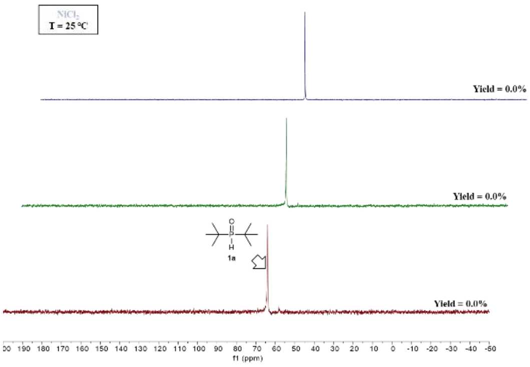
NiCl2
T = 25 °C
Yield = 0.0%
Yield = 0.0%
1a
Yield = 0.0%
f1 (ppm)

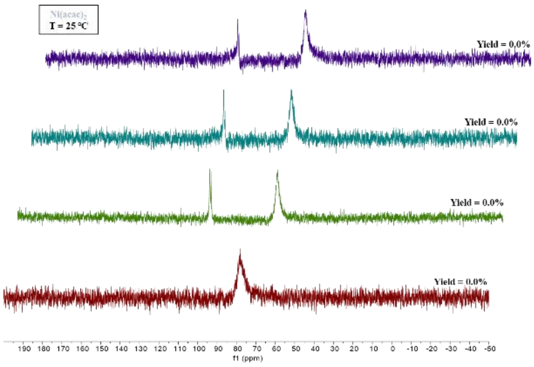
Ni(acac)2
T = 25 °C
Yield = 0.0%
Yield = 0.0%
Yield = 0.0%
Yield = 0.0%
f1 (ppm)

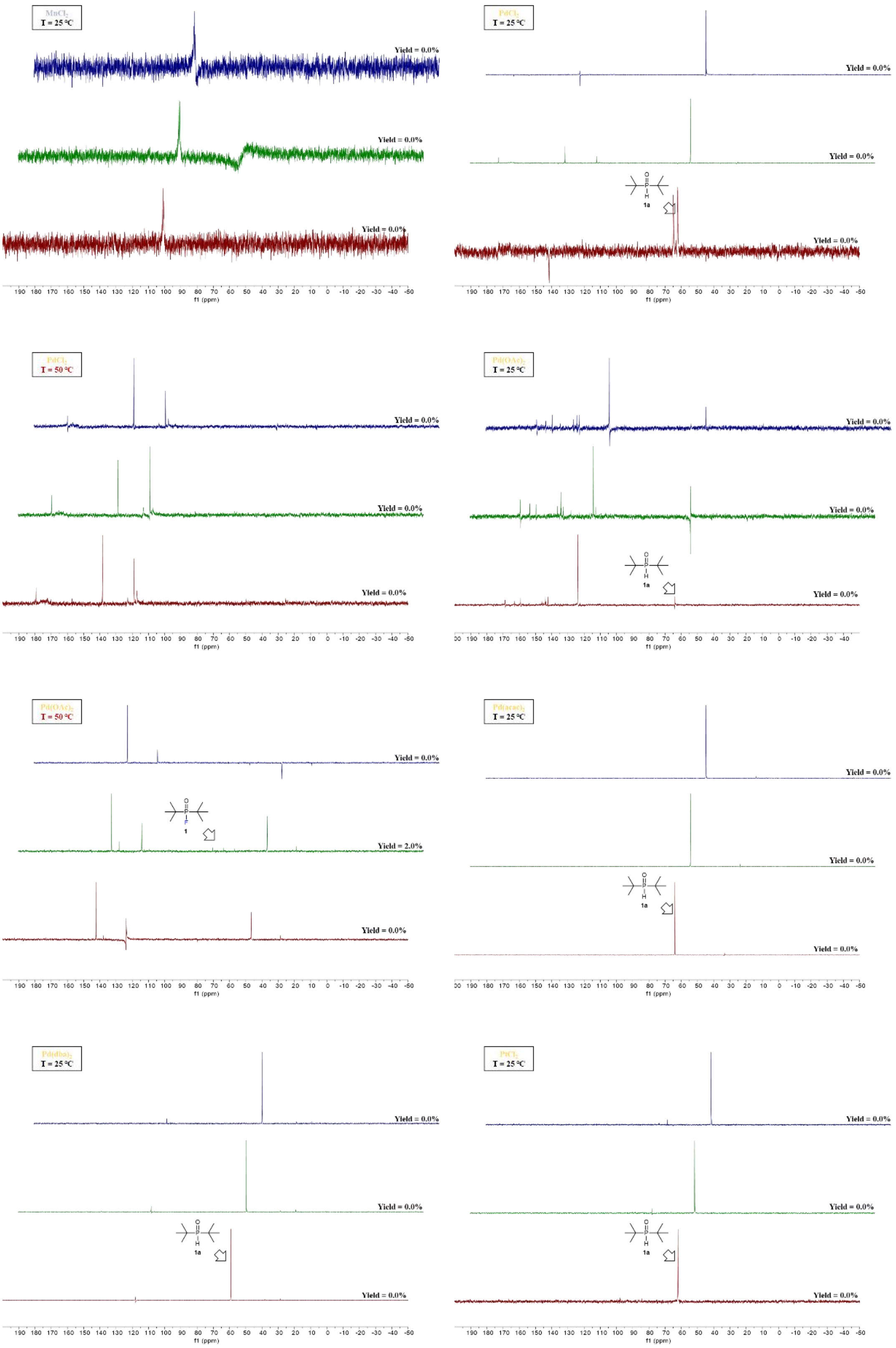

Figure S3. ^{31}P NMR analysis of substrate conversion on catalysts screening (Paramagnetic ions which are hard to remove can explain some abnormal peak shape).

2.4 Mechanism study of $Cu(OAc)_2$-mediated dehydrofluorination on phosphine oxides

Compound **1a** (0.0100 g, 0.0617 mmol), $Cu(OAc)_2$ (0.1233 mmol) , TBAF (1 mol·L^{-1} in THF, 0.1233 mL, 0.1233 mmol), and additives (TEMPO, BHT, AcOH, TEA, Py or DBU, 2-3 equiv.), were added to a 2 mL tube with acetone (0.5 mL) as solvent and transferred to a magnetic stirrer. Then, TBAF (1 mol·L^{-1} in THF, 0.1233 mL, 0.1233 mmol) was added to the mixture and stirred at room temperature for 12 h. When the reaction was finished, the resulting mixture was extracted with saturated potassium carbonate solution, and the supernatant was collected for ^{31}P NMR analysis. The conversion of the compound **1** was shown in **Table S11**.

Table S11. The effects of additives on conversion.

Entry	Additive (equiv.)	Conv. (%)
1	-	100
2	TEMPO (3)	100
3	BHT (3)	100
4	AcOH (2)	38
5	TEA (2)	97
6	Py (2)	97
7	DBU (2)	98

2.5 Effects of water content on the efficiency of copper catalytic fluorination

2.5.1 General reaction route

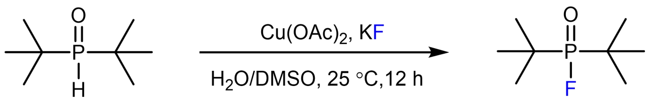

1a **1**

Scheme S2. General reaction route of water addition study.

2.5.2 General procedure for stoichiometric water addition

Compound **1a** (0.0100 g, 0.0617 mmol), $Cu(OAc)_2$ (0.0246 g, 0.1233 mmol) and KF (0.0072 g, 0.1233 mmol) were added to a tube with DMSO (0.4 mL) as reaction solvent, and then pure water was added to the system according to the corresponding stoichiometric ratio. The resulting mixture was stirred at room temperature.

Table S12. The effects of stoichiometric water addition on substrate conversion[a].

Entry	Stoichiometric ratio (H_2O : **1a**)	Conv. (%)[b]
1	10 : 1	69.8
2	30 : 1	26.8
3	50 : 1	29.5
4	70 : 1	13.2
5	90 : 1	6.8
6	100 : 1	7.2

[a]Reaction condition: **1a** (10 mg), KF (2 equiv.), $Cu(OAc)_2$ (2 equiv.), H_2O (10–100 equiv.), DMSO (0.4 ml), 25 °C, 12 h. [b]Conversion was determined by ^{31}P NMR analysis.

2.5.3 General procedure for volumetric water addition

Compound **1a** (0.0100 g, 0.0617 mmol), $Cu(OAc)_2$ (0.0246 g, 0.1233 mmol) and KF (0.0072 g, 0.1233 mmol) were added to a tube with H_2O-DMSO (0.4 mL, configured according to the corresponding volume ratio). The resulting mixture was stirred at room temperature.

Table S13. The effect of volumetric water addition on substrate conversion[a].

Entry	Volume ratio (H_2O : DMSO)	Conv. (%)[b]
1	10 : 90	2.7
2	30 : 70	2.8
3	50 : 50	0
4	70 : 30	0
5	90 : 10	0
6	100 : 0	0

[a]Reaction condition: **1a** (10 mg), KF (2 equiv.), $Cu(OAc)_2$ (2 equiv.), $H_2O_{(v)}$: $DMSO_{(v)}$ = 10 : 90 / 30 : 70 / 50 : 50 / 70 : 30 / 90 : 10 / 100 : 0, 25 °C, 12 h. [b]Conversion was determined by ^{31}P NMR analysis.

2.5.4 ^{31}P NMR analysis of substrate conversion on water addition study

a

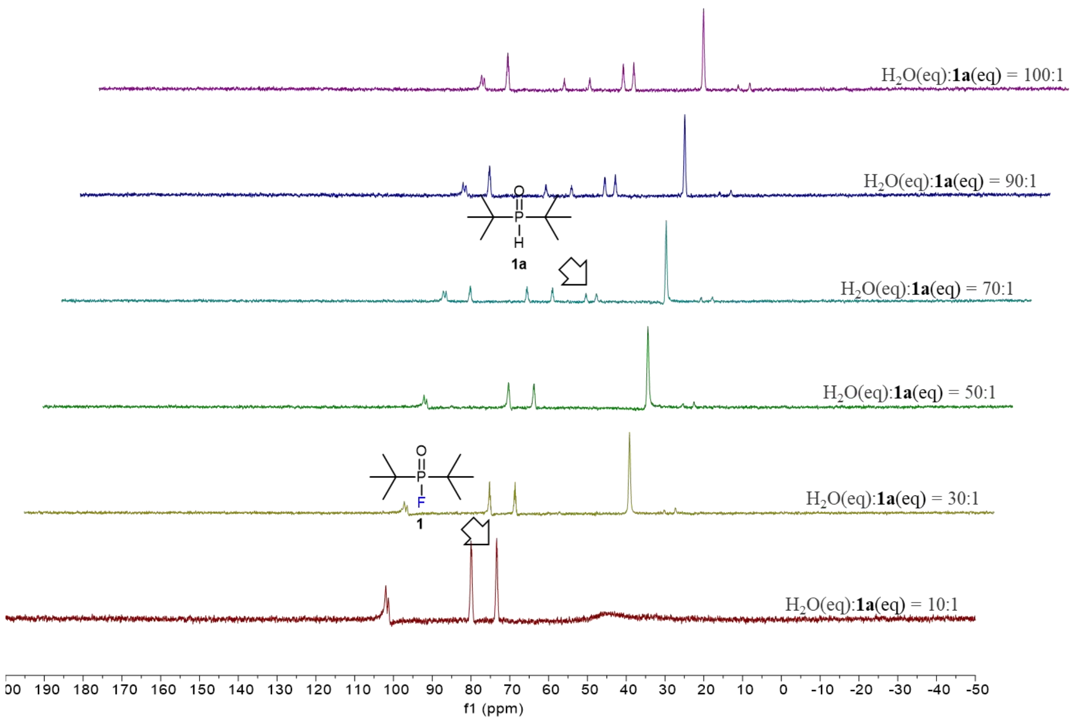

b

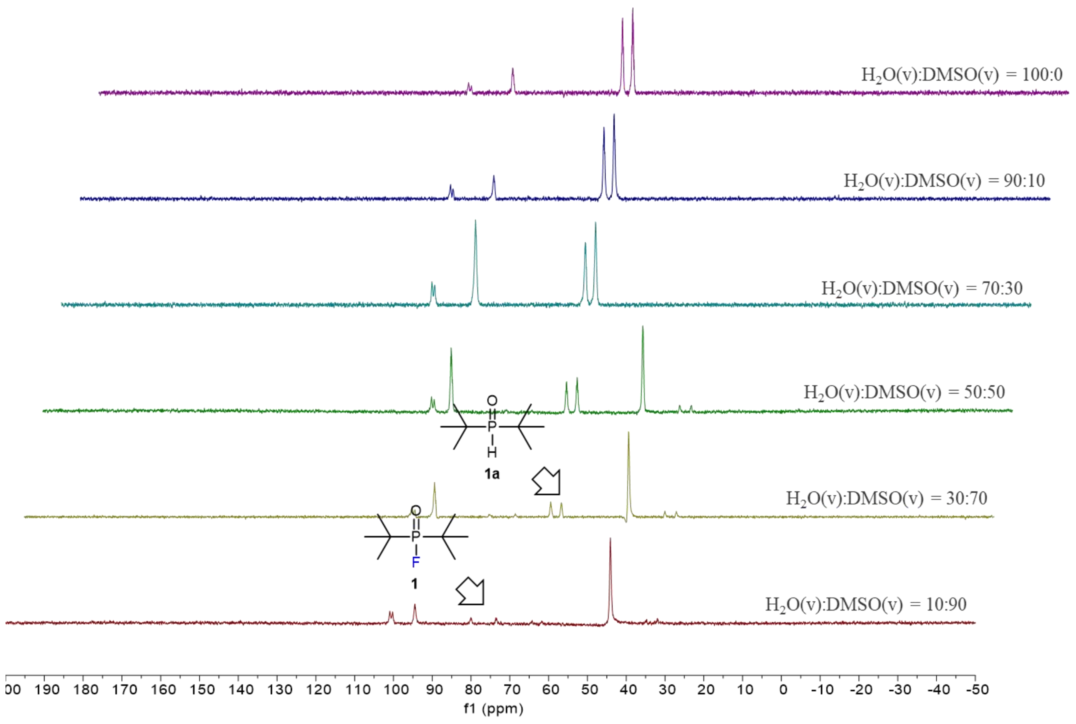

Figure S4. ^{31}P NMR analysis of substrate conversion on water addition study. Compound **1** ^{31}P NMR = 76.68 ppm (d); Compound **1a** ^{31}P NMR = 63.45 ppm (d). (a) ^{31}P NMR analysis on stoichiometric water addition; (b) ^{31}P NMR analysis on volumetric water addition.

3 Radiochemistry

3.1 [^{18}F]F$^-$ source

[^{18}F]F$^-$ was produced *via* the ^{18}O(p,n)^{18}F reaction by proton irradiation of an [^{18}O]H_2O containing target in the IBA 18/9 cyclotron. [^{18}F]F$^-$ in [^{18}O]H_2O was heated at 100 °C under a stream of nitrogen in a of glass vial to remove the solvent.

3.2 General procedure for ^{18}F-fluorination

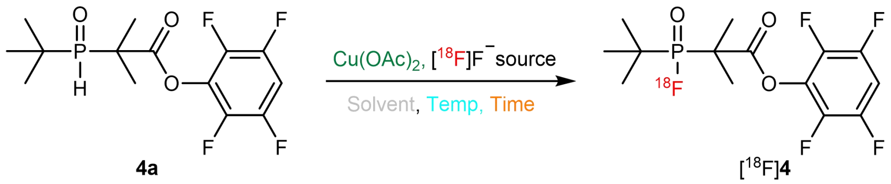

Scheme S3. General reaction routes of ^{18}F-labeling conditions.

Precursor **4a** (3 µmol) and $Cu(OAc)_2$ (2 equiv.) were dissolved in 50 µL acetone respectively and added to the glass vial with dried [^{18}F]F$^-$ source. The mixture was incubated at RT for 10 min. RCCs were analyzed by radio-TLC (n = 3).

3.3 Optimization of ^{18}F-labeling conditions

3.3.1 Relationship between RCCs and different solvents

Table S14. The effects of solvent on RCCs[a].

Entry	Solvent	RCCs (%)[b]	Average (%)
1	THF	30.0<br>23.3<br>24.5	25.9 ± 3.6 (n = 3)
2	Acetone	12.8<br>22.5<br>13.2	16.1 ± 5.5 (n = 3)
3	MeCN	12.3<br>11.0<br>15.1	12.8 ± 2.1 (n = 3)
4	DMF	23.3<br>22.6<br>26.0	24.0 ± 1.8 (n = 3)
5	DMSO	54.3<br>49.5<br>44.1	49.3 ± 5.1 (n = 3)

[a]Reaction condition: **4a** (3 µmol), $Cu(OAc)_2$ (2 equiv.), [^{18}F]TBAF, solvent (THF, Acetone, MeCN, DMF, DMSO), 25 °C, 10 min. [b]RCCs were determined by radio-TLC analysis.

3.3.2 Relationship between RCCs and reaction time

Table S15. The effects of reaction time on RCCs[a].

Entry	Reaction time (min)	RCCs (%)[b]	Average (%)
1	1	7.8<br>10.7<br>11.2	9.9 ± 1.8 (n = 3)
2	5	25.3<br>21.6<br>21.2	22.7 ± 2.2 (n = 3)
3	10	23.3<br>22.6<br>26.0	24.0 ± 1.8 (n = 3)
4	20	31.3<br>27.2<br>29.3	29.3 ± 2.0 (n = 3)
5	30	37.8<br>31.1<br>30.3	33.1 ± 4.1 (n = 3)

[a]Reaction condition: **4a** (3 μmol), $Cu(OAc)_2$ (2 equiv.), [^{18}F]TBAF, DMF, 25 °C, reaction time (1–30 min). [b]RCCs were determined by radio-TLC analysis.

3.3.3 Relationship between RCCs and precursor amount

Table S16. The effects of precursor amount on RCCs[a].

Entry	Precursor amount (μmol)	RCCs (%)[b]	Average (%)
1	0.1	0<br>0<br>1.2	0.4 ± 0.7 (n = 3)
2	0.5	16.8<br>16.2<br>11.8	14.9 ± 2.7 (n = 3)
3	1.0	13.2<br>20.6<br>26.0	19.9 ± 6.4 (n = 3)
4	3.0	54.3<br>49.5<br>44.1	49.3 ± 5.1 (n = 3)
5	5.0	20.9<br>29.0<br>19.2	23.1 ± 5.3 (n = 3)

[a]Reaction condition: **4a** (0.1–5.0 μmol), $Cu(OAc)_2$ (2 equiv.), [^{18}F]TBAF, DMSO, 25 °C, 10 min.
[b]RCCs were determined by radio-TLC analysis.

3.3.4 Relationship between RCCs and reaction temperature

Table S17. The effects of temperature on RCCs[a].

Entry	Solvent	Temperature (°C)	RCCs (%)[b]	Average (%)
1	Acetone	25	37.3<br>38.5<br>41.5	39.1 ± 2.2 (n = 3)
2	Acetone	50	13.2<br>9.0<br>10.5	10.9 ± 2.1 (n = 3)
3	DMSO	25	54.3<br>49.5<br>44.1	49.3 ± 5.1 (n = 3)
4	DMSO	50	42.9<br>35.0<br>45.1	41.0 ± 5.3 (n = 3)
5	DMSO	75	10.6<br>15.1	12.8 ± 3.2 (n = 2)
6	DMSO	100	14.4<br>10.5<br>11.4	12.1 ± 2.1 (n = 3)
7	DMSO	125	7.3<br>4.9<br>4.1	5.4 ± 1.7 (n = 3)

[a]Reaction condition: **4a** (3 μmol), $Cu(OAc)_2$ (2 equiv.), [^{18}F]TBAF, acetone or DMSO, 25–125 °C, 10 min. [b]RCCs were determined by radio-TLC analysis.

3.3.5 Relationship between RCCs and equivalent of $Cu(OAc)_2$

Table S18. The effects of equivalents of $Cu(OAc)_2$ on RCCs[a].

Entry	$Cu(OAc)_2$ (equiv.)	RCCs (%)[b]	Average (%)
1	0.0	5.2<br>0	2.6 ± 3.7 (n = 2)
2	0.5	21.8<br>10.4	16.1 ± 8.0 (n = 2)
3	1.0	28.8<br>27.9<br>24.8	27.2 ± 2.1 (n = 3)
4	2.0	54.3<br>49.5<br>44.1	49.3 ± 5.1 (n = 3)
5	4.0	14.1<br>19.9	17.0 ± 4.1 (n = 2)

[a]Reaction condition: **4a** (3.0 μmol), $Cu(OAc)_2$ (0.0–4.0 equiv.), [^{18}F]TBAF, DMSO, 25 °C, 10 min. [b]RCCs were determined by radio-TLC analysis.

3.3.6 Relationship between RCCs and ^{18}F-fluorine source

Table S19. The effects of ^{18}F-fluorine source on RCCs[a].

Entry	[^{18}F]F^- source	RCCs (%)[b]	Average (%)
1	[^{18}F]KF	31.1<br>29.3<br>32.6	31.0 ± 1.7 (n = 3)
2	[^{18}F]KF/K_{222}	22.6<br>28.4<br>26.3	25.8 ± 2.9 (n = 3)
3	[^{18}F]KF/18-c-6	39.6<br>46.4<br>44.6	43.5 ± 3.5 (n = 3)
4	[^{18}F]TBAF	54.3<br>49.5<br>44.1	49.3 ± 5.1 (n = 3)
5	[^{18}F]CsF	46.8<br>32.5<br>44.1	41.1 ± 7.6 (n = 3)

[a]Reaction condition: **4a** (3.0 μmol), $Cu(OAc)_2$ (2 equiv.), different [^{18}F]F^- source, DMSO, 25 °C, 10 min. [b]RCCs were determined by radio-TLC analysis.

3.3.7 Relationship between RCCs and water amount

Table S20. The effects of water amount on RCCs[a].

Entry	H_2O (equiv.)	RCCs (%)[b]	Average (%)
1	10	37.3<br>40.7<br>39.2	39.1 ± 1.7 (n = 3)
2	50	15.0<br>17.1<br>18.0	16.7 ± 1.5 (n = 3)
3	100	12.9<br>12.3<br>10.9	12.0 ± 1.0 (n = 3)
4	300	2.7<br>3.1<br>1.6	2.4 ± 0.7 (n = 3)
5	500	0<br>0<br>0	0 (n = 3)
6	1000	0<br>0<br>0	0 (n = 3)

[a]Reaction condition: **4a** (3.0 μmol), $Cu(OAc)_2$ (2 equiv.), [^{18}F]TBAF, H_2O (10–1000 equiv.), DMSO, 25 °C, 10 min. [b]RCCs were determined by radio-TLC analysis.

3.3.8 Characterization spectra by radio-TLC and radio-HPLC

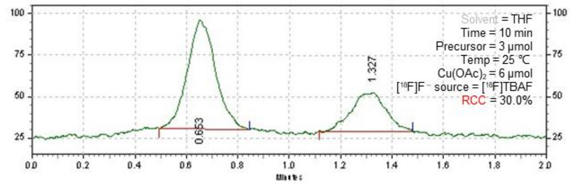

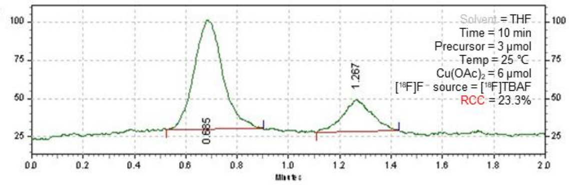

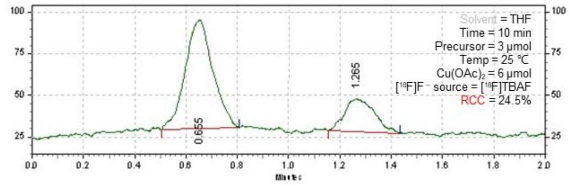

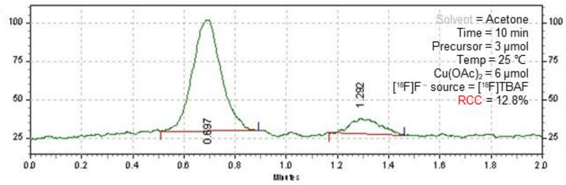

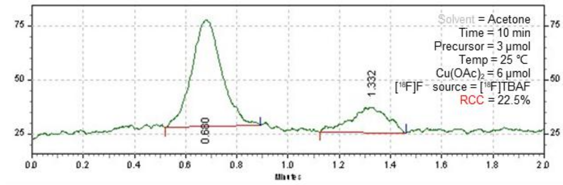

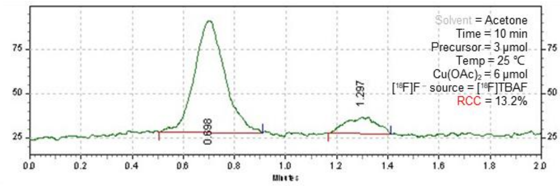

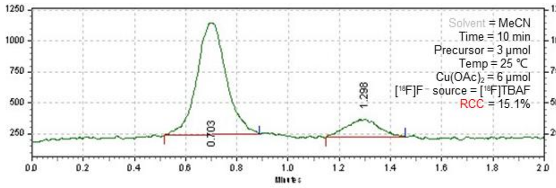

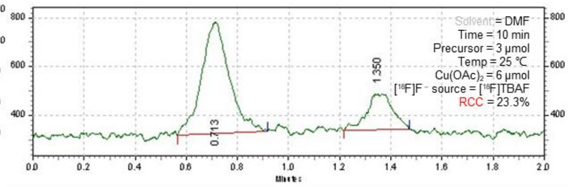

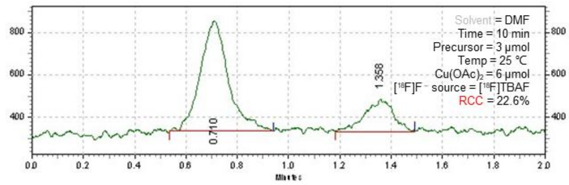

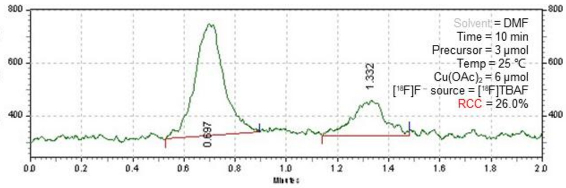

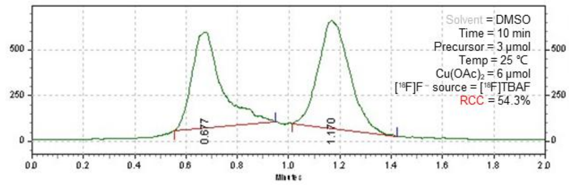

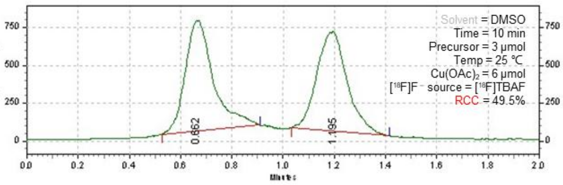

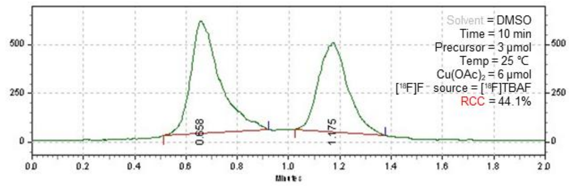

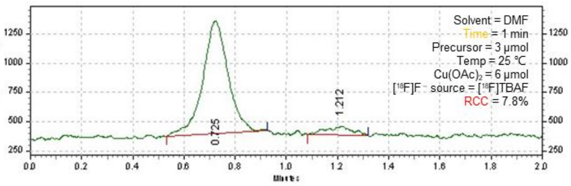

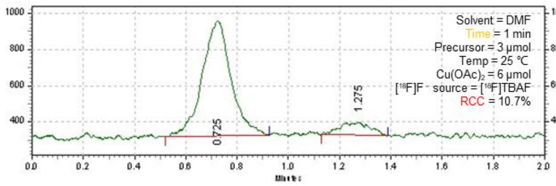

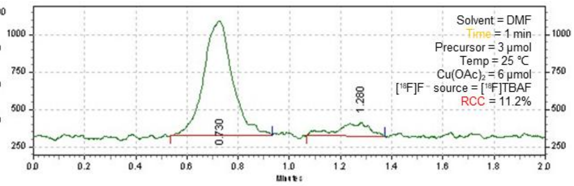

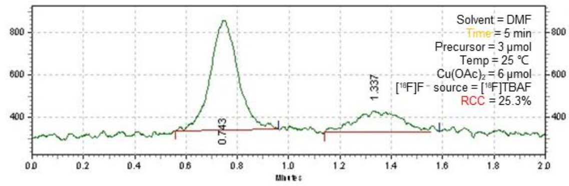
Solvent = DMF
Time = 5 min
Precursor = 3 μmol
Temp = 25 ℃
Cu(OAc)$_2$ = 6 μmol
[^{18}F]F$^-$ source = [^{18}F]TBAF
RCC = 25.3%

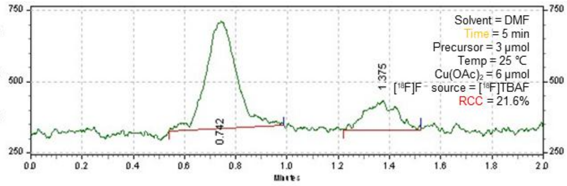
Solvent = DMF
Time = 5 min
Precursor = 3 μmol
Temp = 25 ℃
Cu(OAc)$_2$ = 6 μmol
[^{18}F]F$^-$ source = [^{18}F]TBAF
RCC = 21.6%

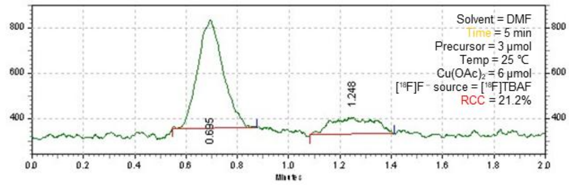
Solvent = DMF
Time = 5 min
Precursor = 3 μmol
Temp = 25 ℃
Cu(OAc)$_2$ = 6 μmol
[^{18}F]F$^-$ source = [^{18}F]TBAF
RCC = 21.2%

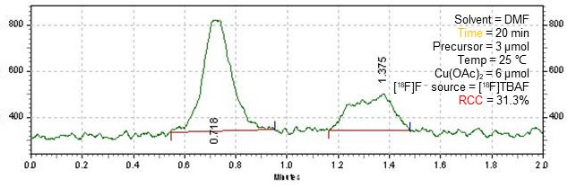
Solvent = DMF
Time = 20 min
Precursor = 3 μmol
Temp = 25 ℃
Cu(OAc)$_2$ = 6 μmol
[^{18}F]F$^-$ source = [^{18}F]TBAF
RCC = 31.3%

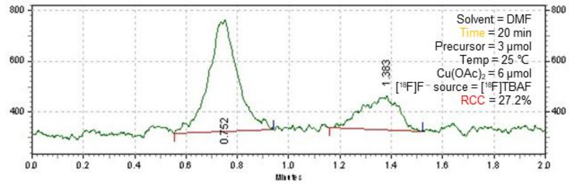
Solvent = DMF
Time = 20 min
Precursor = 3 μmol
Temp = 25 ℃
Cu(OAc)$_2$ = 6 μmol
[^{18}F]F$^-$ source = [^{18}F]TBAF
RCC = 27.2%

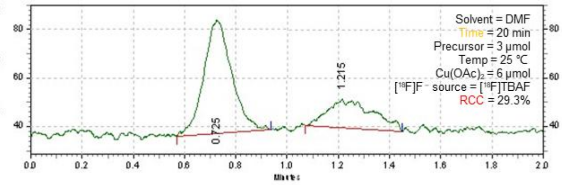
Solvent = DMF
Time = 20 min
Precursor = 3 μmol
Temp = 25 ℃
Cu(OAc)$_2$ = 6 μmol
[^{18}F]F$^-$ source = [^{18}F]TBAF
RCC = 29.3%

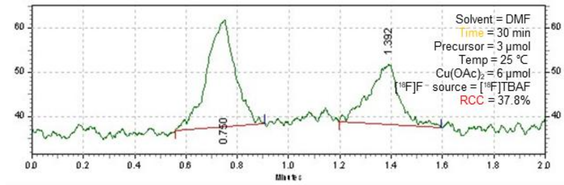
Solvent = DMF
Time = 30 min
Precursor = 3 μmol
Temp = 25 ℃
Cu(OAc)$_2$ = 6 μmol
[^{18}F]F$^-$ source = [^{18}F]TBAF
RCC = 37.8%

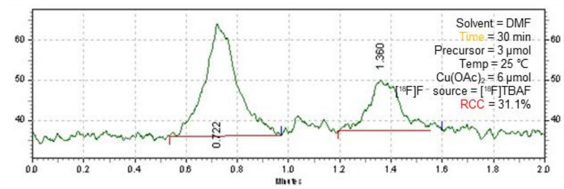
Solvent = DMF
Time = 30 min
Precursor = 3 μmol
Temp = 25 ℃
Cu(OAc)$_2$ = 6 μmol
[^{18}F]F$^-$ source = [^{18}F]TBAF
RCC = 31.1%

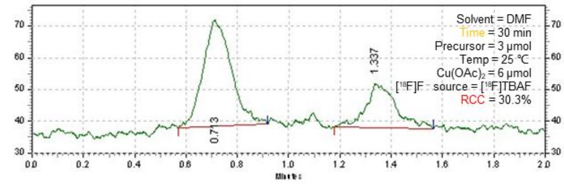
Solvent = DMF
Time = 30 min
Precursor = 3 μmol
Temp = 25 ℃
Cu(OAc)$_2$ = 6 μmol
[^{18}F]F$^-$ source = [^{18}F]TBAF
RCC = 30.3%

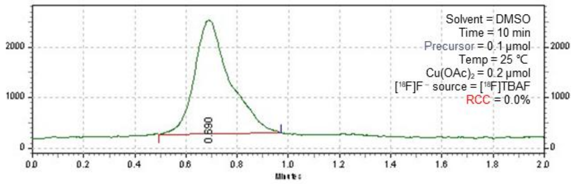
Solvent = DMSO
Time = 10 min
Precursor = 0.1 μmol
Temp = 25 ℃
Cu(OAc)$_2$ = 0.2 μmol
[^{18}F]F$^-$ source = [^{18}F]TBAF
RCC = 0.0%

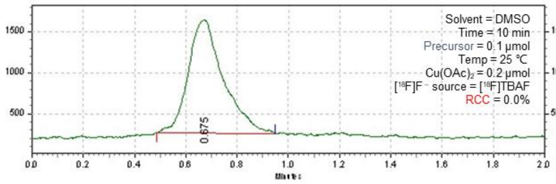
Solvent = DMSO
Time = 10 min
Precursor = 0.1 μmol
Temp = 25 ℃
Cu(OAc)$_2$ = 0.2 μmol
[^{18}F]F$^-$ source = [^{18}F]TBAF
RCC = 0.0%

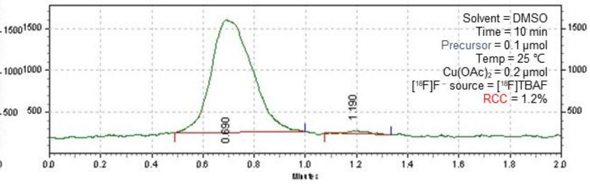
Solvent = DMSO
Time = 10 min
Precursor = 0.1 μmol
Temp = 25 ℃
Cu(OAc)$_2$ = 0.2 μmol
[^{18}F]F$^-$ source = [^{18}F]TBAF
RCC = 1.2%

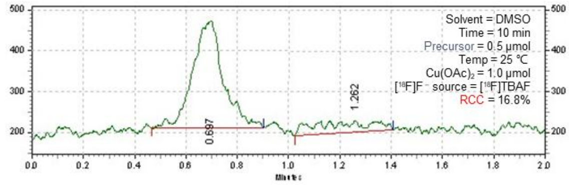
Solvent = DMSO
Time = 10 min
Precursor = 0.5 μmol
Temp = 25 ℃
Cu(OAc)$_2$ = 1.0 μmol
[^{18}F]F$^-$ source = [^{18}F]TBAF
RCC = 16.8%

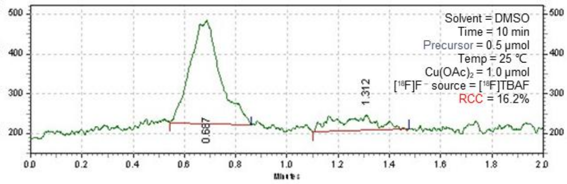
Solvent = DMSO
Time = 10 min
Precursor = 0.5 μmol
Temp = 25 ℃
Cu(OAc)$_2$ = 1.0 μmol
[^{18}F]F$^-$ source = [^{18}F]TBAF
RCC = 16.2%

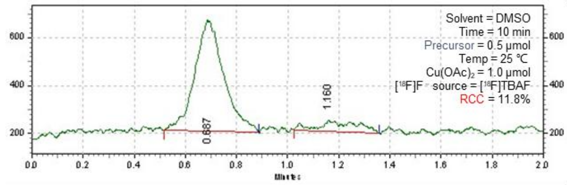
Solvent = DMSO
Time = 10 min
Precursor = 0.5 μmol
Temp = 25 ℃
Cu(OAc)$_2$ = 1.0 μmol
[^{18}F]F$^-$ source = [^{18}F]TBAF
RCC = 11.8%

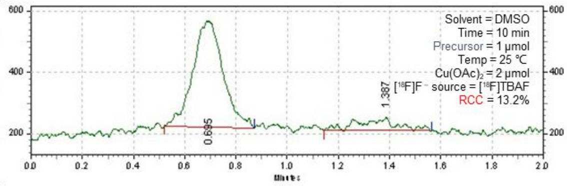
Solvent = DMSO
Time = 10 min
Precursor = 1 μmol
Temp = 25 ℃
Cu(OAc)$_2$ = 2 μmol
[^{18}F]F$^-$ source = [^{18}F]TBAF
RCC = 13.2%

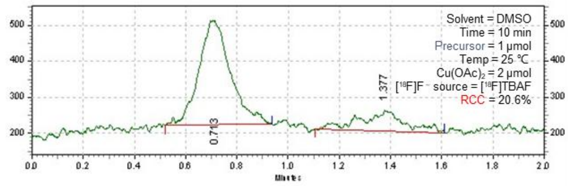
Solvent = DMSO
Time = 10 min
Precursor = 1 μmol
Temp = 25 ℃
Cu(OAc)$_2$ = 2 μmol
[^{18}F]F$^-$ source = [^{18}F]TBAF
RCC = 20.6%

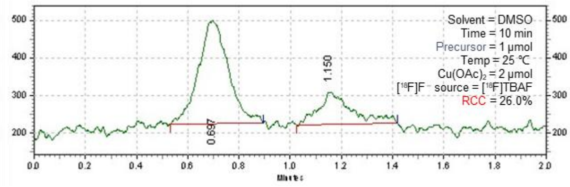
Solvent = DMSO
Time = 10 min
Precursor = 1 μmol
Temp = 25 ℃
Cu(OAc)$_2$ = 2 μmol
[^{18}F]F$^-$ source = [^{18}F]TBAF
RCC = 26.0%

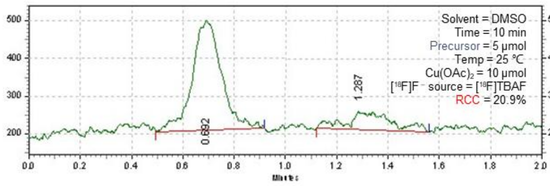
Solvent = DMSO
Time = 10 min
Precursor = 5 μmol
Temp = 25 ℃
$Cu(OAc)_2$ = 10 μmol
$[^{18}F]F^-$ source = $[^{18}F]$TBAF
RCC = 20.9%

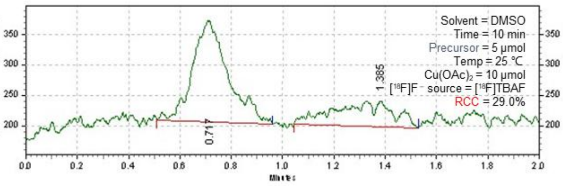
Solvent = DMSO
Time = 10 min
Precursor = 5 μmol
Temp = 25 ℃
$Cu(OAc)_2$ = 10 μmol
$[^{18}F]F^-$ source = $[^{18}F]$TBAF
RCC = 29.0%

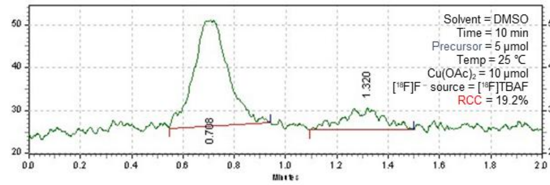
Solvent = DMSO
Time = 10 min
Precursor = 5 μmol
Temp = 25 ℃
$Cu(OAc)_2$ = 10 μmol
$[^{18}F]F^-$ source = $[^{18}F]$TBAF
RCC = 19.2%

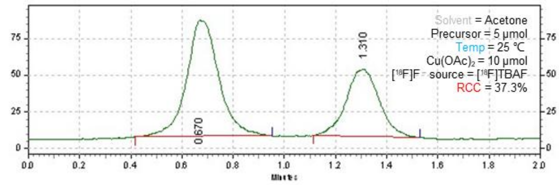
Solvent = Acetone
Precursor = 5 μmol
Temp = 25 ℃
$Cu(OAc)_2$ = 10 μmol
$[^{18}F]F^-$ source = $[^{18}F]$TBAF
RCC = 37.3%

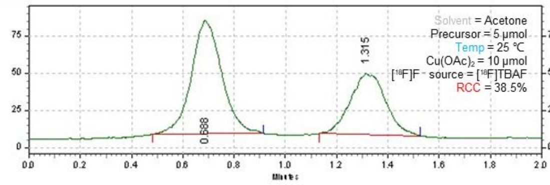
Solvent = Acetone
Precursor = 5 μmol
Temp = 25 ℃
$Cu(OAc)_2$ = 10 μmol
$[^{18}F]F^-$ source = $[^{18}F]$TBAF
RCC = 38.5%

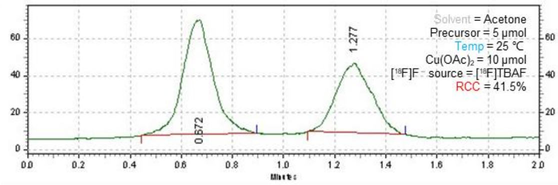
Solvent = Acetone
Precursor = 5 μmol
Temp = 25 ℃
$Cu(OAc)_2$ = 10 μmol
$[^{18}F]F^-$ source = $[^{18}F]$TBAF
RCC = 41.5%

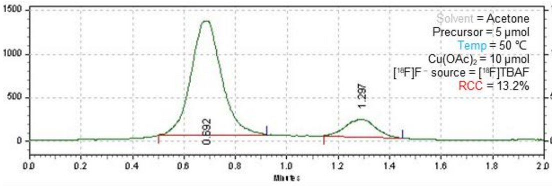
Solvent = Acetone
Precursor = 5 μmol
Temp = 50 ℃
$Cu(OAc)_2$ = 10 μmol
$[^{18}F]F^-$ source = $[^{18}F]$TBAF
RCC = 13.2%

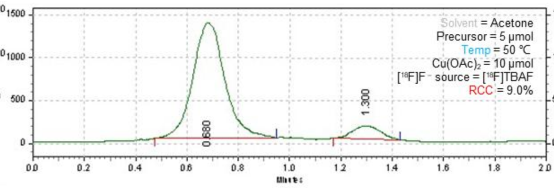
Solvent = Acetone
Precursor = 5 μmol
Temp = 50 ℃
$Cu(OAc)_2$ = 10 μmol
$[^{18}F]F^-$ source = $[^{18}F]$TBAF
RCC = 9.0%

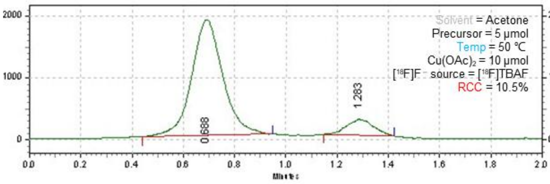
Solvent = Acetone
Precursor = 5 μmol
Temp = 50 ℃
$Cu(OAc)_2$ = 10 μmol
$[^{18}F]F^-$ source = $[^{18}F]$TBAF
RCC = 10.5%

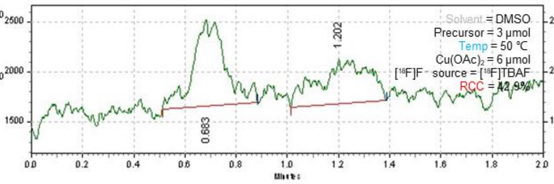
Solvent = DMSO
Precursor = 3 μmol
Temp = 50 ℃
$Cu(OAc)_2$ = 6 μmol
$[^{18}F]F^-$ source = $[^{18}F]$TBAF
RCC = 42.9%

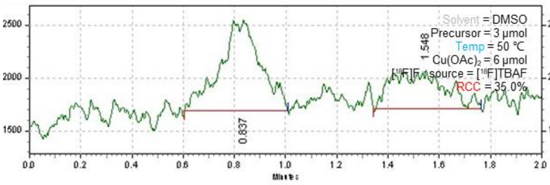
Solvent = DMSO
Precursor = 3 μmol
Temp = 50 ℃
$Cu(OAc)_2$ = 6 μmol
$[^{18}F]F^-$ source = $[^{18}F]$TBAF
RCC = 35.0%

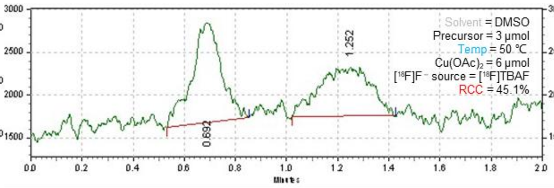
Solvent = DMSO
Precursor = 3 μmol
Temp = 50 ℃
$Cu(OAc)_2$ = 6 μmol
$[^{18}F]F^-$ source = $[^{18}F]$TBAF
RCC = 45.1%

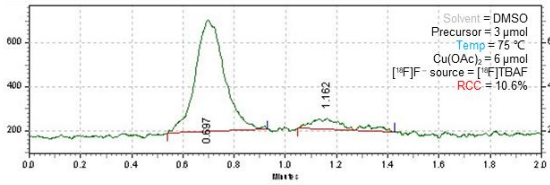
Solvent = DMSO
Precursor = 3 μmol
Temp = 75 ℃
$Cu(OAc)_2$ = 6 μmol
$[^{18}F]F^-$ source = $[^{18}F]$TBAF
RCC = 10.6%

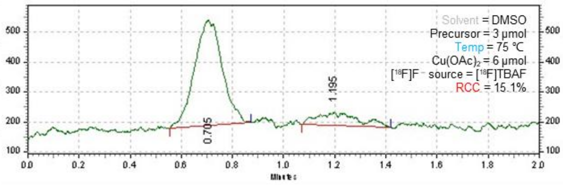
Solvent = DMSO
Precursor = 3 μmol
Temp = 75 ℃
$Cu(OAc)_2$ = 6 μmol
$[^{18}F]F^-$ source = $[^{18}F]$TBAF
RCC = 15.1%

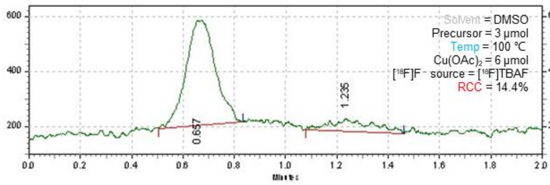
Solvent = DMSO
Precursor = 3 μmol
Temp = 100 ℃
$Cu(OAc)_2$ = 6 μmol
$[^{18}F]F^-$ source = $[^{18}F]$TBAF
RCC = 14.4%

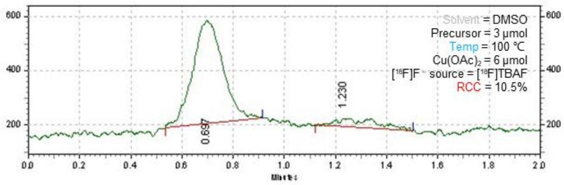
Solvent = DMSO
Precursor = 3 μmol
Temp = 100 ℃
$Cu(OAc)_2$ = 6 μmol
$[^{18}F]F^-$ source = $[^{18}F]$TBAF
RCC = 10.5%

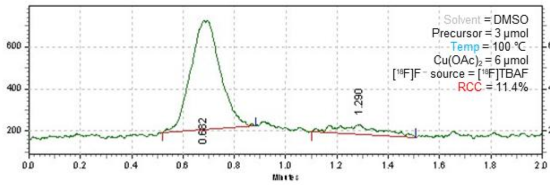
Solvent = DMSO
Precursor = 3 μmol
Temp = 100 ℃
$Cu(OAc)_2$ = 6 μmol
$[^{18}F]F^-$ source = $[^{18}F]$TBAF
RCC = 11.4%

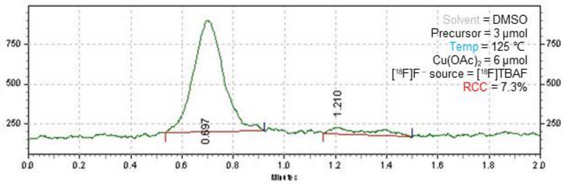
Solvent = DMSO
Precursor = 3 μmol
Temp = 125 ℃
$Cu(OAc)_2$ = 6 μmol
$[^{18}F]F^-$ source = $[^{18}F]$TBAF
RCC = 7.3%

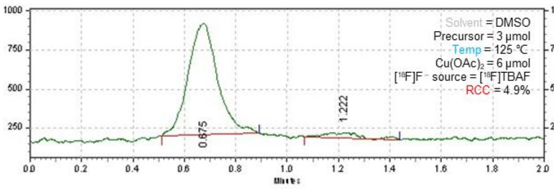
Solvent = DMSO
Precursor = 3 μmol
Temp = 125 ℃
$Cu(OAc)_2$ = 6 μmol
[^{18}F]F⁻ source = [^{18}F]TBAF
RCC = 4.9%

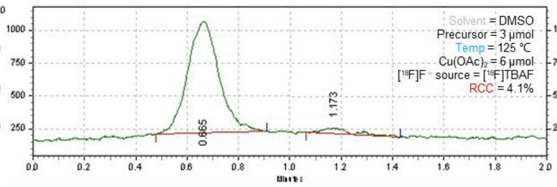
Solvent = DMSO
Precursor = 3 μmol
Temp = 125 ℃
$Cu(OAc)_2$ = 6 μmol
[^{18}F]F⁻ source = [^{18}F]TBAF
RCC = 4.1%

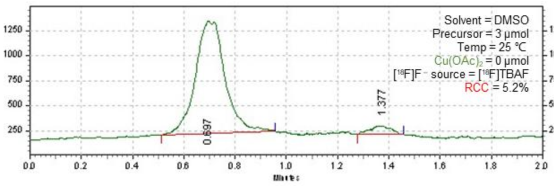
Solvent = DMSO
Precursor = 3 μmol
Temp = 25 ℃
$Cu(OAc)_2$ = 0 μmol
[^{18}F]F⁻ source = [^{18}F]TBAF
RCC = 5.2%

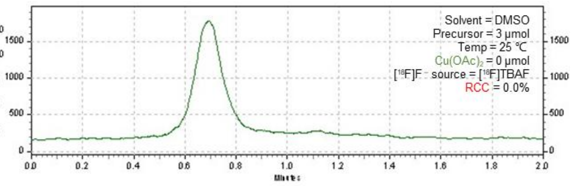
Solvent = DMSO
Precursor = 3 μmol
Temp = 25 ℃
$Cu(OAc)_2$ = 0 μmol
[^{18}F]F⁻ source = [^{18}F]TBAF
RCC = 0.0%

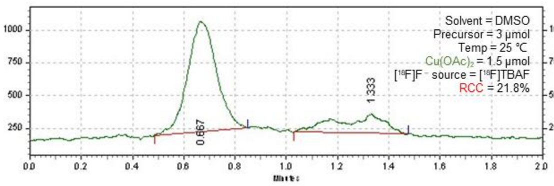
Solvent = DMSO
Precursor = 3 μmol
Temp = 25 ℃
$Cu(OAc)_2$ = 1.5 μmol
[^{18}F]F⁻ source = [^{18}F]TBAF
RCC = 21.8%

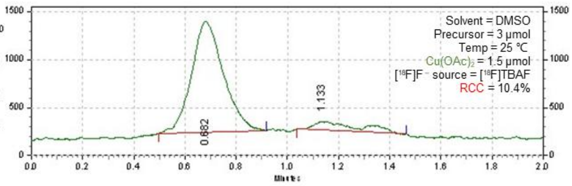
Solvent = DMSO
Precursor = 3 μmol
Temp = 25 ℃
$Cu(OAc)_2$ = 1.5 μmol
[^{18}F]F⁻ source = [^{18}F]TBAF
RCC = 10.4%

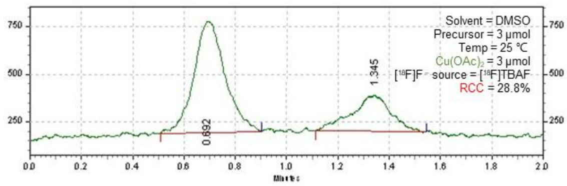
Solvent = DMSO
Precursor = 3 μmol
Temp = 25 ℃
$Cu(OAc)_2$ = 3 μmol
[^{18}F]F⁻ source = [^{18}F]TBAF
RCC = 28.8%

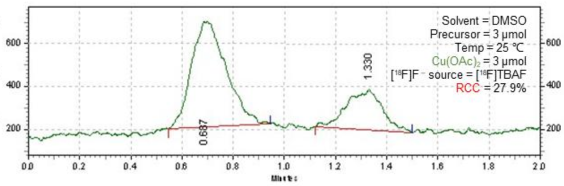
Solvent = DMSO
Precursor = 3 μmol
Temp = 25 ℃
$Cu(OAc)_2$ = 3 μmol
[^{18}F]F⁻ source = [^{18}F]TBAF
RCC = 27.9%

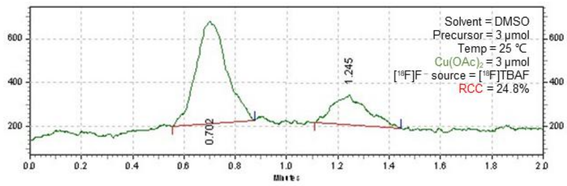
Solvent = DMSO
Precursor = 3 μmol
Temp = 25 ℃
$Cu(OAc)_2$ = 3 μmol
[^{18}F]F⁻ source = [^{18}F]TBAF
RCC = 24.8%

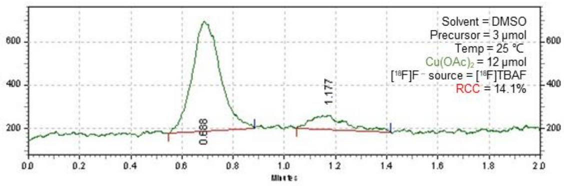
Solvent = DMSO
Precursor = 3 μmol
Temp = 25 ℃
$Cu(OAc)_2$ = 12 μmol
[^{18}F]F⁻ source = [^{18}F]TBAF
RCC = 14.1%

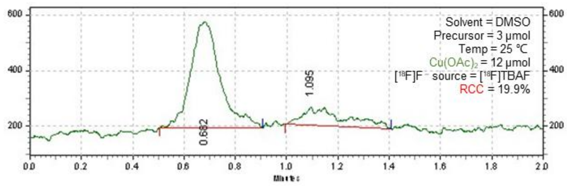
Solvent = DMSO
Precursor = 3 μmol
Temp = 25 ℃
$Cu(OAc)_2$ = 12 μmol
[^{18}F]F⁻ source = [^{18}F]TBAF
RCC = 19.9%

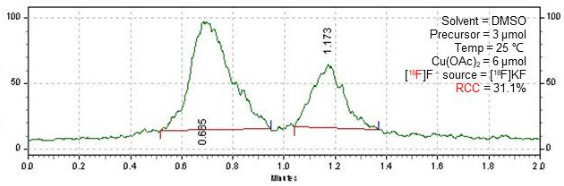
Solvent = DMSO
Precursor = 3 μmol
Temp = 25 ℃
$Cu(OAc)_2$ = 6 μmol
[^{18}F]F⁻ source = [^{18}F]KF
RCC = 31.1%

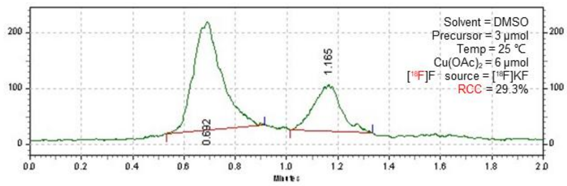
Solvent = DMSO
Precursor = 3 μmol
Temp = 25 ℃
$Cu(OAc)_2$ = 6 μmol
[^{18}F]F⁻ source = [^{18}F]KF
RCC = 29.3%

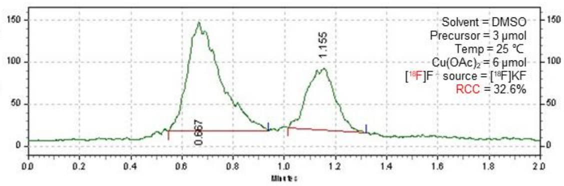
Solvent = DMSO
Precursor = 3 μmol
Temp = 25 ℃
$Cu(OAc)_2$ = 6 μmol
[^{18}F]F⁻ source = [^{18}F]KF
RCC = 32.6%

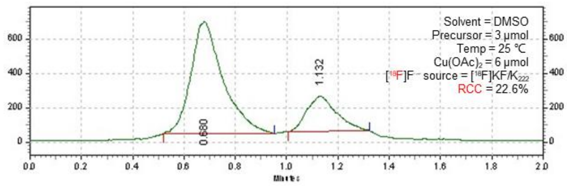
Solvent = DMSO
Precursor = 3 μmol
Temp = 25 ℃
$Cu(OAc)_2$ = 6 μmol
[^{18}F]F⁻ source = [^{18}F]KF/K_{222}
RCC = 22.6%

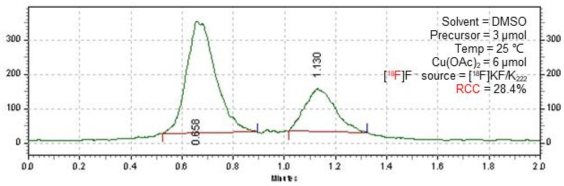
Solvent = DMSO
Precursor = 3 μmol
Temp = 25 ℃
$Cu(OAc)_2$ = 6 μmol
[^{18}F]F⁻ source = [^{18}F]KF/K_{222}
RCC = 28.4%

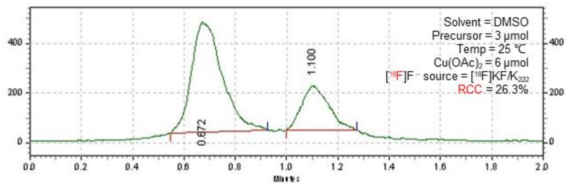
Solvent = DMSO
Precursor = 3 μmol
Temp = 25 ℃
$Cu(OAc)_2$ = 6 μmol
[^{18}F]F⁻ source = [^{18}F]KF/K_{222}
RCC = 26.3%

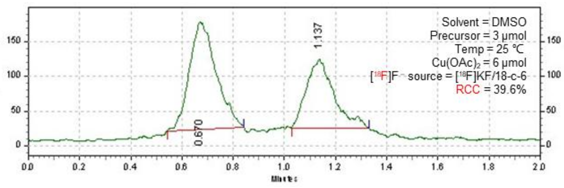
Solvent = DMSO
Precursor = 3 μmol
Temp = 25 ℃
$Cu(OAc)_2$ = 6 μmol
[^{18}F]F⁻ source = [^{18}F]KF/18-c-6
RCC = 39.6%

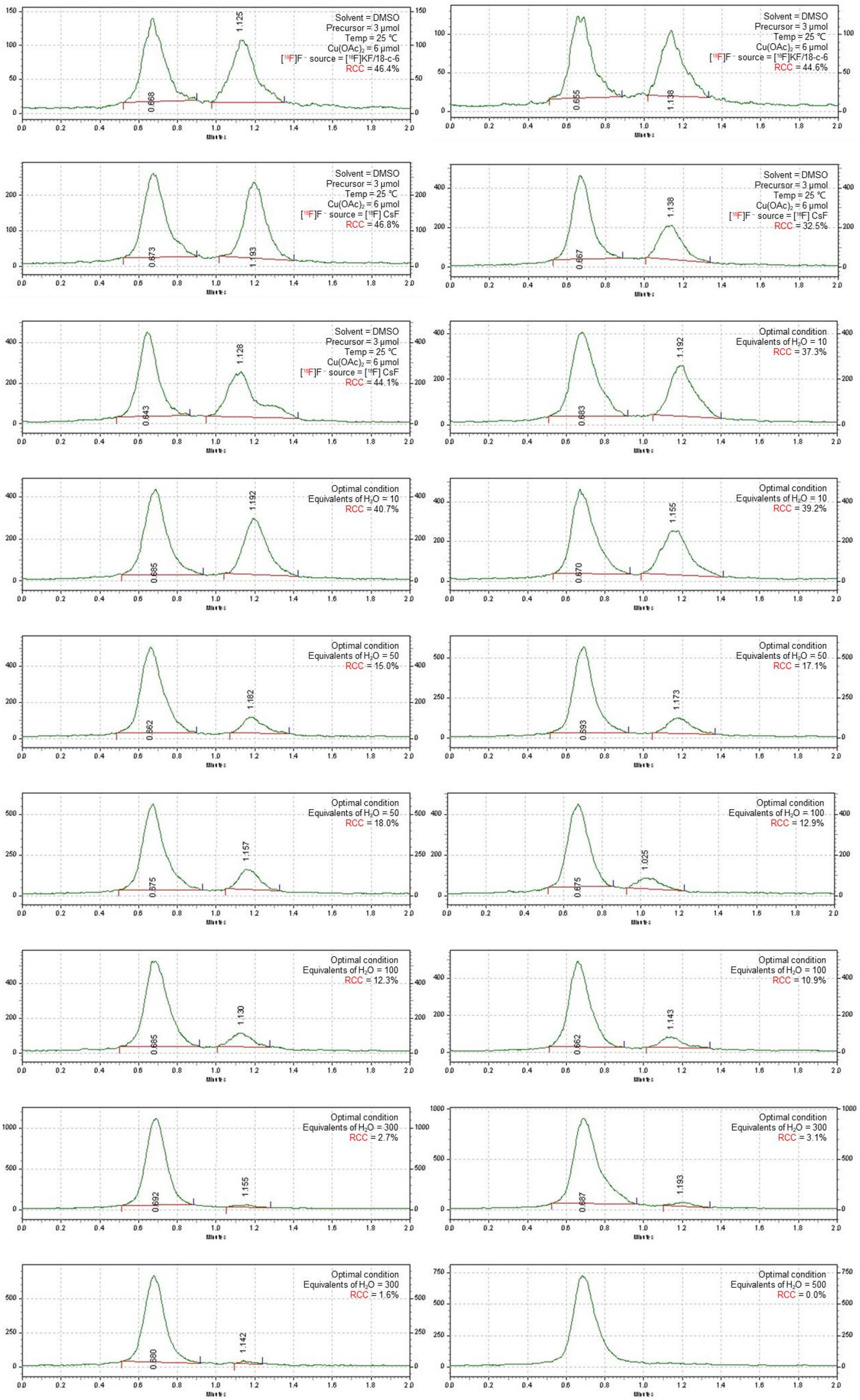
Solvent = DMSO
Precursor = 3 μmol
Temp = 25 ℃
$Cu(OAc)_2$ = 6 μmol
[^{18}F]F$^-$ source = [^{18}F]KF/18-c-6
RCC = 46.4%
Solvent = DMSO
Precursor = 3 μmol
Temp = 25 ℃
$Cu(OAc)_2$ = 6 μmol
[^{18}F]F$^-$ source = [^{18}F]KF/18-c-6
RCC = 44.6%
Solvent = DMSO
Precursor = 3 μmol
Temp = 25 ℃
$Cu(OAc)_2$ = 6 μmol
[^{18}F]F$^-$ source = [^{18}F] CsF
RCC = 46.8%
Solvent = DMSO
Precursor = 3 μmol
Temp = 25 ℃
$Cu(OAc)_2$ = 6 μmol
[^{18}F]F$^-$ source = [^{18}F] CsF
RCC = 32.5%
Solvent = DMSO
Precursor = 3 μmol
Temp = 25 ℃
$Cu(OAc)_2$ = 6 μmol
[^{18}F]F$^-$ source = [^{18}F] CsF
RCC = 44.1%
Optimal condition
Equivalents of H_2O = 10
RCC = 37.3%
Optimal condition
Equivalents of H_2O = 10
RCC = 40.7%
Optimal condition
Equivalents of H_2O = 10
RCC = 39.2%
Optimal condition
Equivalents of H_2O = 50
RCC = 15.0%
Optimal condition
Equivalents of H_2O = 50
RCC = 17.1%
Optimal condition
Equivalents of H_2O = 50
RCC = 18.0%
Optimal condition
Equivalents of H_2O = 100
RCC = 12.9%
Optimal condition
Equivalents of H_2O = 100
RCC = 12.3%
Optimal condition
Equivalents of H_2O = 100
RCC = 10.9%
Optimal condition
Equivalents of H_2O = 300
RCC = 2.7%
Optimal condition
Equivalents of H_2O = 300
RCC = 3.1%
Optimal condition
Equivalents of H_2O = 300
RCC = 1.6%
Optimal condition
Equivalents of H_2O = 500
RCC = 0.0%

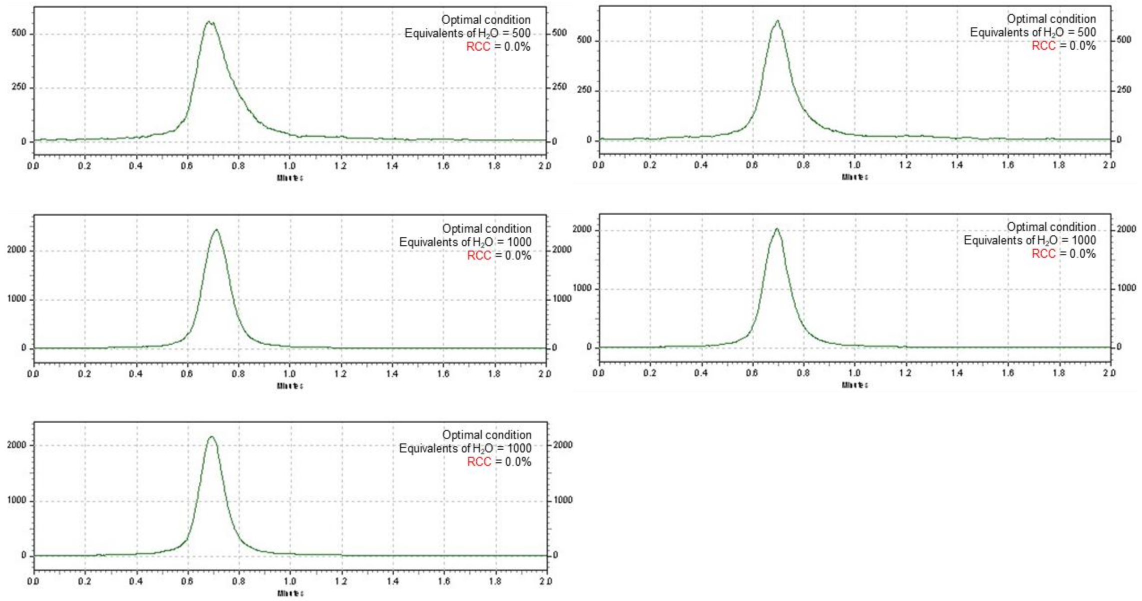

Figure S5. Radio-TLC analysis of RCCs on ^{18}F-labeling conditions of [^{18}F]**4**.

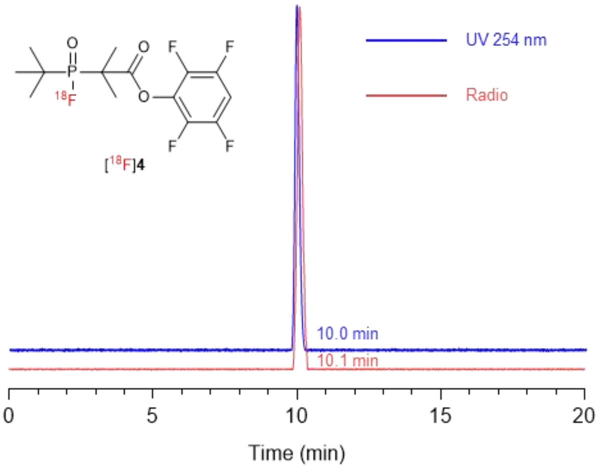

Figure S6. Radio-HPLC analysis of co-injection with cold standard for [^{18}F]**4** confirmation. HPLC condition: Column: Thermo Scientific™ Acclaim™ 120 C18 (250 × 4.6 mm, 5 μm). Solvent A: MeCN, Solvent B: H_2O, isocrat: 0 to 20 min: isocratic elution at 40% solvent A and 60% solvent B. Flow rate: 1 mL/min. Column temperature: 25 ℃.

3.4 UV standard curve of 4

Table S21. UV absorption peak areas corresponding to different amounts of compound **4**.

n (nmol)	0.011389	0.056945	0.113890	0.569450	1.138900	5.694500
Area (mAu*min)	0.0100	0.0360	0.0667	0.3678	0.6686	3.3296

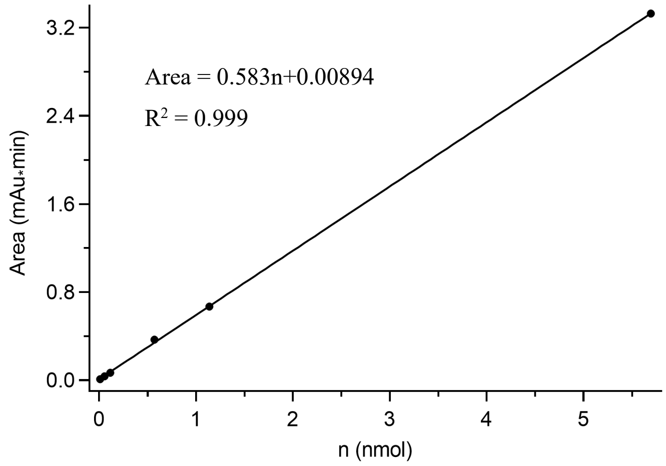

Figure S7. UV standard curve of compound **4**.

3.5 ^{18}F-Labeling of [^{18}F]1-[^{18}F]3

3.5.1 ^{18}F-Labeling of [^{18}F]1

Table S22. ^{18}F-labeling RCCs of [^{18}F]**1**[a].

Precursor	RCC_{TLC} (%)[b]	RCC_{HPLC} (%)[b]
**1a**	0 (n = 3)	0 (n = 1)

[a]Reaction condition: **1a** (1 equiv.), $Cu(OAc)_2$ (2 equiv.), [^{18}F]TBAF, DMSO, 25 °C, 10 min. [b]RCCs were determined by radio-TLC and radio-HPLC analysis.

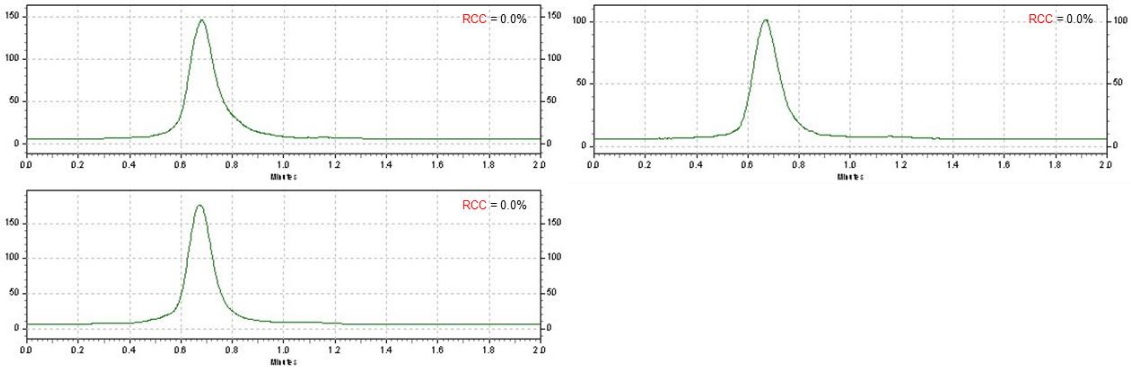

Figure S8. Radio-TLC analysis of RCCs on [^{18}F]**1**.

3.5.2 ^{18}F-Labeling of [^{18}F]2

Table S23. ^{18}F-labeling RCCs of [^{18}F]**2**^{a}.

Precursor	RCC_{TLC} (%)^b	RCC_{HPLC} (%)^b
**2a**	0 (n = 3)	0 (n = 1)

^{a}Reaction condition: **2a** (1 equiv.), $Cu(OAc)_2$ (2 equiv.), [^{18}F]TBAF, DMSO, 25 °C, 10 min. ^{b}RCCs were determined by radio-TLC and radio-HPLC analysis.

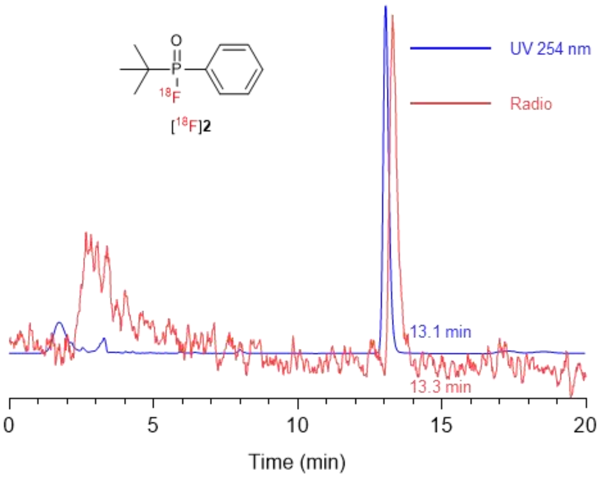

Figure S9. Radio-HPLC analysis of co-injection with cold standard for [^{18}F]**2** confirmation. HPLC condition: Column: Thermo Scientific™ Acclaim™ 120 C18 (250 × 4.6 mm, 5 µm). Solvent A: MeCN, Solvent B: H_2O, isocrat: 0 to 20 min: isocratic elution at 35% solvent A and 65% solvent B. Flow rate: 1 mL/min. Column temperature: 25 °C.

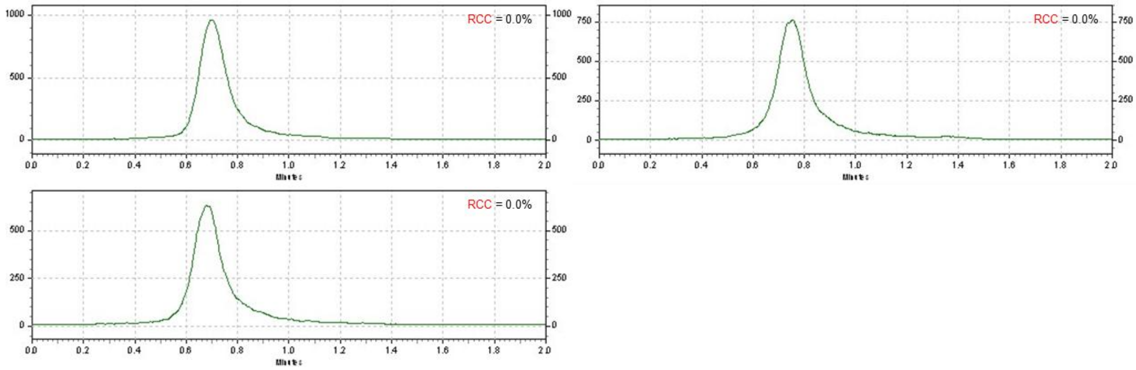

Figure S10. Radio-TLC analysis of RCCs on ^{18}F-labeling [^{18}F]**2**.

3.5.3 ^{18}F-Labeling of [^{18}F]3

Table S24. ^{18}F-labeling RCCs of [^{18}F]**3**[a].

Precursor	RCC_{TLC} (%)[b]	RCC_{HPLC} (%)[b]
**3a**	7.2 ± 3.5 (n = 3)	29.3 (n = 1)

[a]Reaction condition: **3a** (1 equiv.), $Cu(OAc)_2$ (2 equiv.), [^{18}F]TBAF, DMSO, 25 °C, 10 min. [b]RCCs were determined by radio-TLC and radio-HPLC analysis.

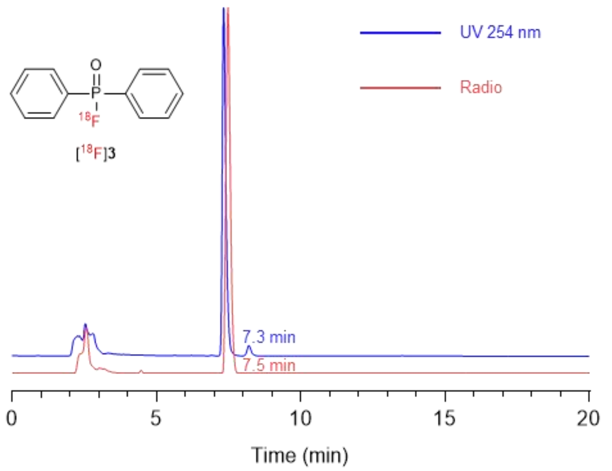

Figure S11. Radio-HPLC analysis of co-injection with cold standard for [^{18}F]**3** confirmation. HPLC condition: Column: Thermo Scientific™ Acclaim™ 120 C18 (250 × 4.6 mm, 5 μm). Solvent A: MeCN, Solvent B: H_2O, isocrat: 0 to 20 min: isocratic elution at 50% solvent A and 50% solvent B. Flow rate: 1 mL/min. Column temperature: 25 °C.

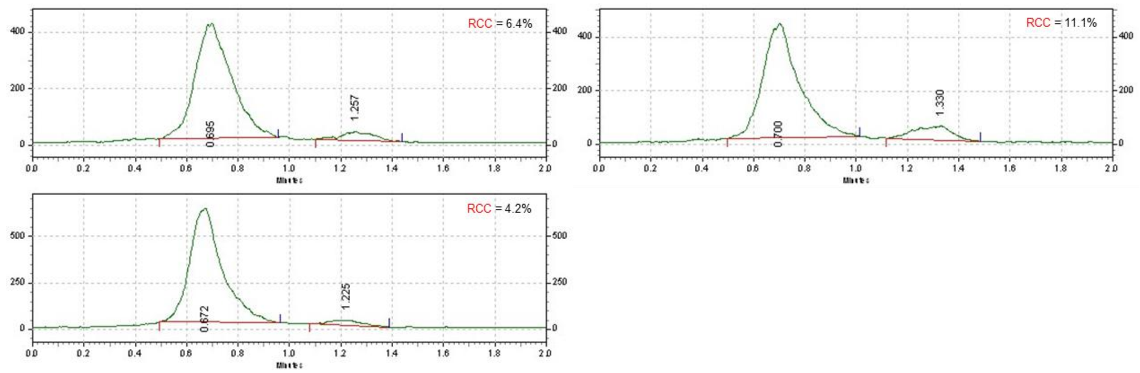

Figure S12. Radio-TLC analysis of RCCs on ^{18}F-labeling [^{18}F]**3**.

4 Appendix (^{1}H, ^{13}C, ^{19}F, ^{31}P NMR and MS spectra)

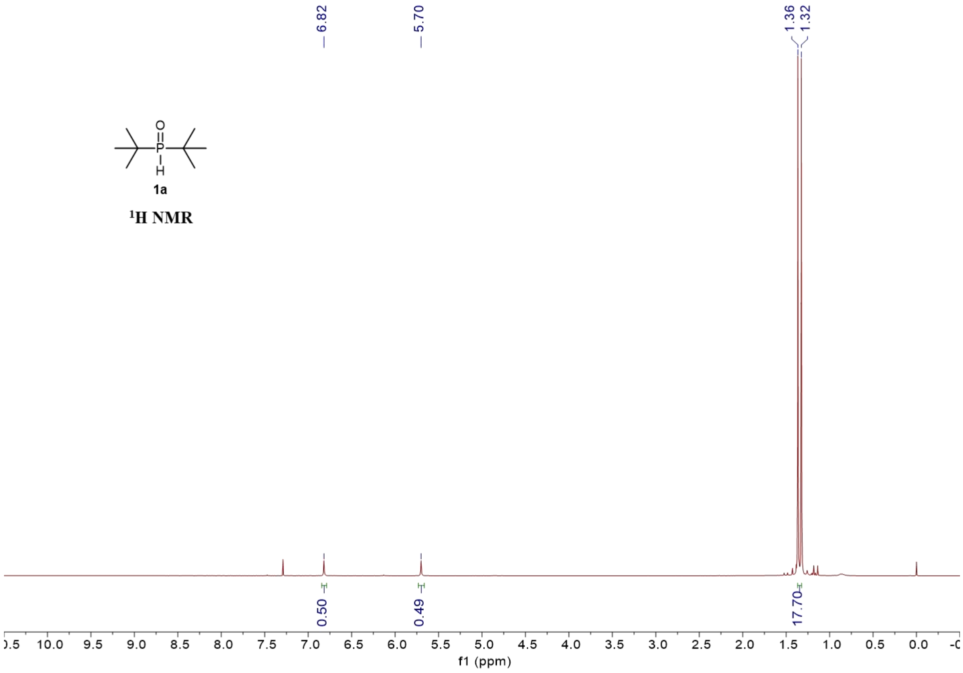

Figure S13. ^{1}H NMR spectrum of compound **1a**.

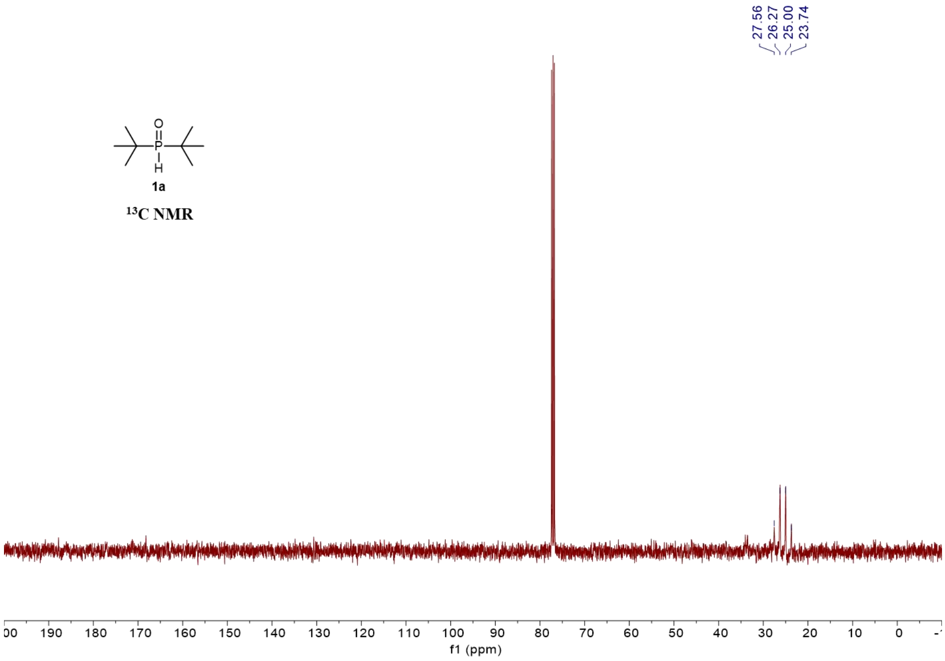

Figure S14. ^{13}C NMR spectrum of compound **1a**.

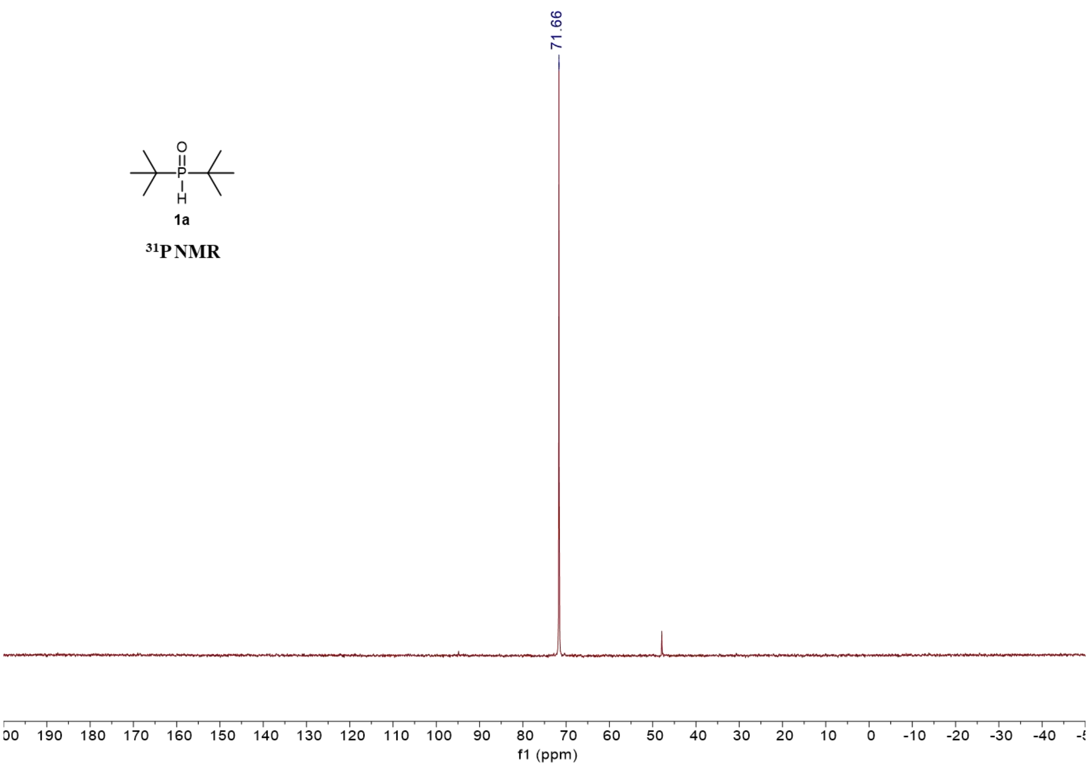

Figure S15. ^{31}P NMR spectrum of compound **1a**.

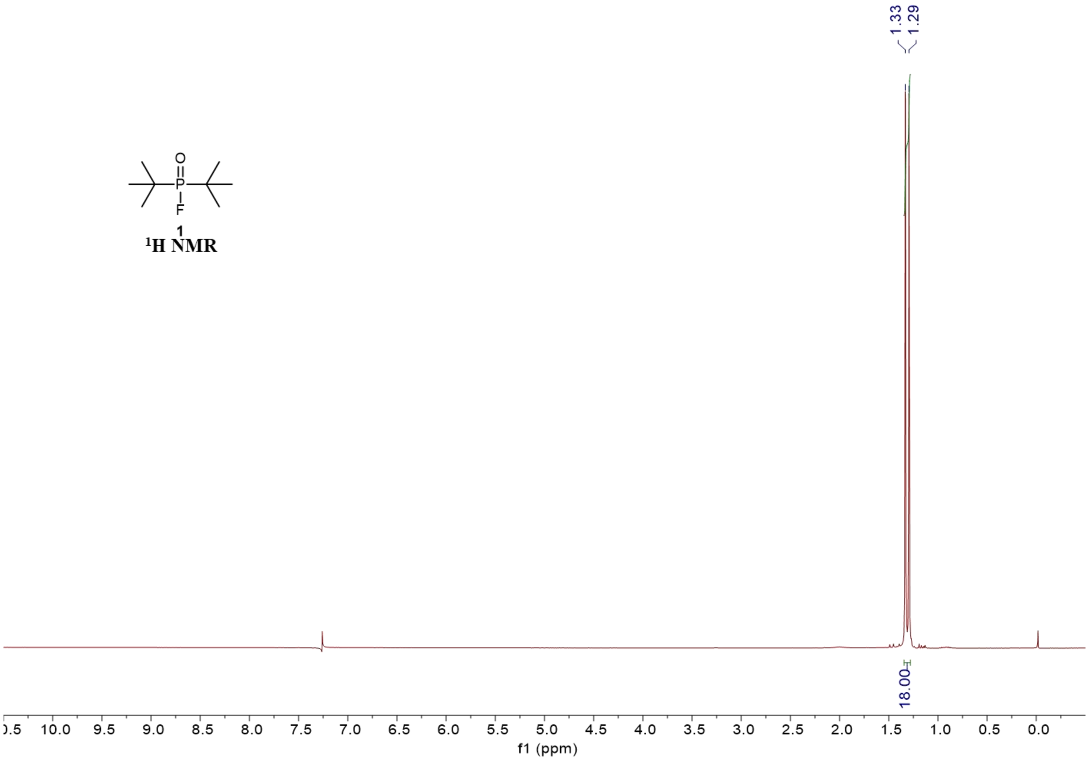

Figure S16. ^{1}H NMR spectrum of compound **1**.

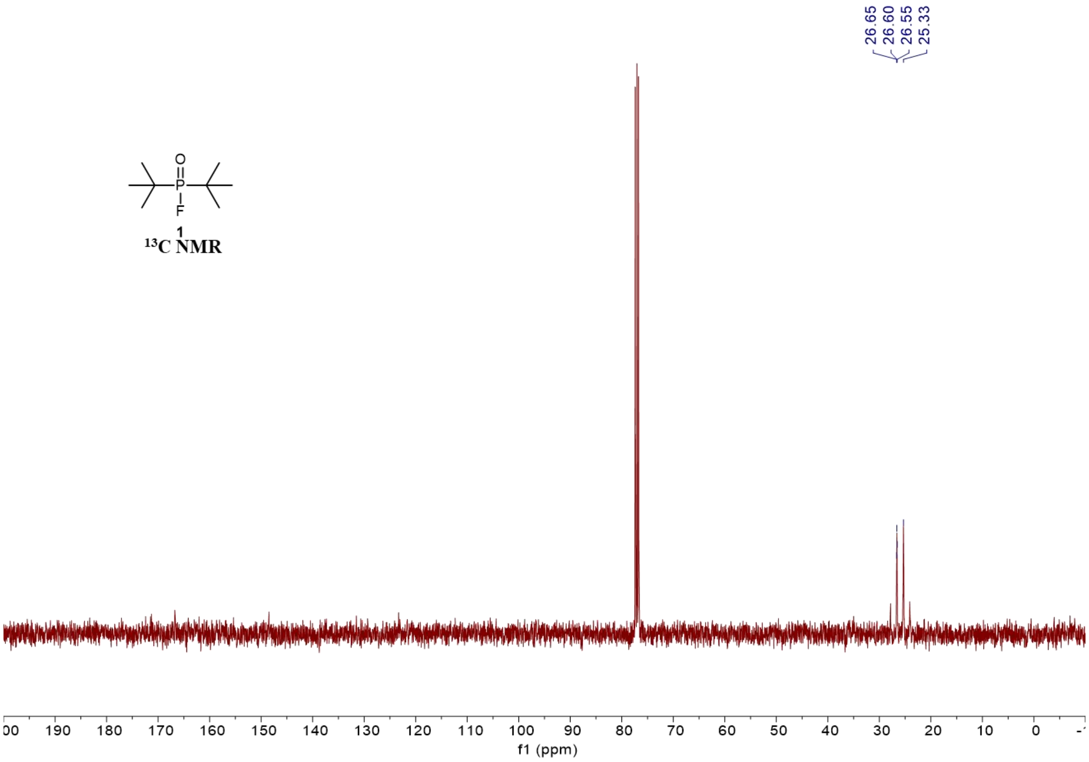

Figure S17. ^{13}C NMR spectrum of compound **1**.

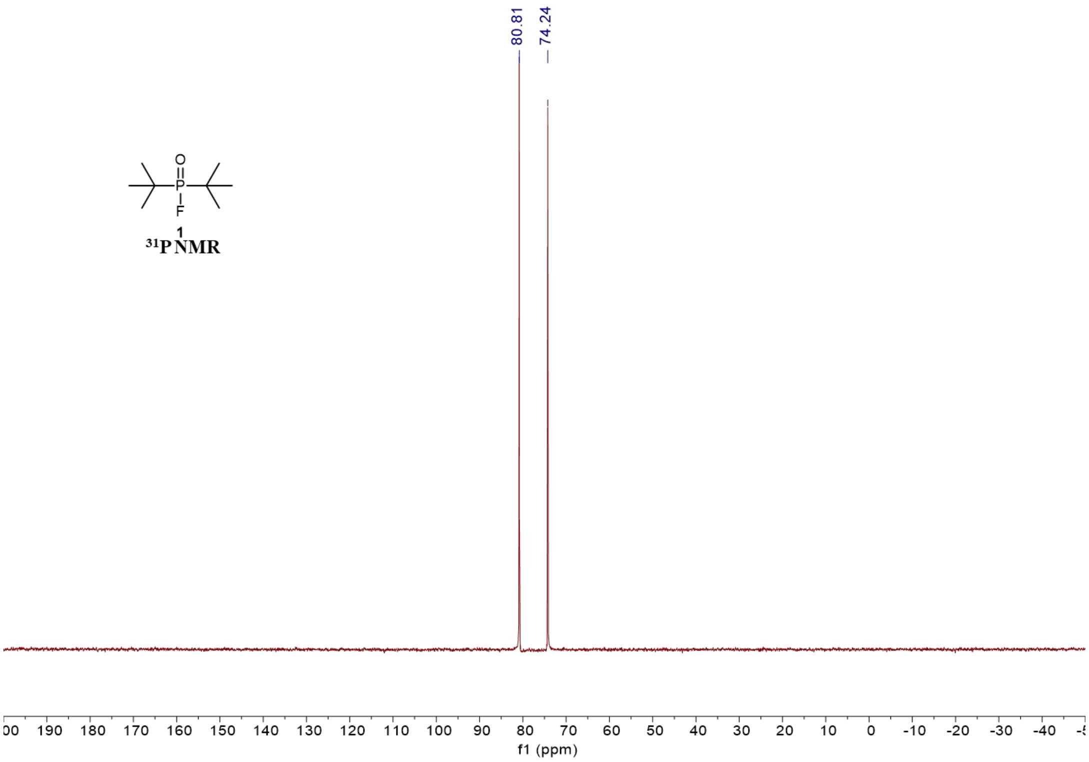

Figure S18. ^{31}P NMR spectrum of compound **1**.

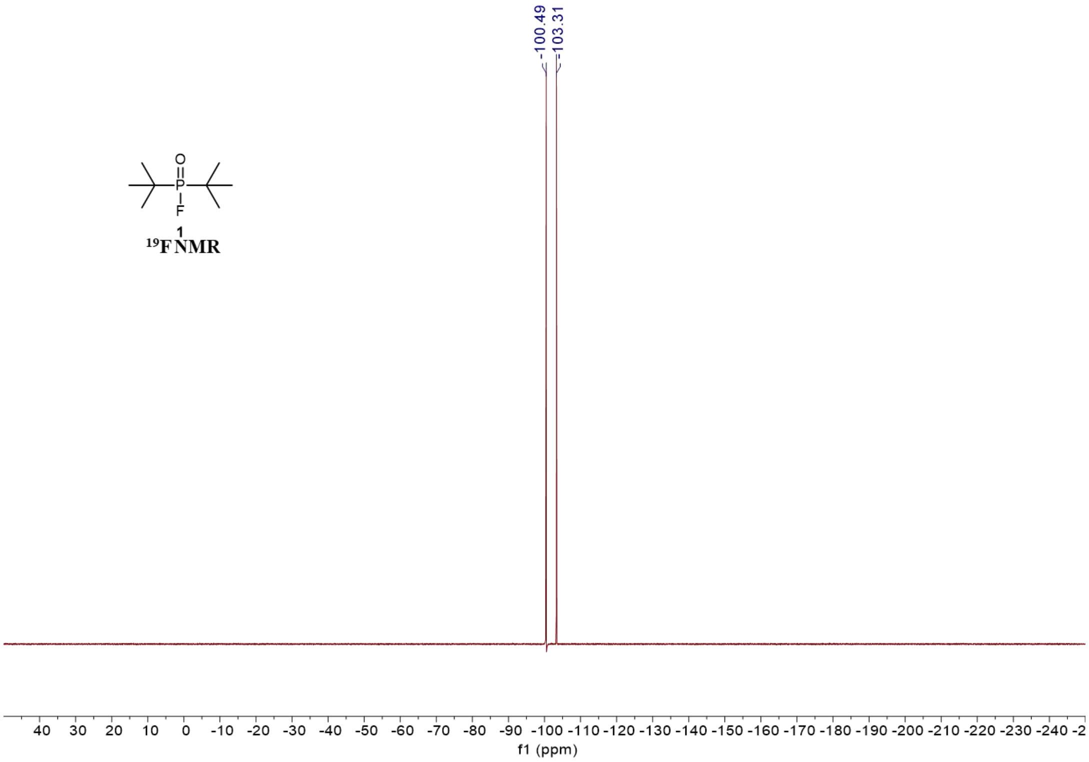

Figure S19. ^{19}F NMR spectrum of compound **1**.

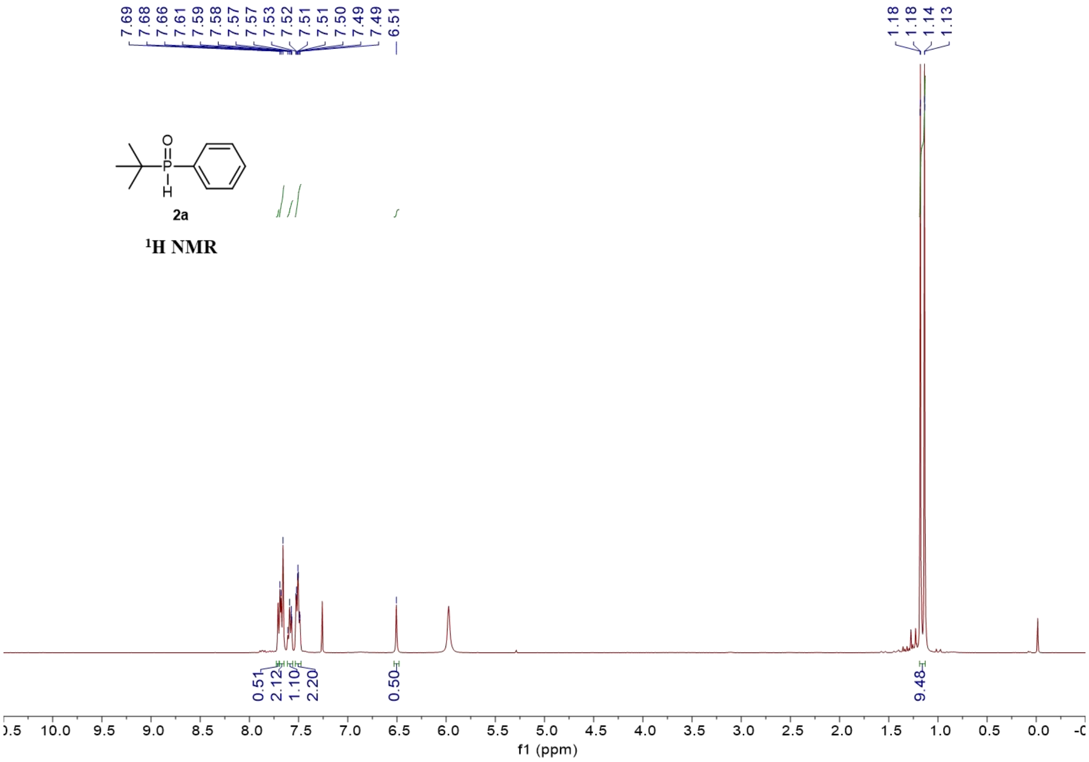

Figure S20. ^{1}H NMR spectrum of compound **2a**.

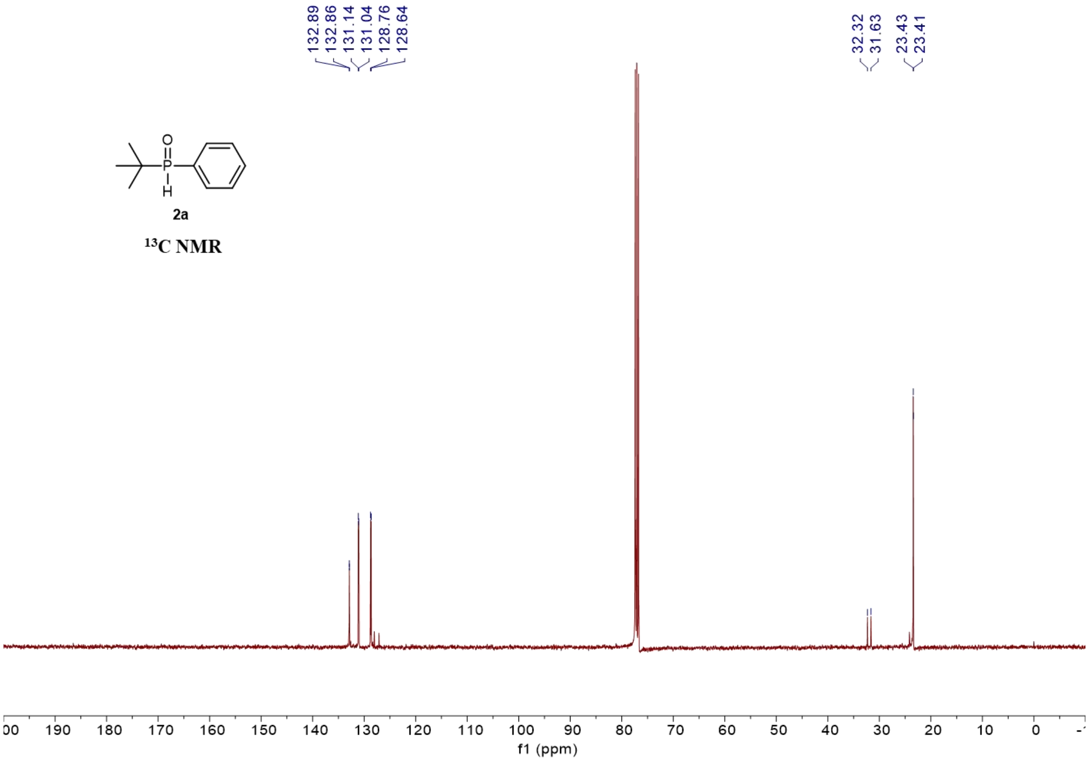

Figure S21. ^{13}C NMR spectrum of compound **2a**.

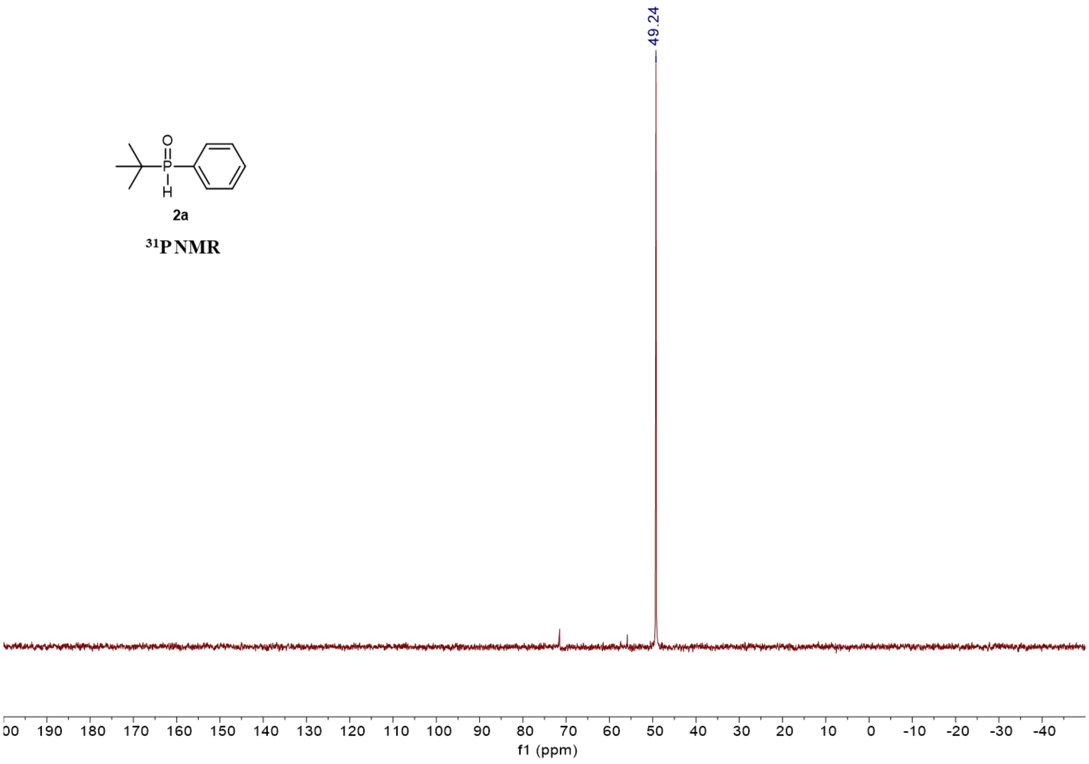

Figure S22. ^{31}P NMR spectrum of compound **2a**.

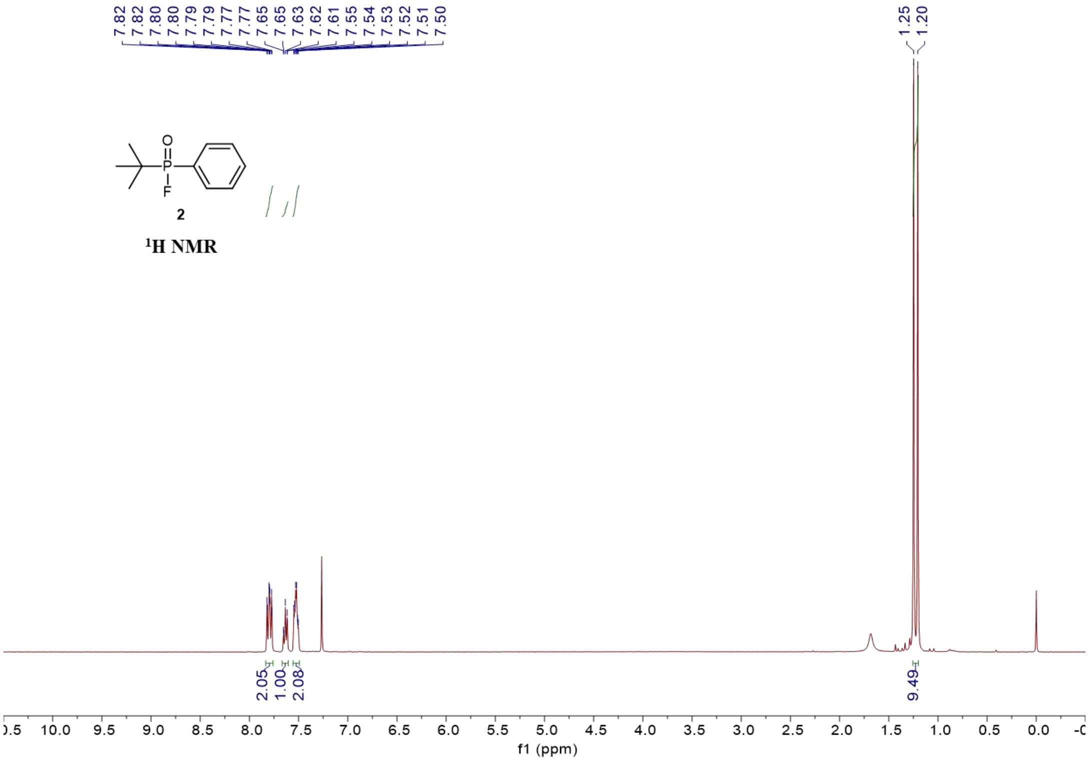

Figure S23. ^{1}H NMR spectrum of compound **2**.

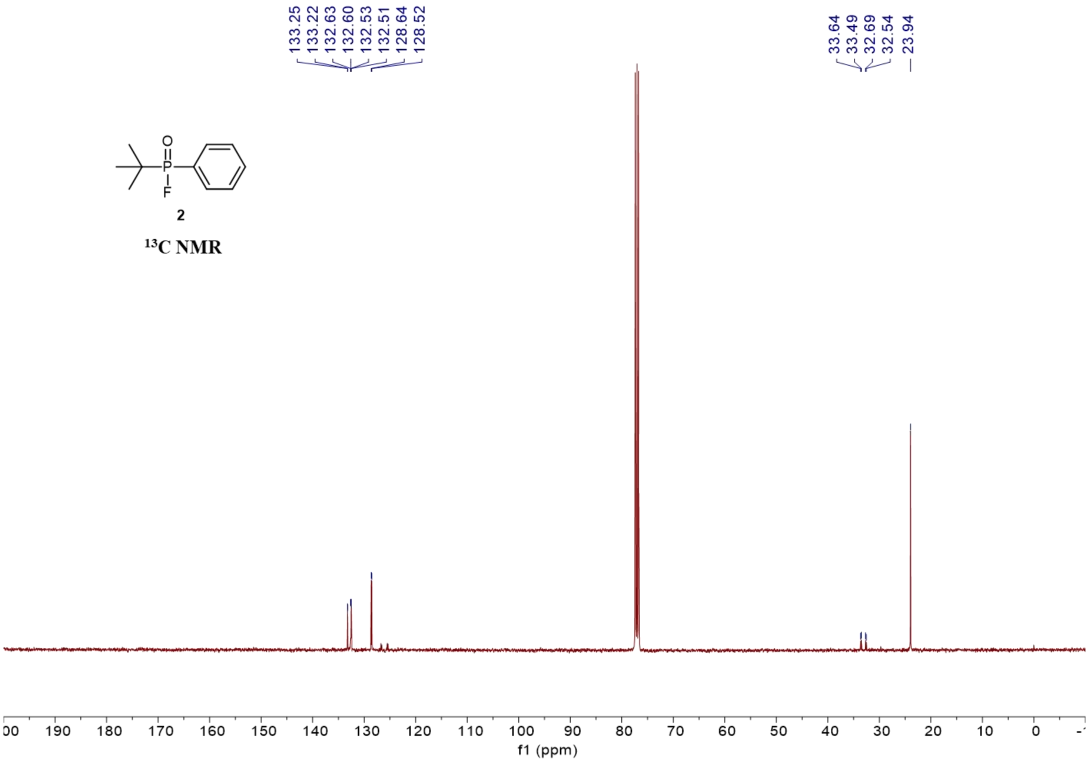

Figure S24. ^{13}C NMR spectrum of compound **2**.

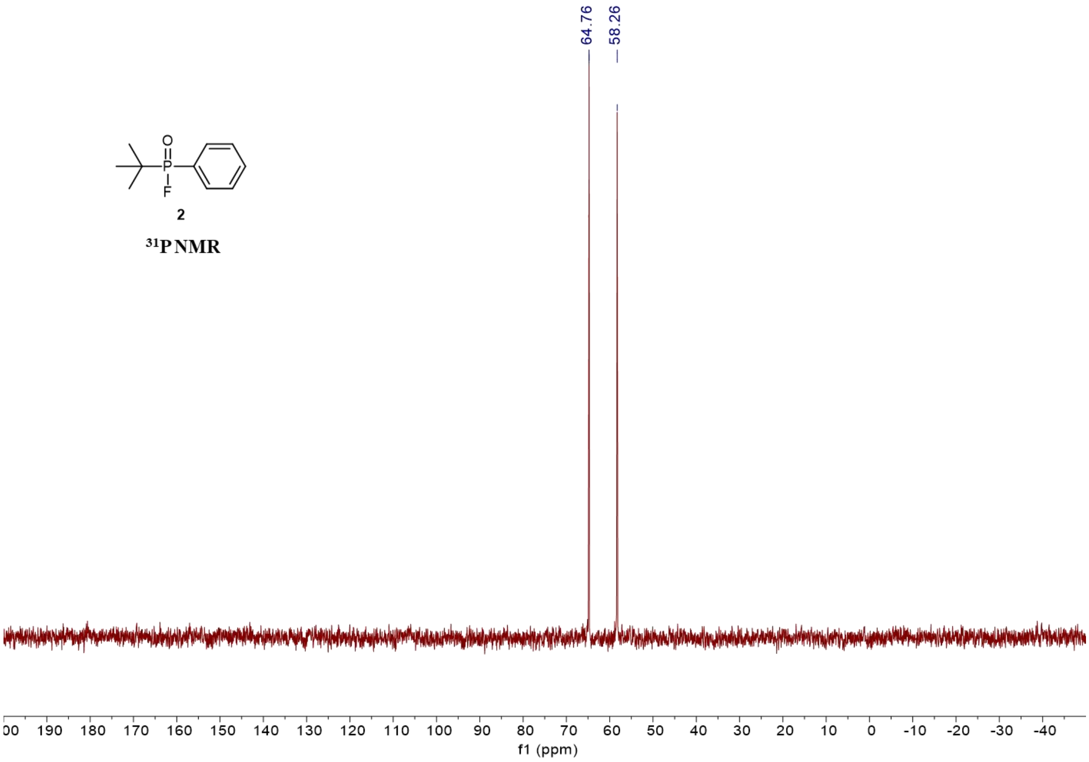

Figure S25. ^{31}P NMR spectrum of compound **2**.

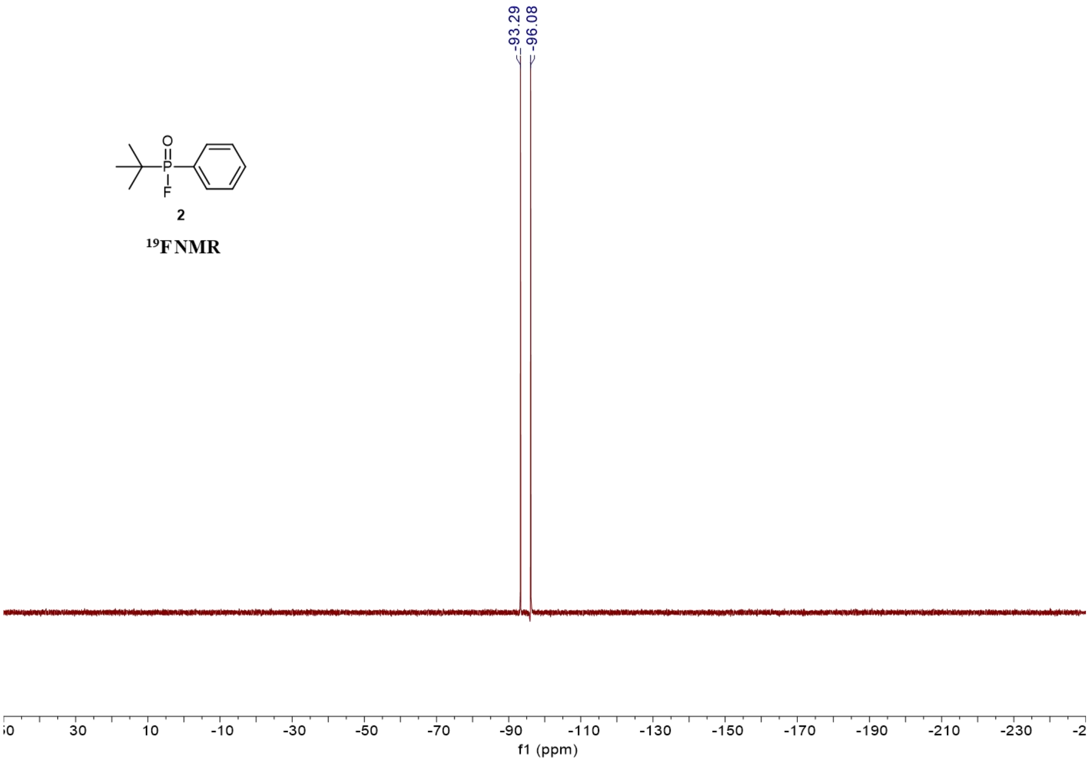

Figure S26. ^{19}F NMR spectrum of compound **2**.

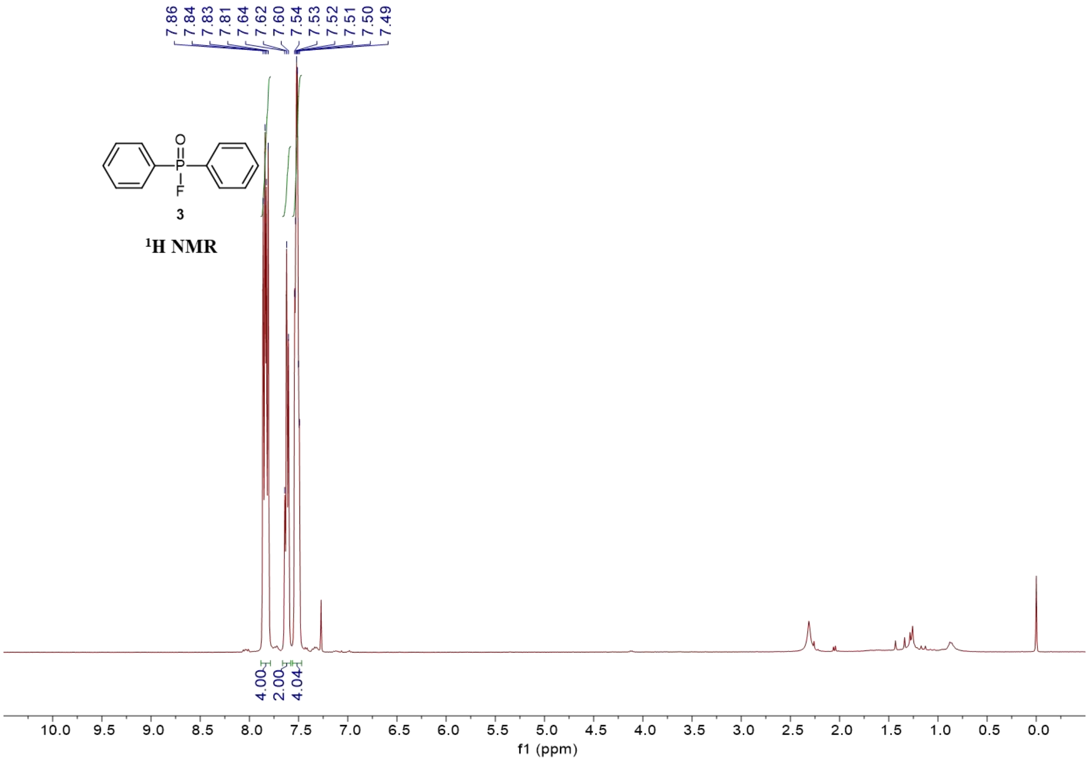

Figure S27. ^{1}H NMR spectrum of compound **3**.

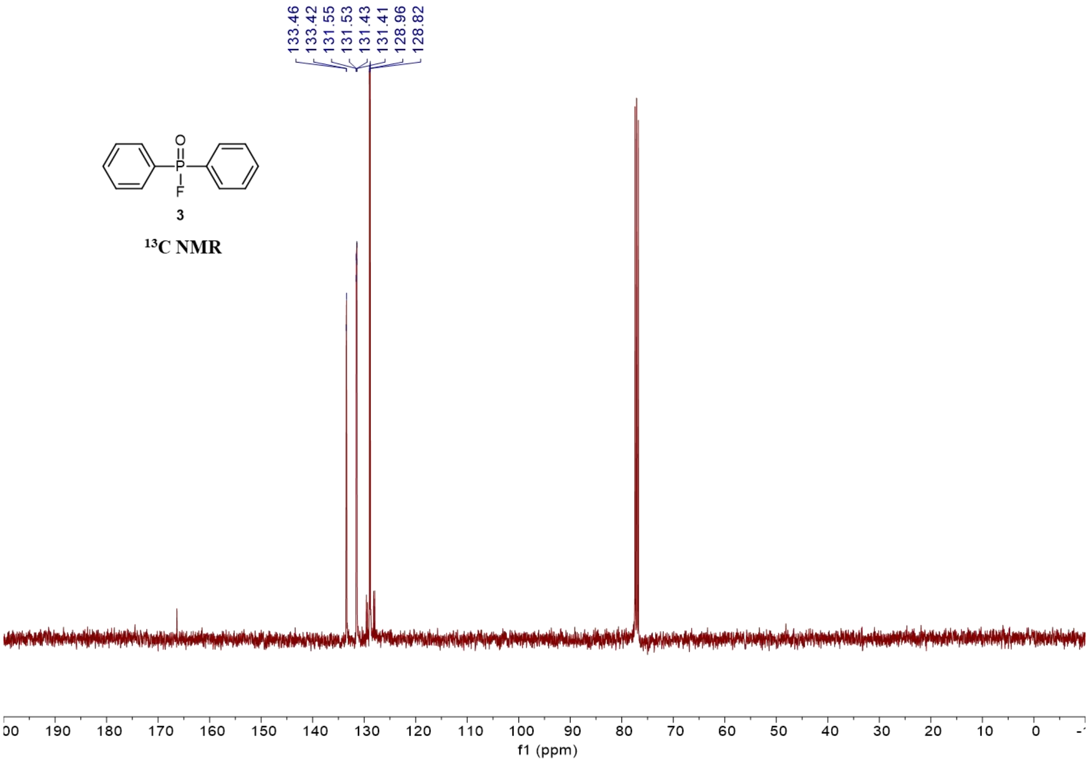

Figure S28. ^{13}C NMR spectrum of compound **3**.

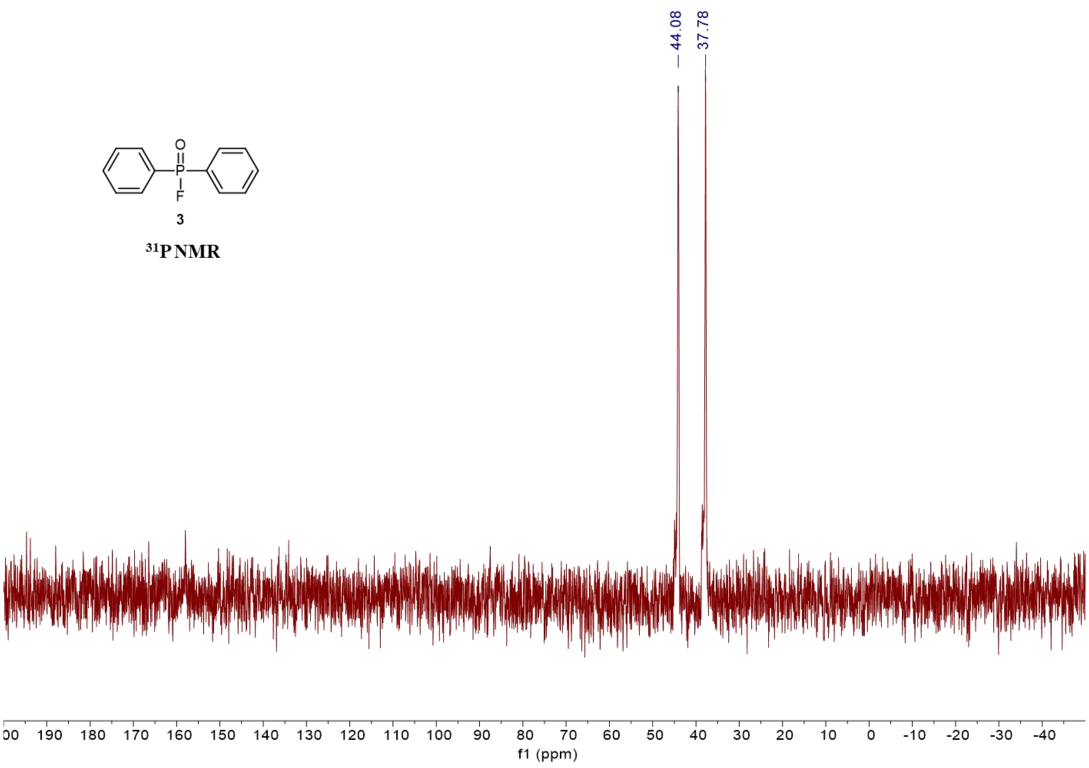

Figure S29. ^{31}P NMR spectrum of compound **3**.

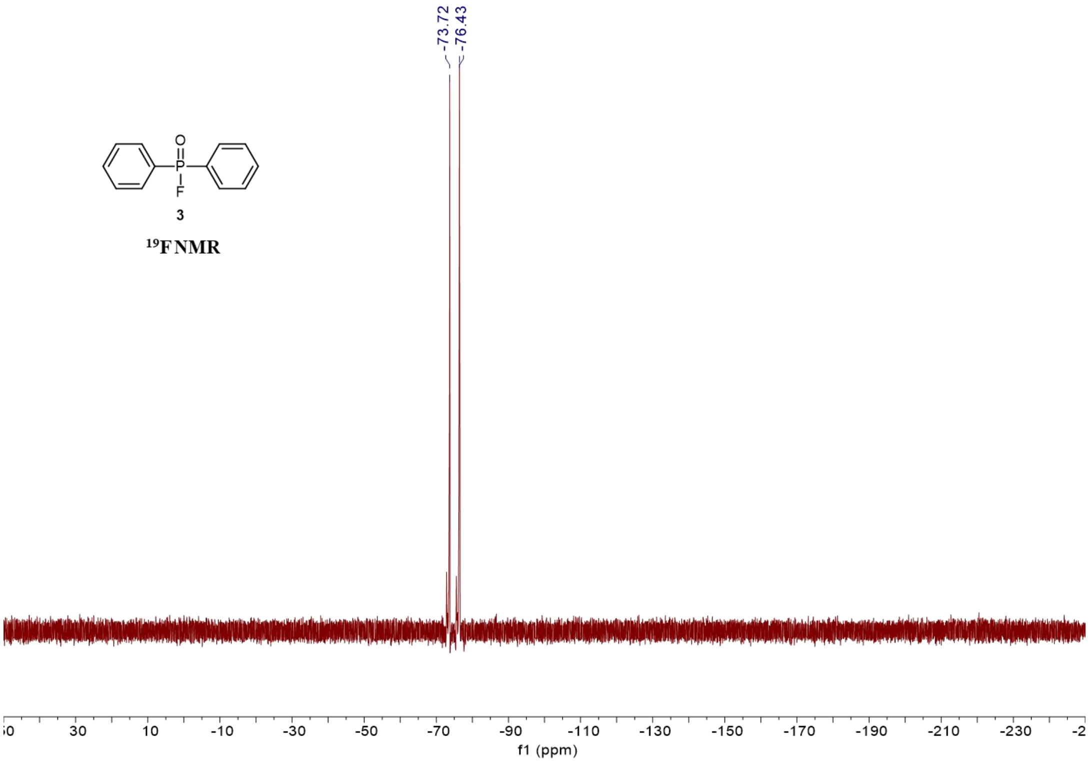

Figure S30. ^{19}F NMR spectrum of compound **3**.

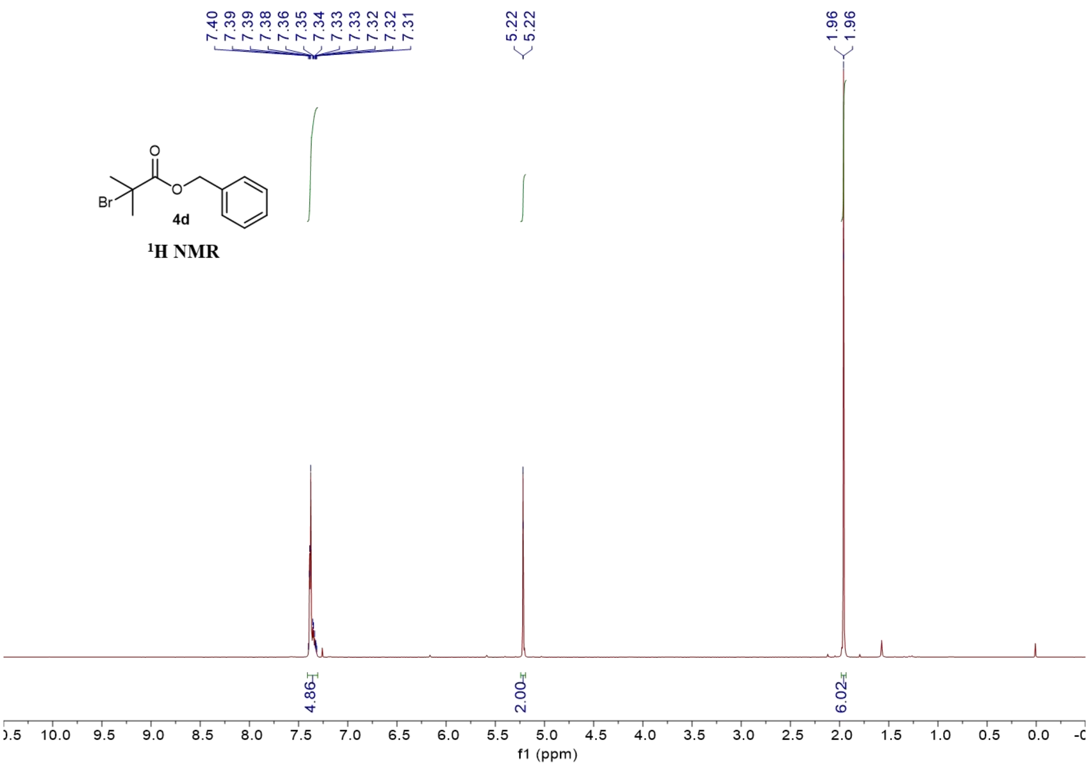

Figure S31. ^{1}H NMR spectrum of compound **4d**.

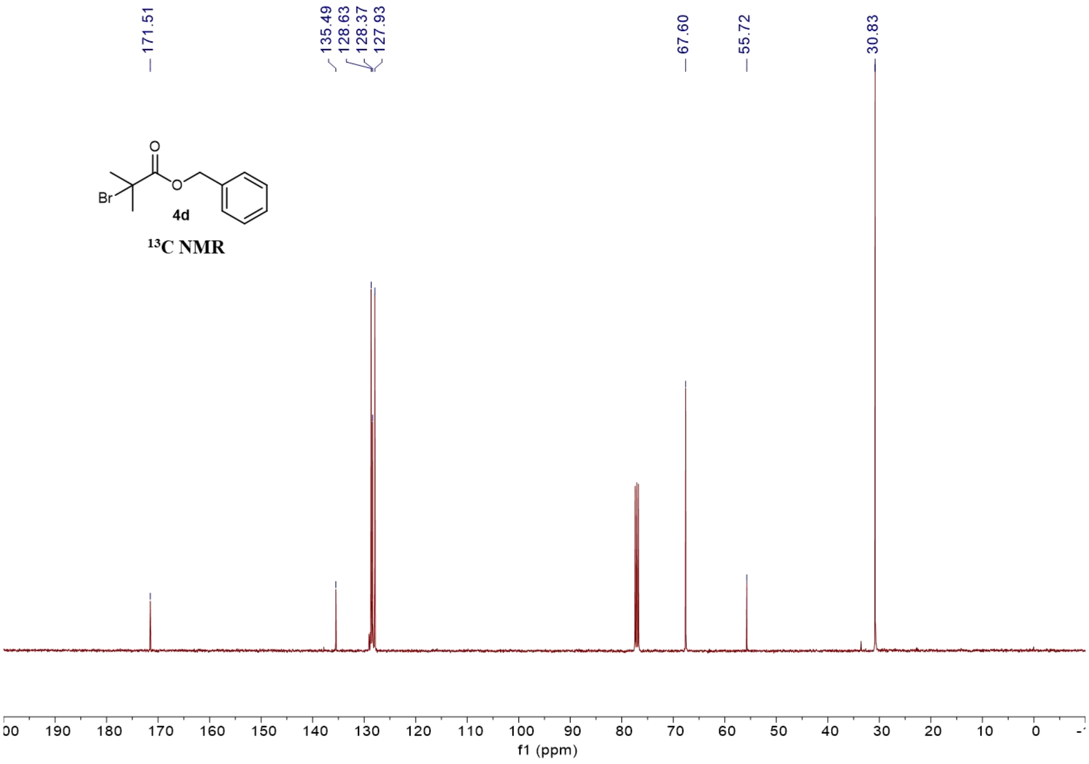

Figure S32. ^{13}C NMR spectrum of compound **4d**.

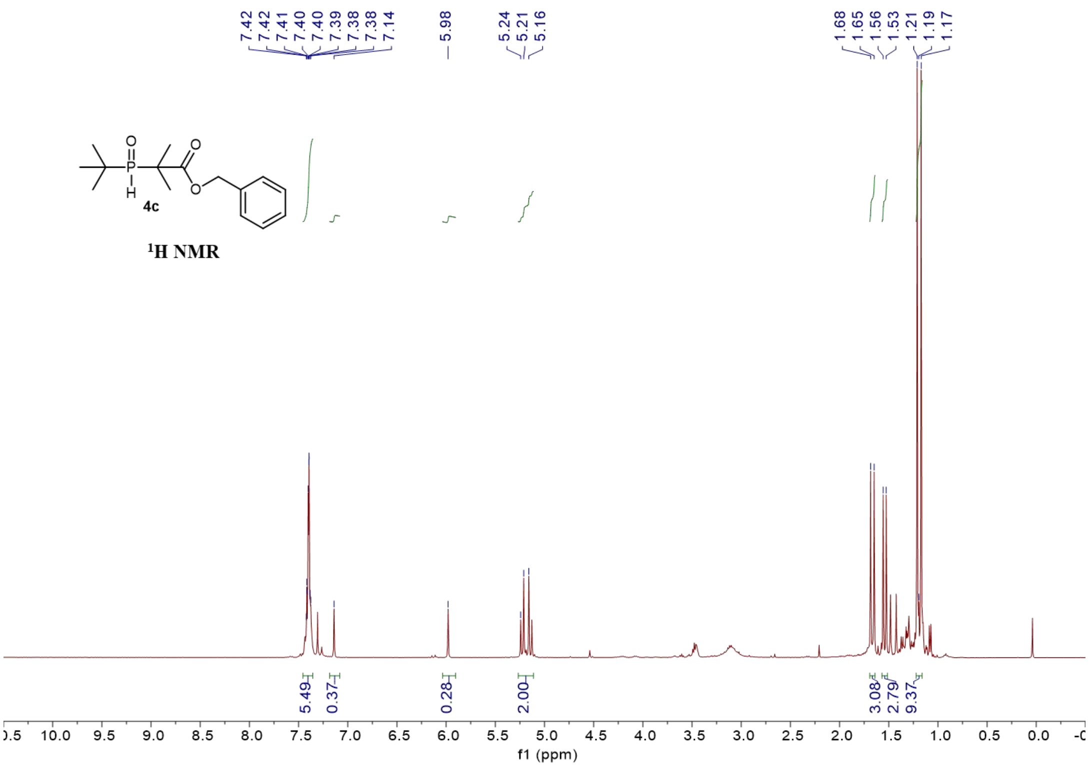

Figure S33. ^{1}H NMR spectrum of compound **4c**.

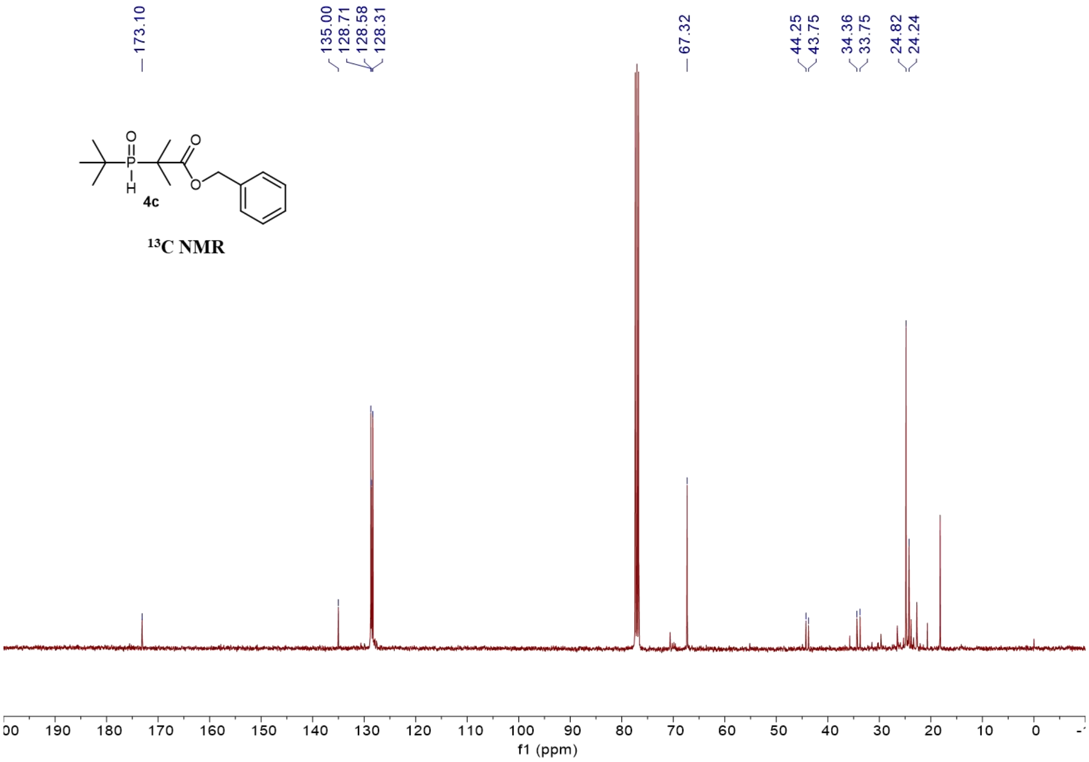

Figure S34. ^{13}C NMR spectrum of compound **4c**.

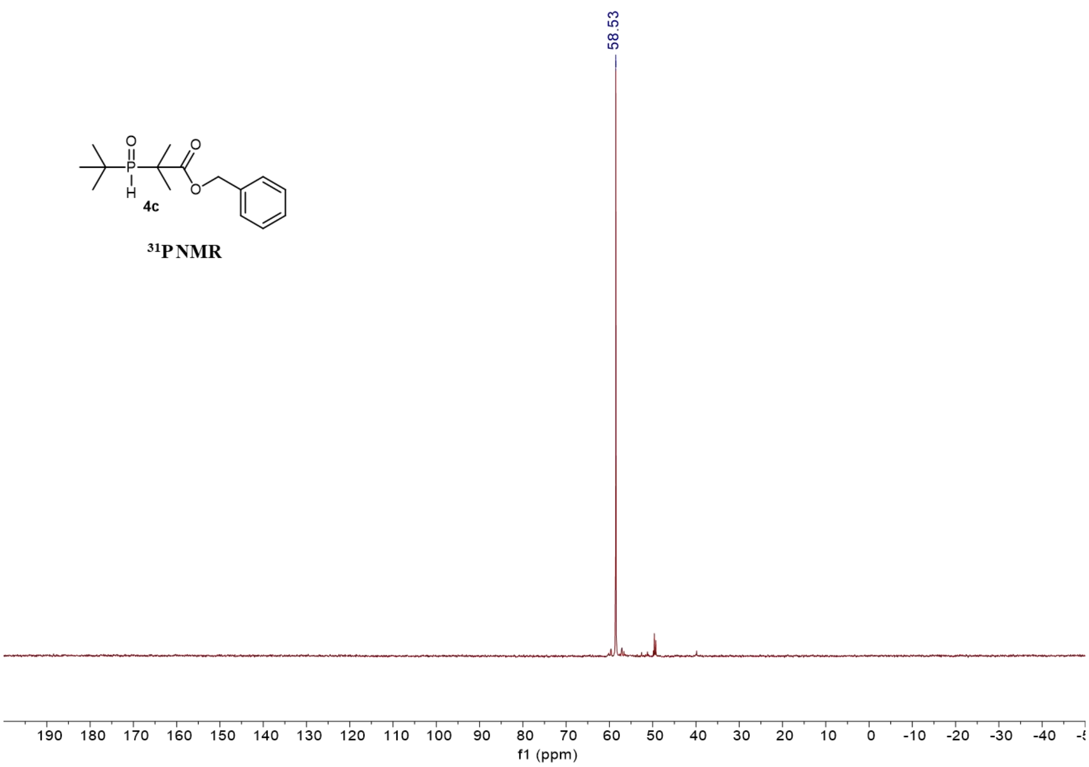

Figure S35. ^{31}P NMR spectrum of compound **4c**.

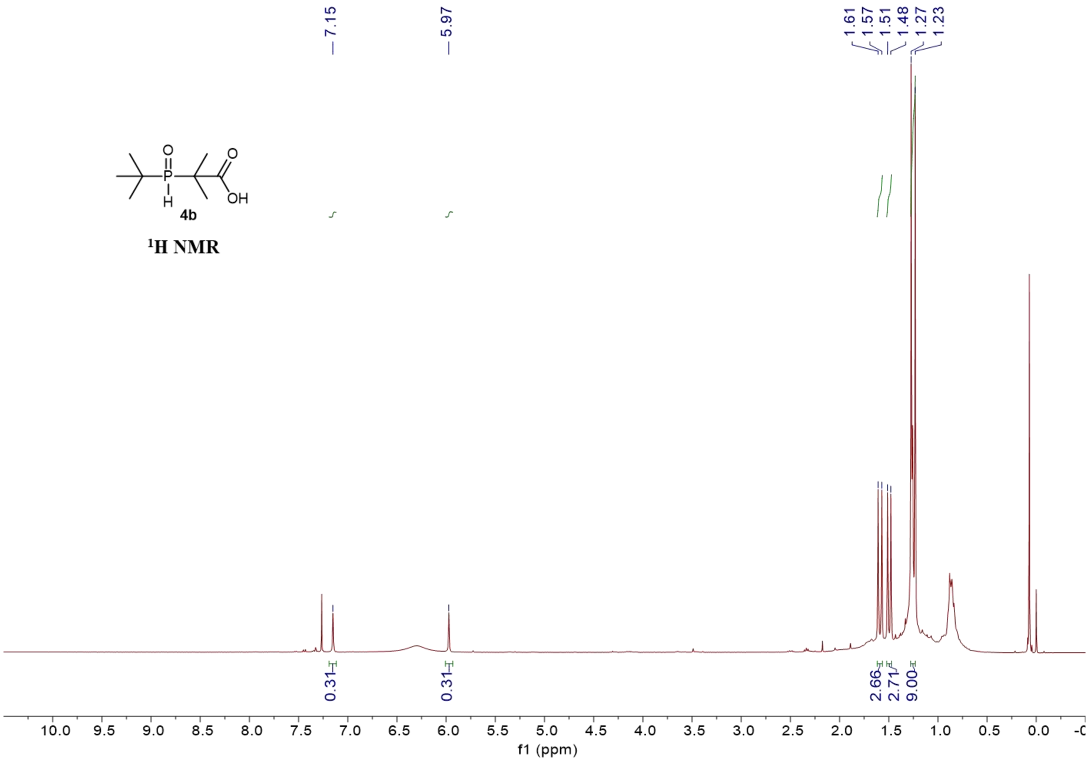

Figure S36. ^{1}H NMR spectrum of compound **4b**.

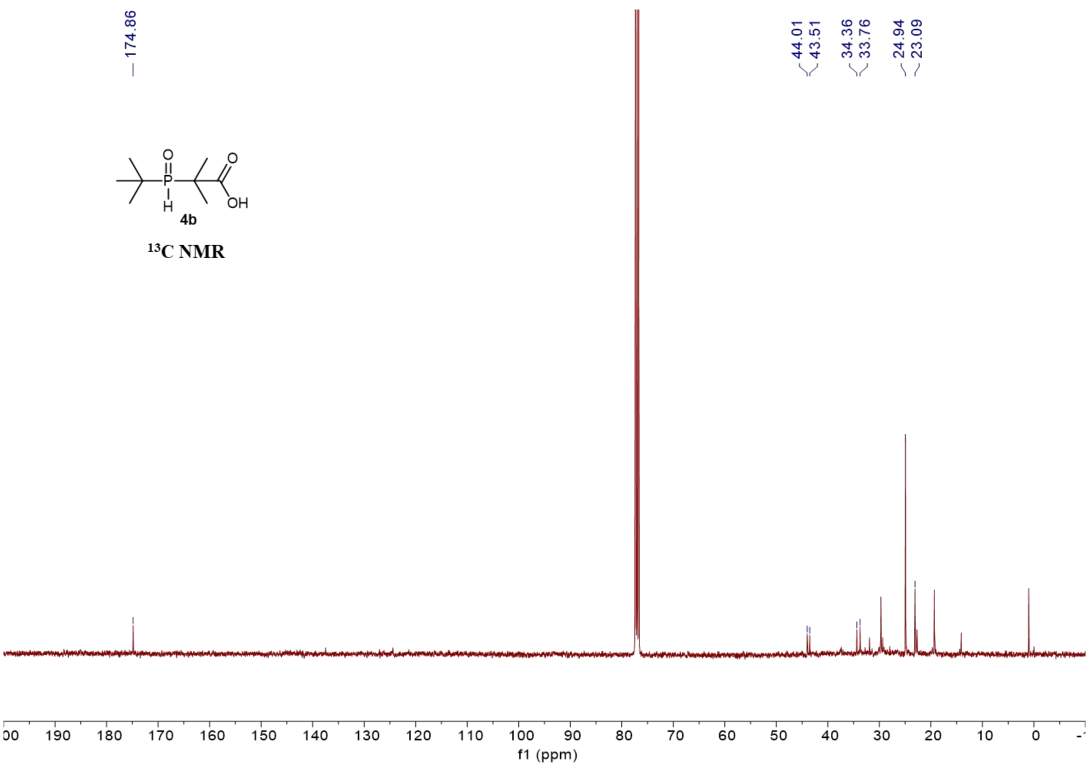

Figure S37. ^{13}C NMR spectrum of compound **4b**.

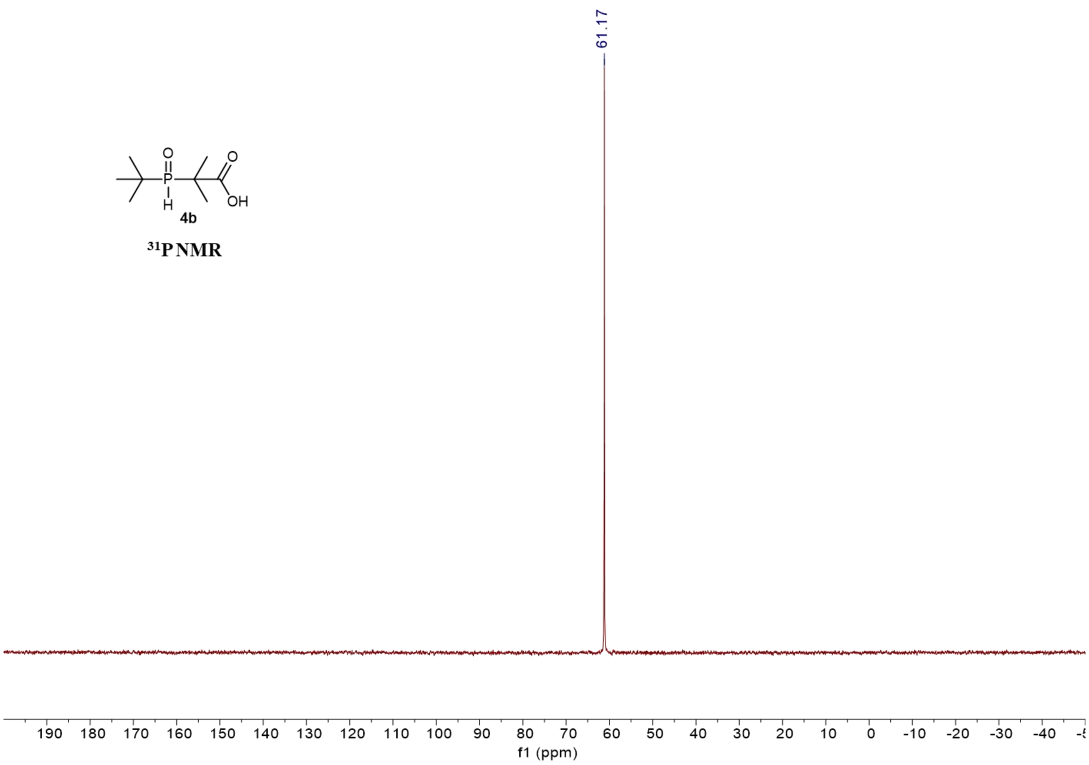

Figure S38. ^{31}P NMR spectrum of compound **4b**.

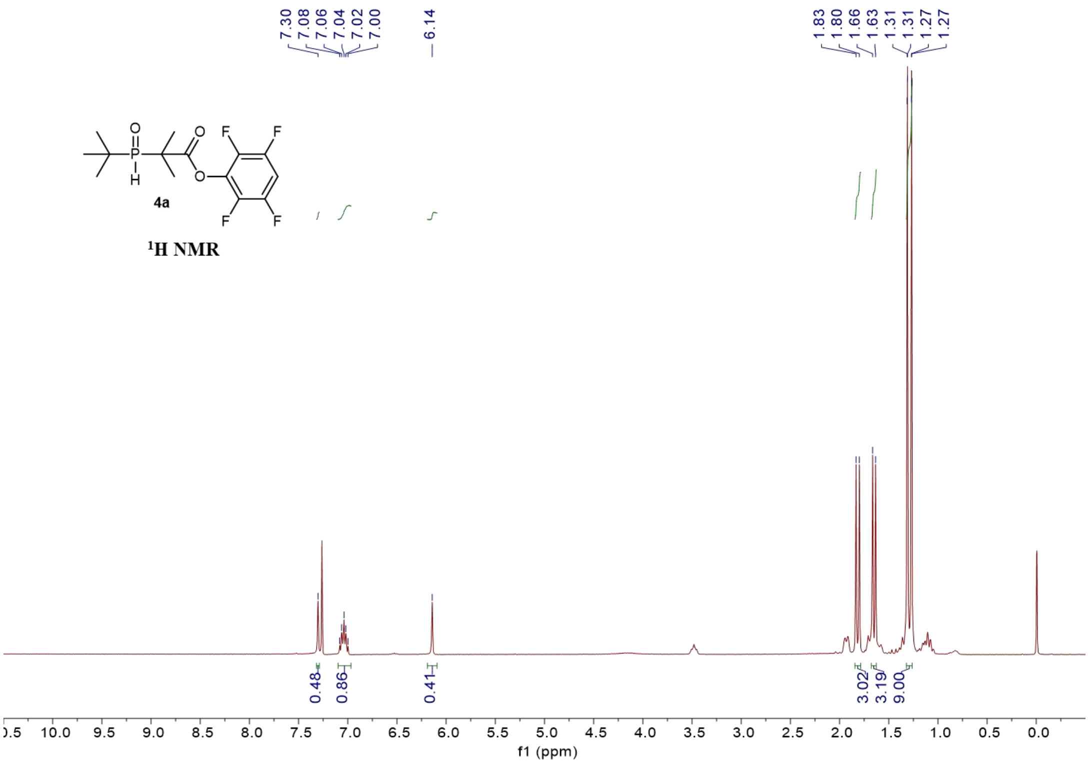

Figure S39. ^{1}H NMR spectrum of compound **4a**.

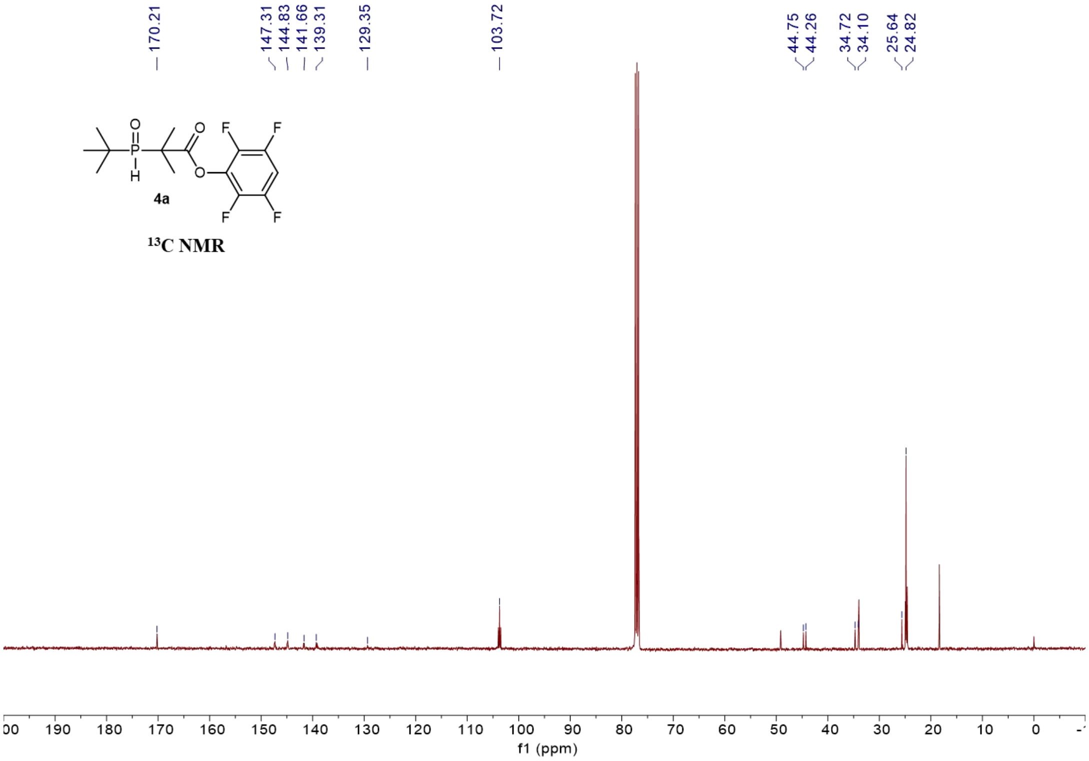

Figure S40. ^{13}C NMR spectrum of compound **4a**.

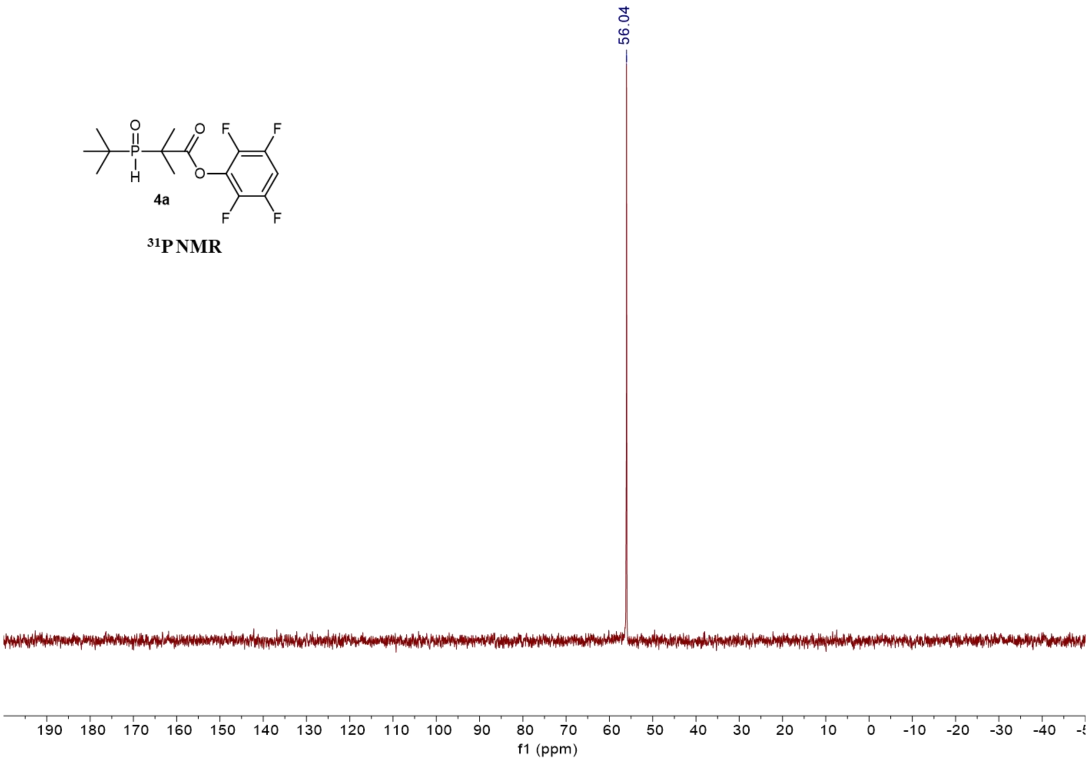

Figure S41. ^{31}P NMR spectrum of compound **4a**.

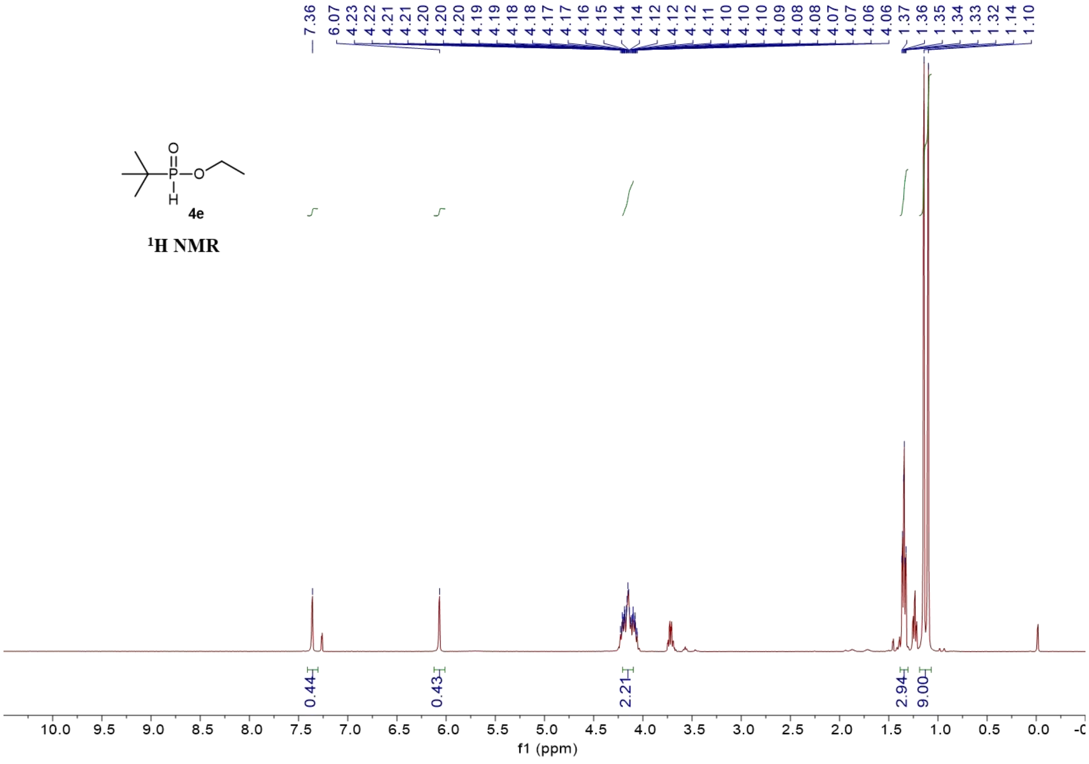

Figure S42. ^{1}H NMR spectrum of compound **4e**.

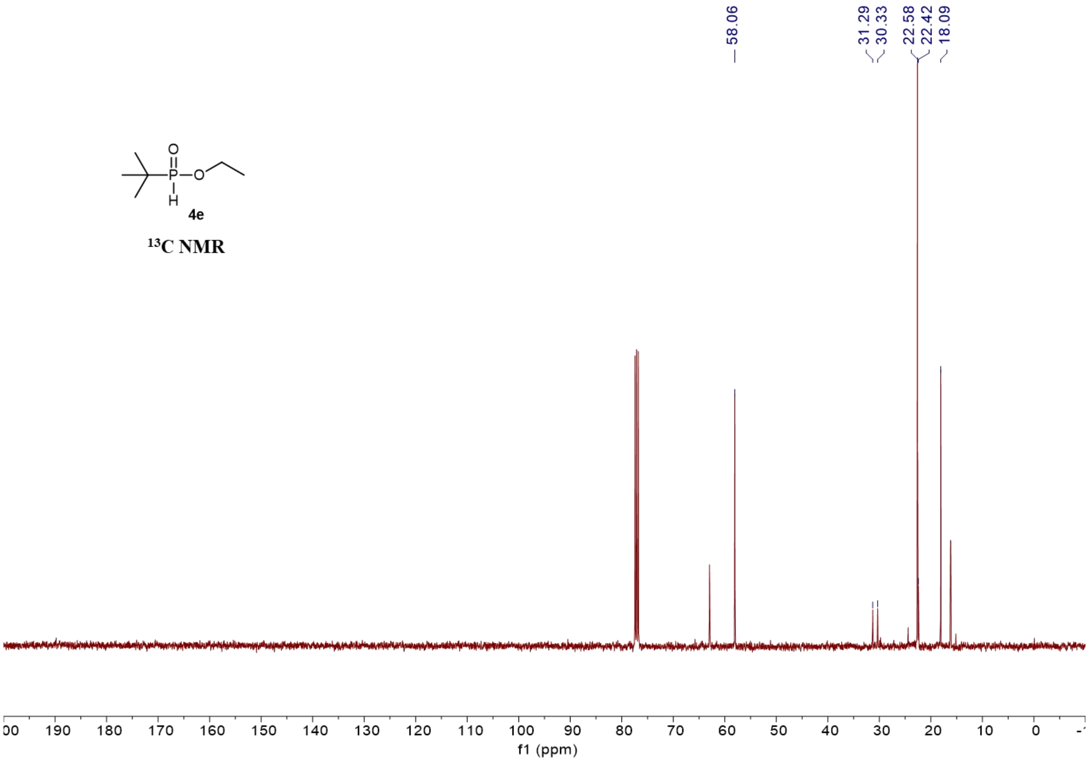

Figure S43. ^{13}C NMR spectrum of compound **4e**.

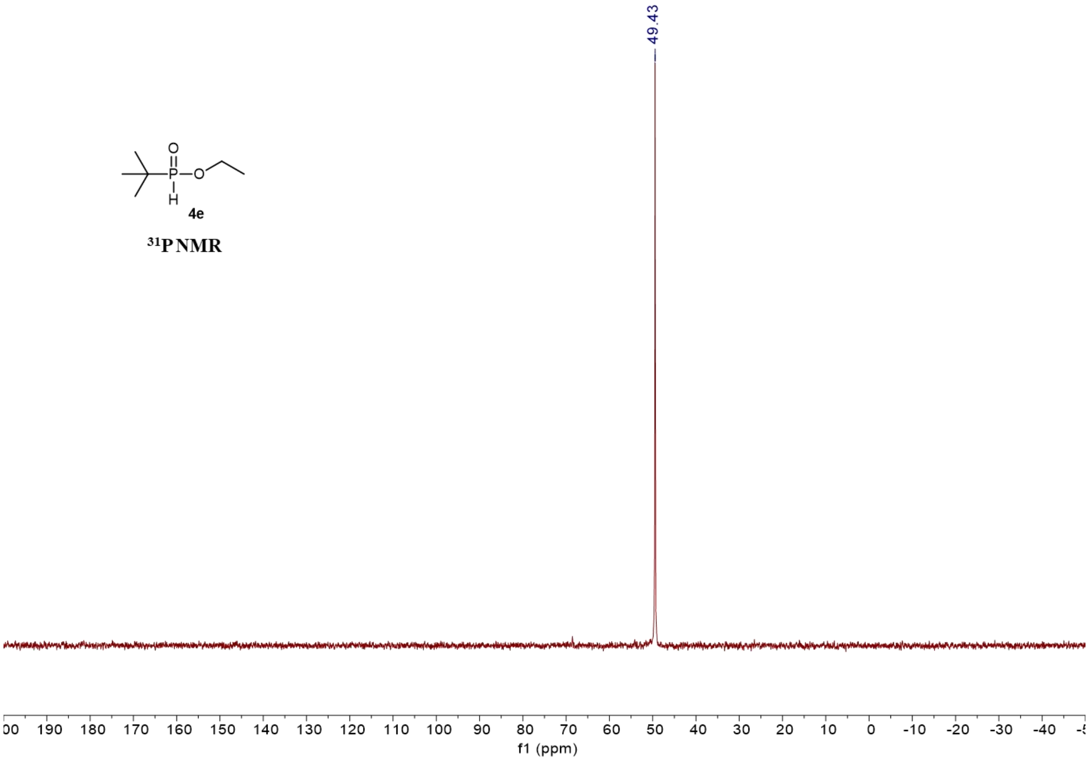

Figure S44. ^{31}P NMR spectrum of compound **4e**.

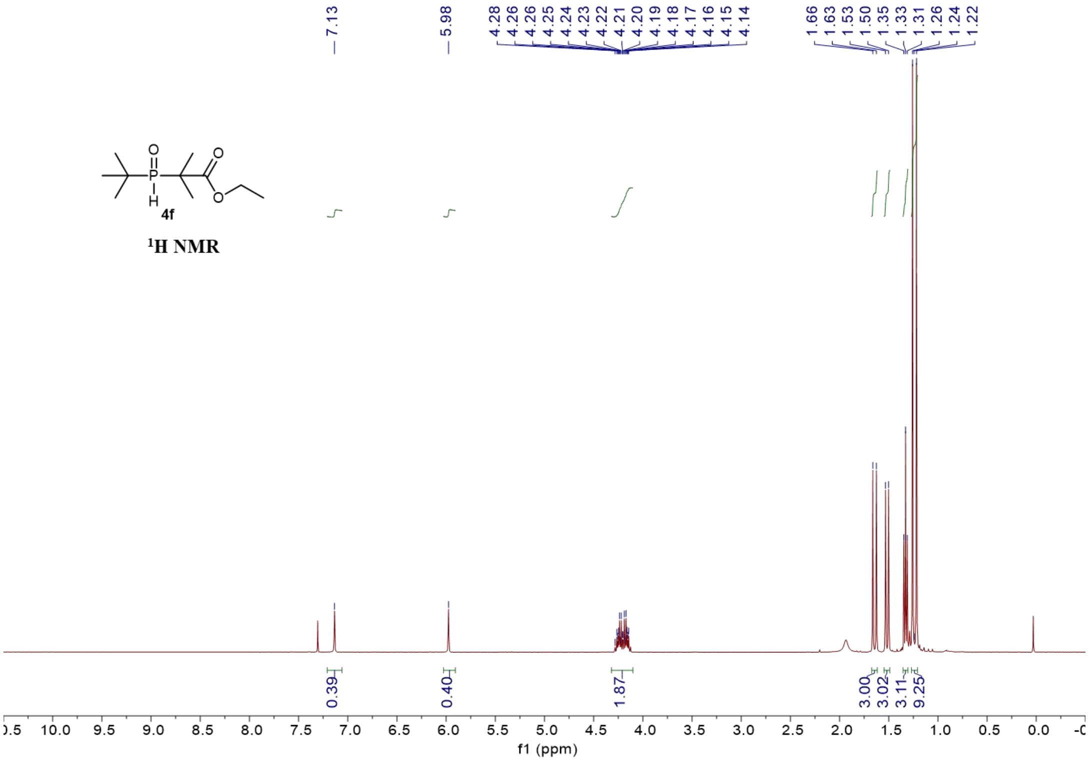

Figure S45. ^{1}H NMR spectrum of compound **4f**.

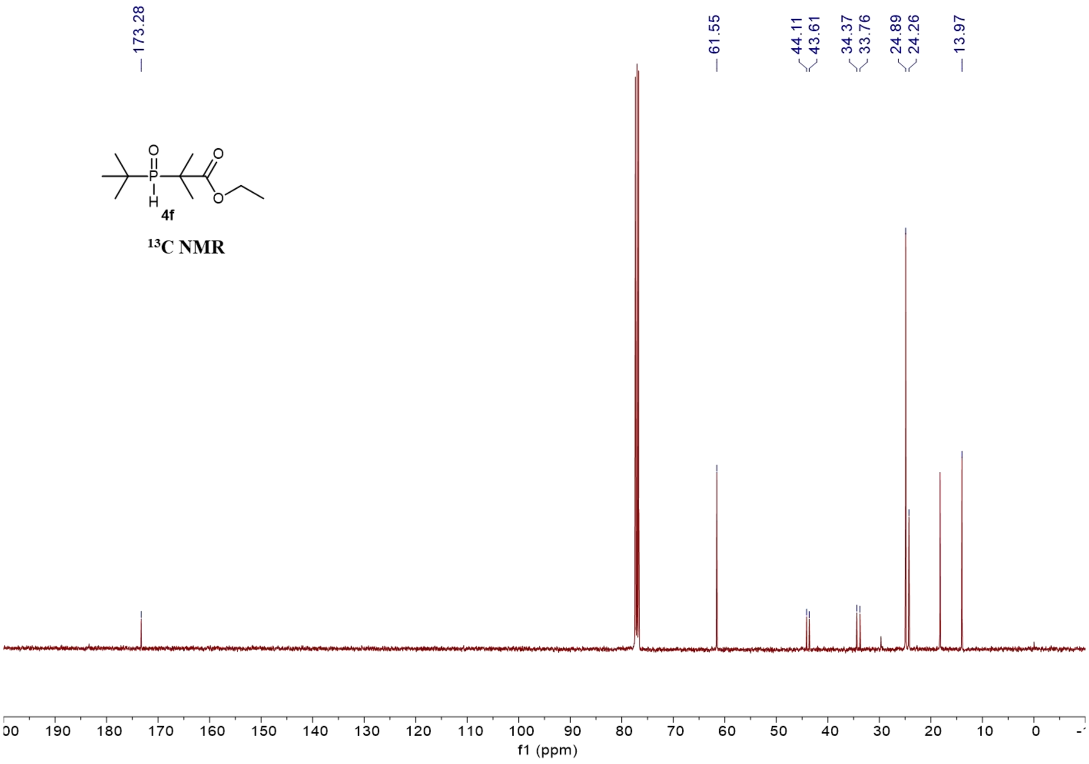

Figure S46. ^{13}C NMR spectrum of compound **4f**.

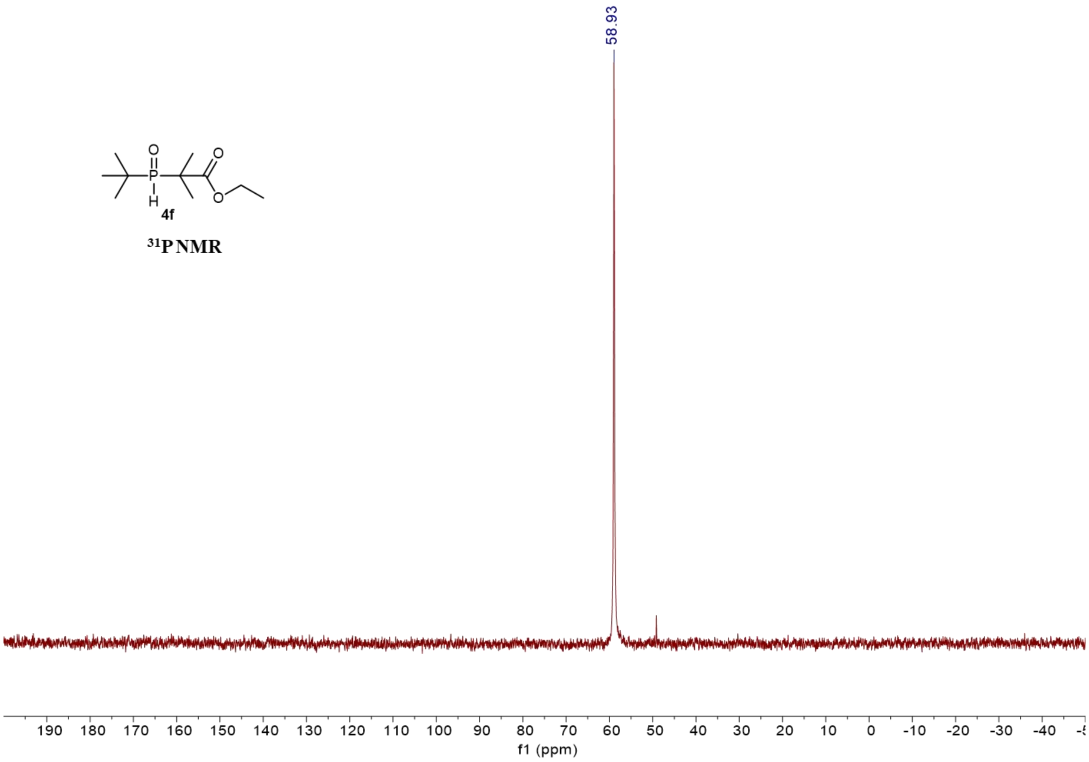

Figure S47. ^{31}P NMR spectrum of compound **4f**.

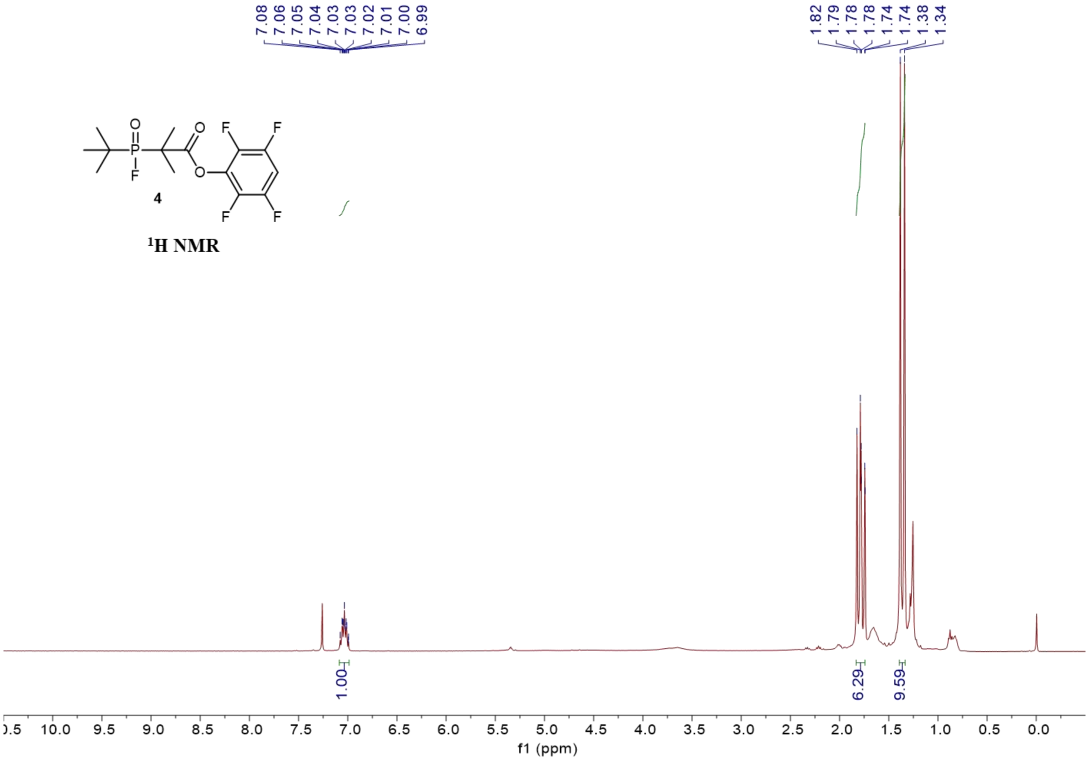

Figure S48. ^{1}H NMR spectrum of compound **4**.

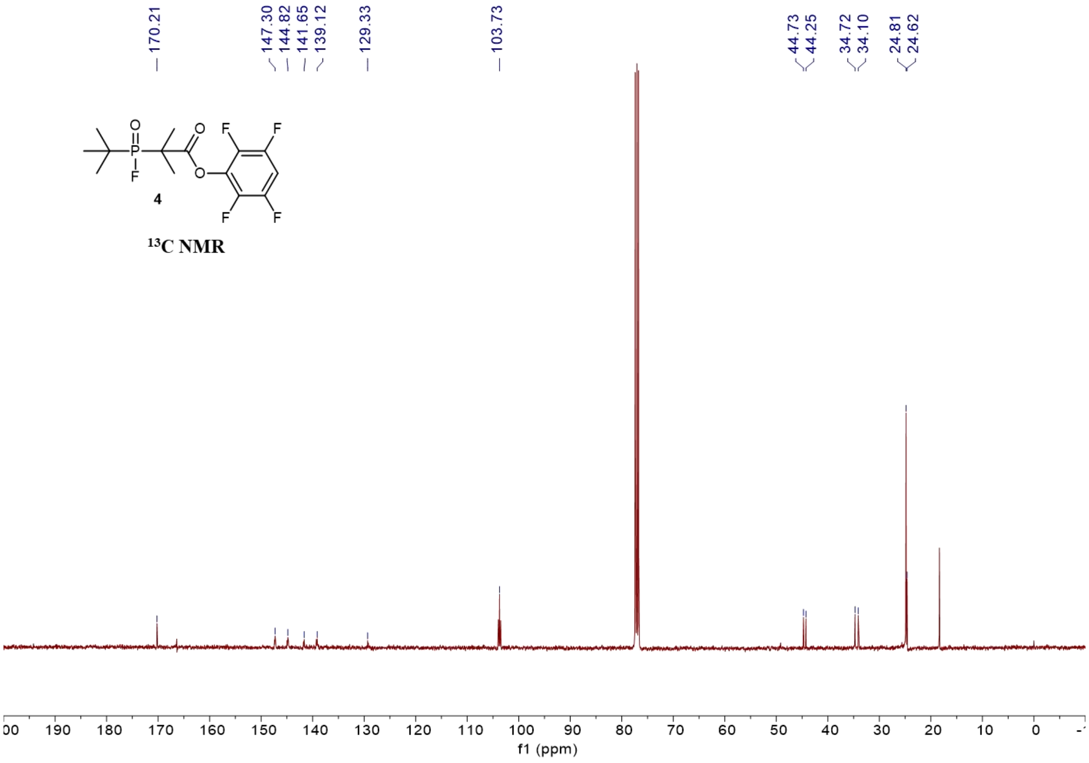

Figure S49. ^{13}C NMR spectrum of compound **4**.

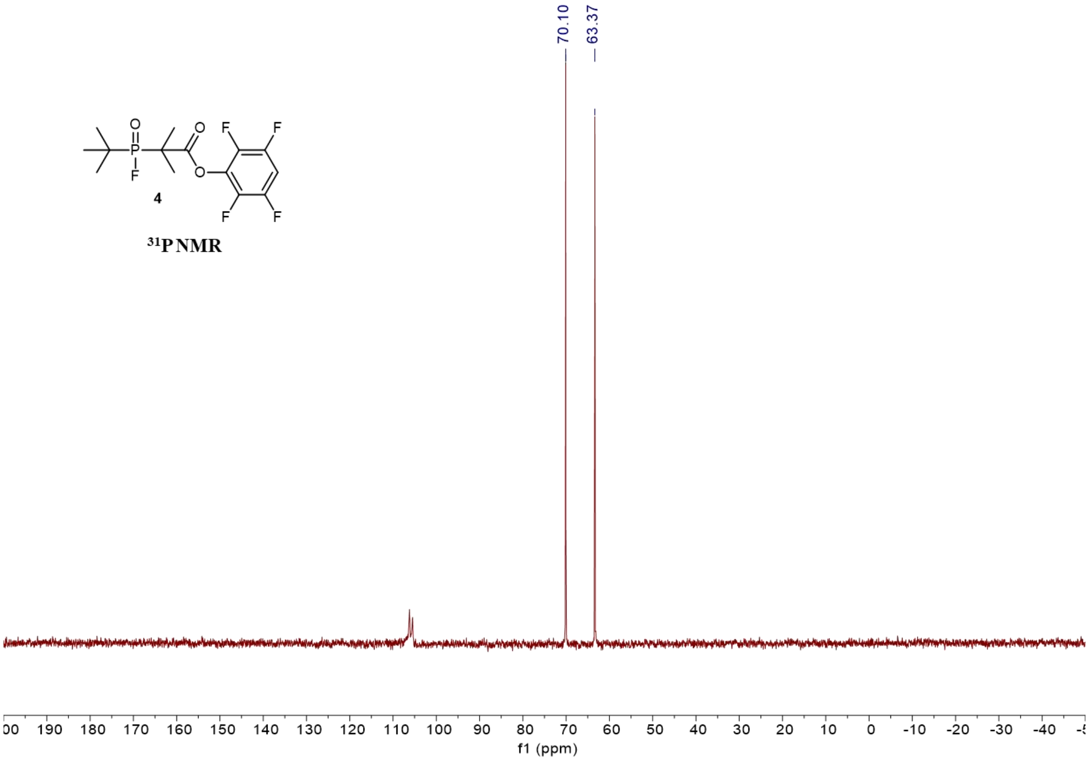

Figure S50. ^{31}P NMR spectrum of compound **4**.

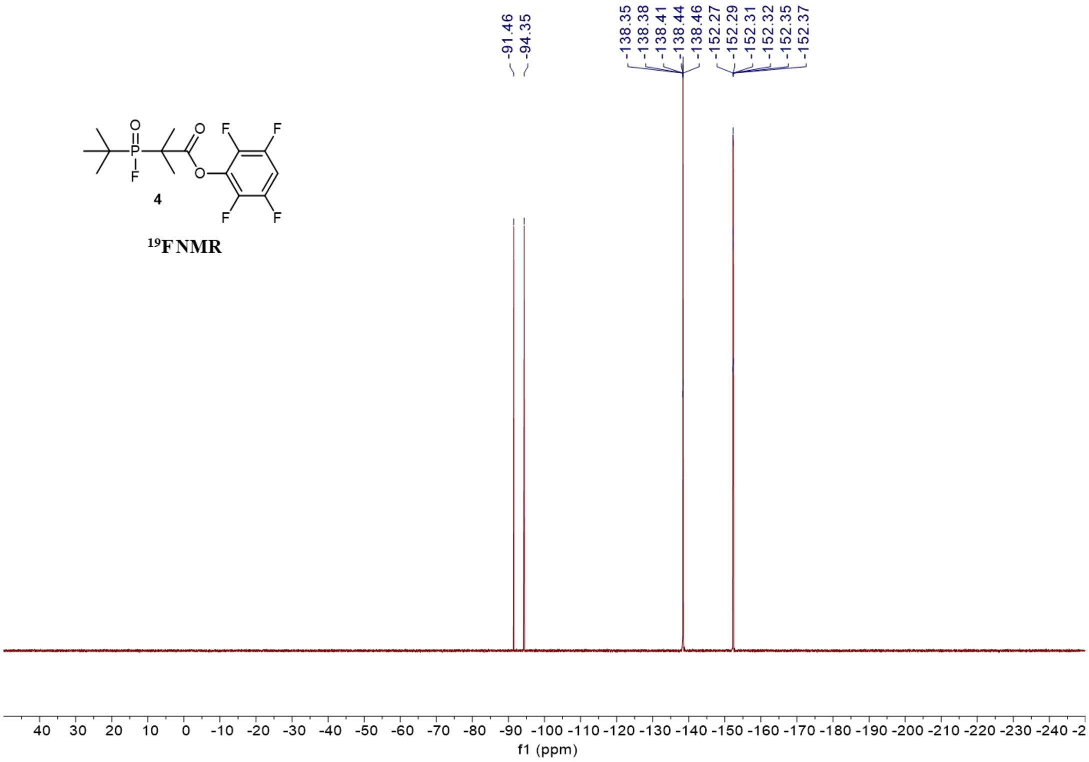

Figure S51. ^{19}F NMR spectrum of compound **4**.

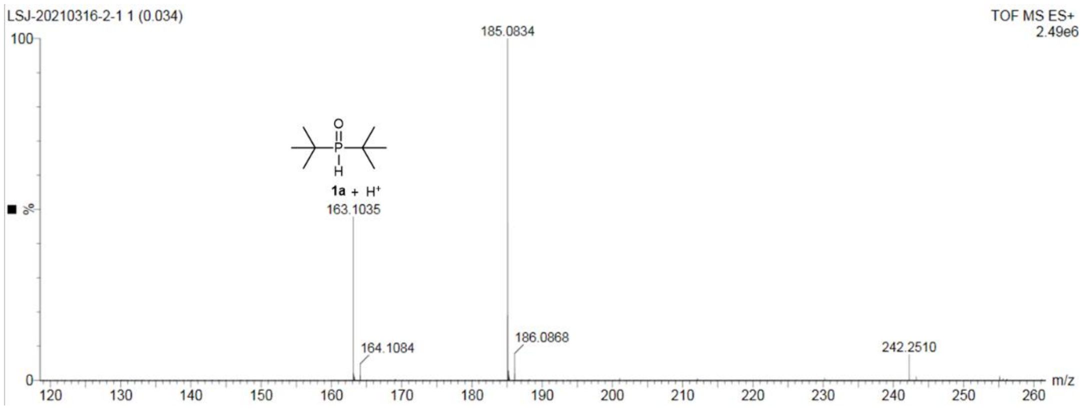

Figure S52. MS spectrum of compound **1a**.

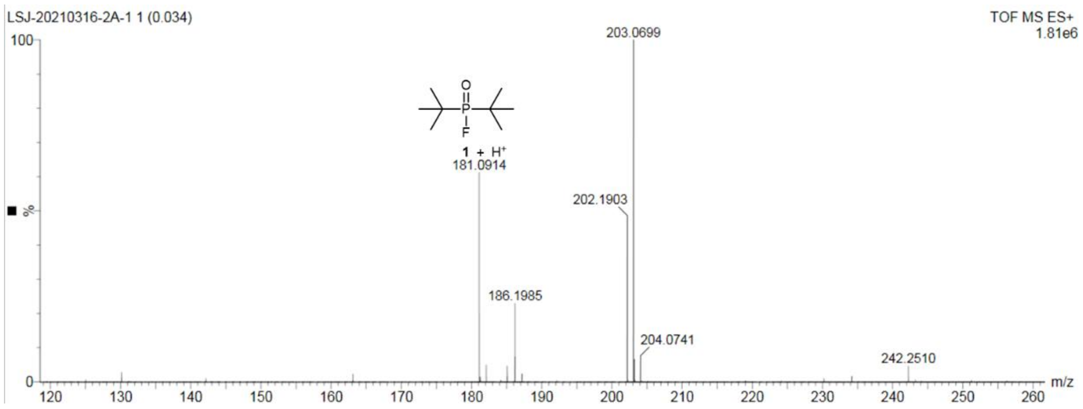

Figure S53. MS spectrum of compound **1**.

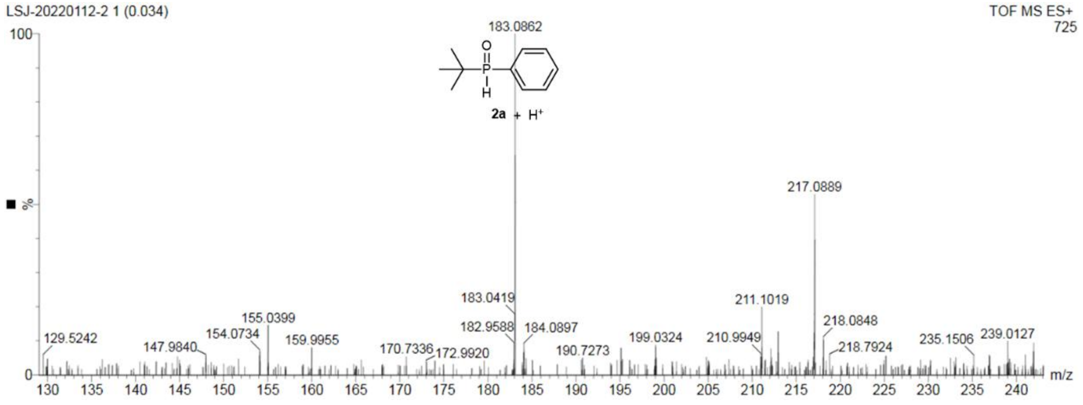

Figure S54. MS spectrum of compound **2a**.

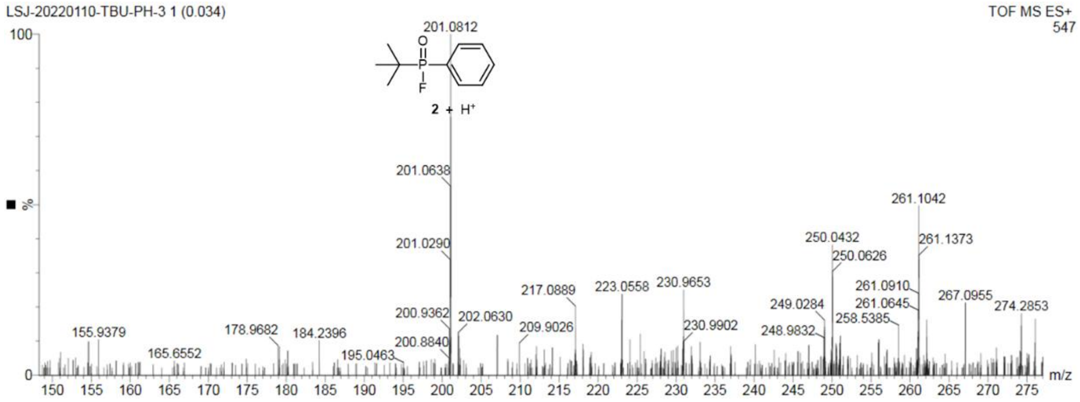

Figure S55. MS spectrum of compound **2**.

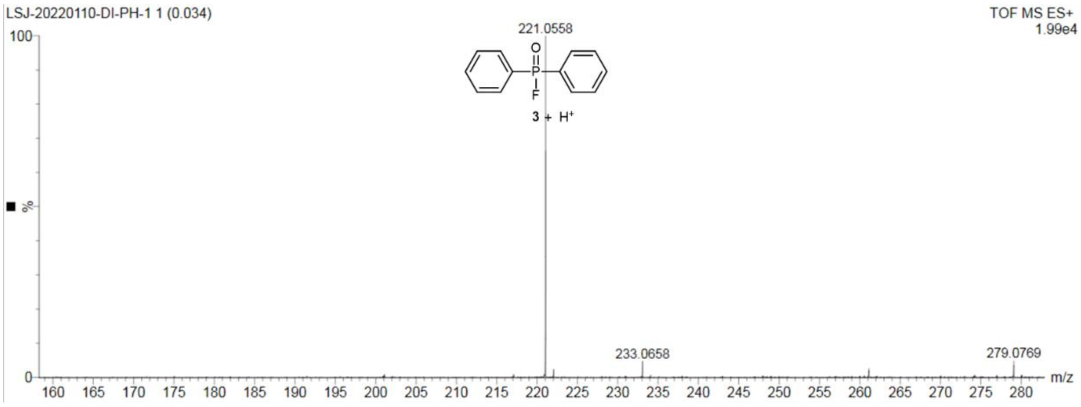

Figure S56. MS spectrum of compound **3**.

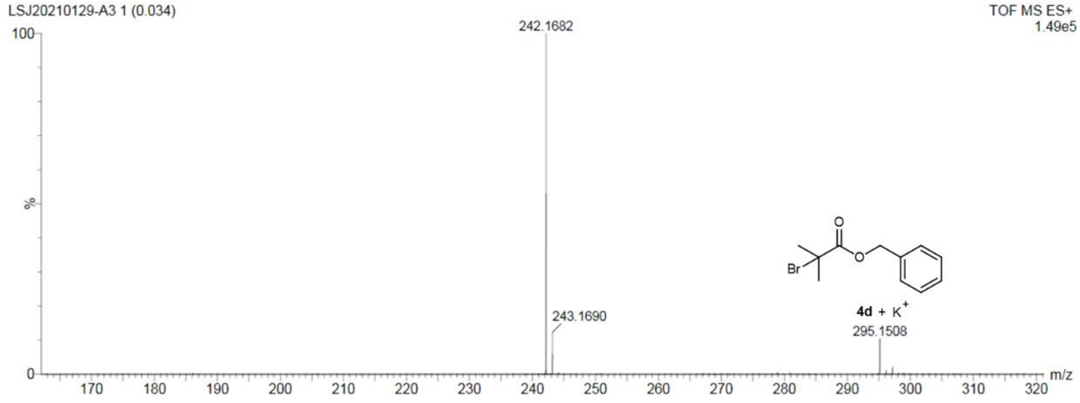

Figure S57. MS spectrum of compound **4d**.

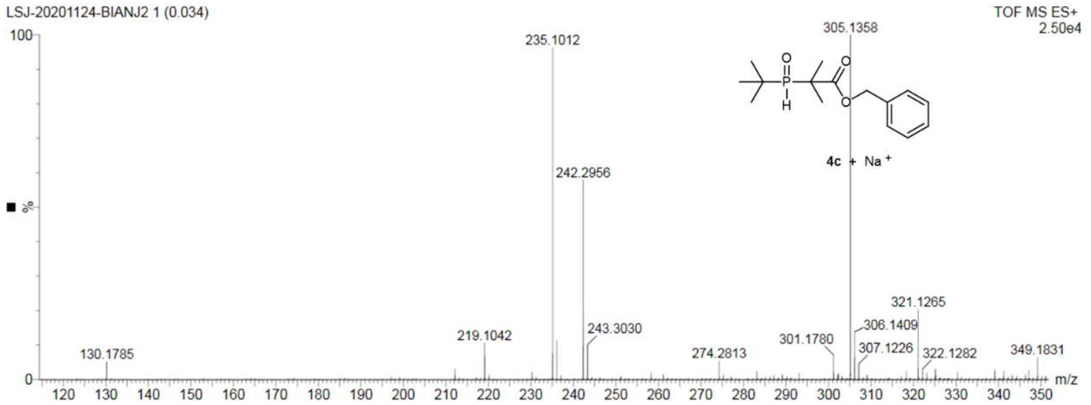

Figure S58. MS spectrum of compound **4c**.

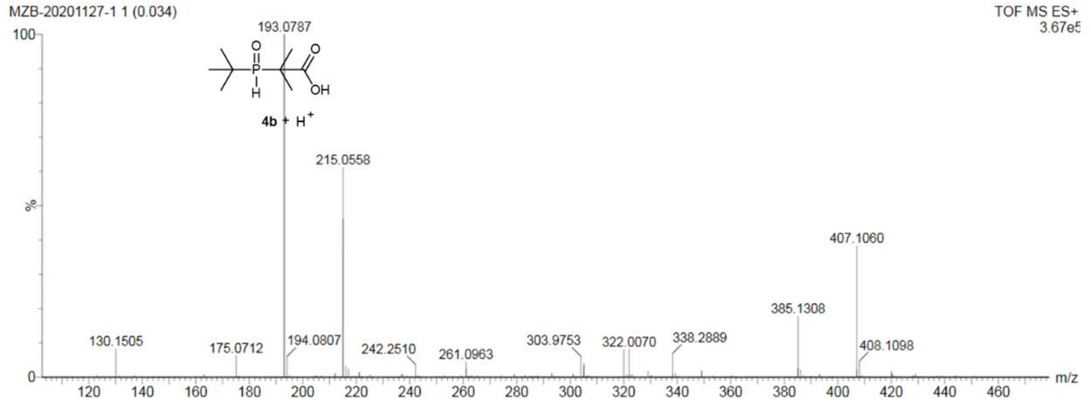

Figure S59. MS spectrum of compound **4b**.

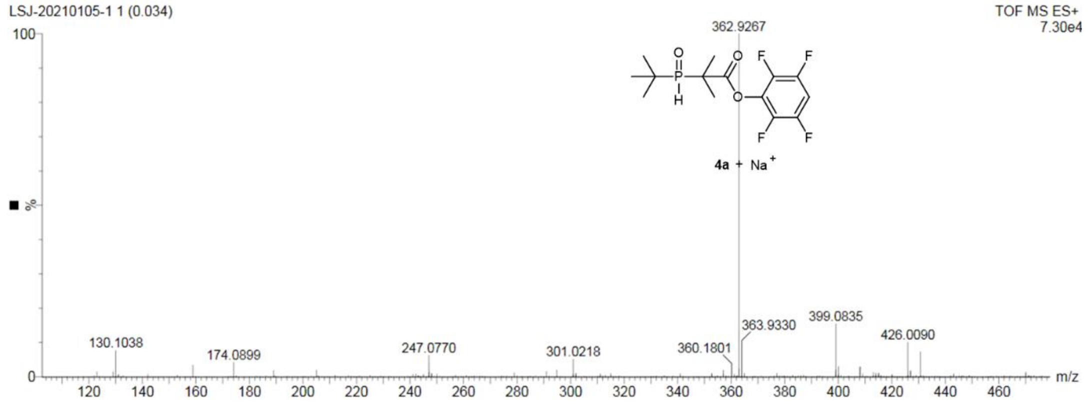

Figure S60. MS spectrum of compound **4a**.

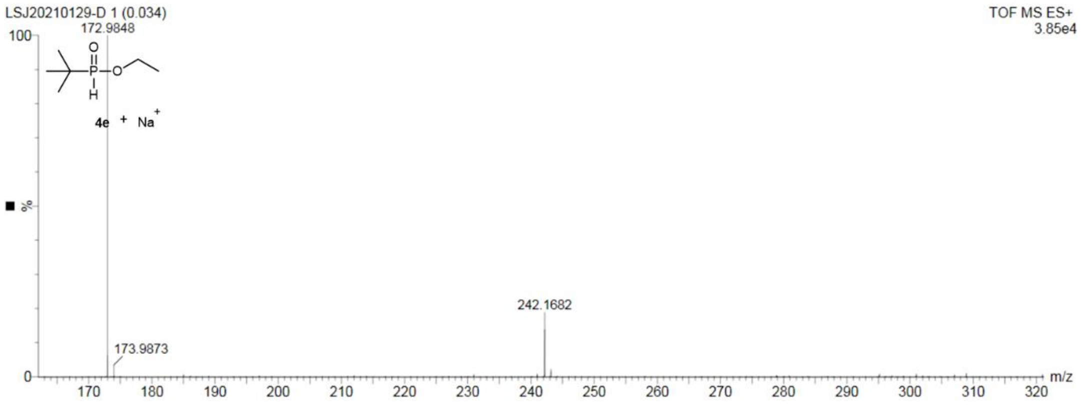

Figure S61. MS spectrum of compound **4e**.

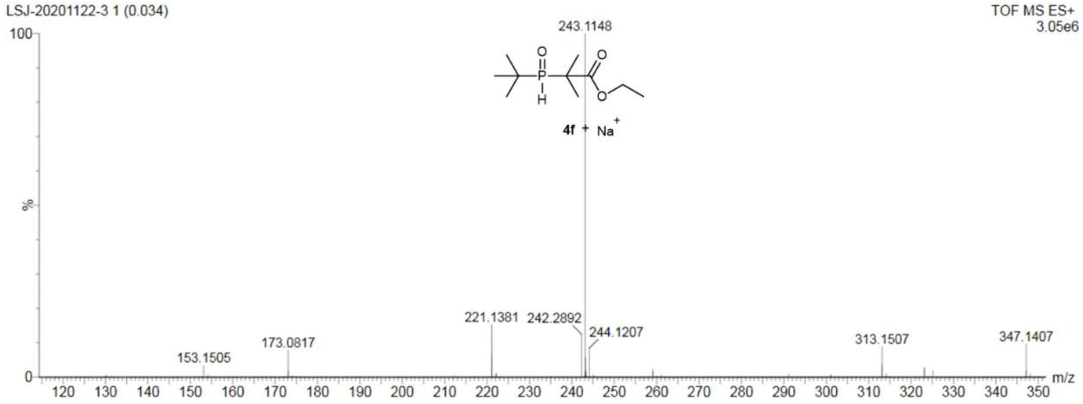

Figure S62. MS spectrum of compound **4f**.

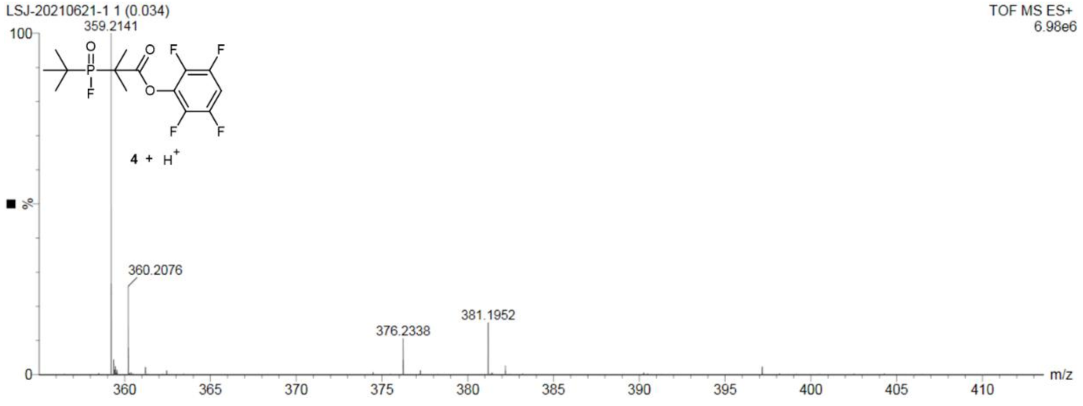

Figure S63. MS spectrum of compound **4**.
